# Competition for Hydride Between Silicon and Boron: Synthesis and Characterization of a Hydroborane‐Stabilized Silylium Ion

**DOI:** 10.1002/chem.202104464

**Published:** 2022-01-10

**Authors:** Haopeng Gao, Robert Müller, Elisabeth Irran, Hendrik F. T. Klare, Martin Kaupp, Martin Oestreich

**Affiliations:** ^1^ Institut für Chemie Technische Universität Berlin Strasse des 17. Juni 115 10623 Berlin Germany

**Keywords:** bidentate interaction, borenium ions, density functional calculations, Lewis acids, silylium ions

## Abstract

Potent main‐group Lewis acids are capable of activating element‐hydrogen bonds. To probe the rivalry for hydride between silylium‐ and borenium‐ion centers, a neutral precursor with the hydrosilane and hydroborane units in close proximity on a naphthalene‐1,8‐diyl platform was designed. Abstraction of one hydride leads to a hydroborane‐stabilized silylium ion rather than a hydrosilane‐coordinated borenium ion paired with [B(C_6_F_5_)_4_]^−^ or [HCB_11_Cl_11_]^−^ as counteranions. Characterization by multinuclear NMR spectroscopy and X‐ray diffraction supported by DFT calculations reveals a cationic, unsymmetrical open three‐center, two‐electron (3c2e) Si−H−B linkage.

## Graphical abstract

**Silylium against borenium**…who wins when tugging on opposite “ends” of hydride? A cationic system based on a naphtalene‐1,8‐diyl platform has been designed for probing the competition for hydride between silicon and boron. The hydroborane‐stabilized silylium ion with a delocalized three‐center, two‐electron B−H−Si bond and more silylium than borenium ion character is the winner (see Scheme; [B(C_6_F_5_)_4_]^−^ omitted for clarity).

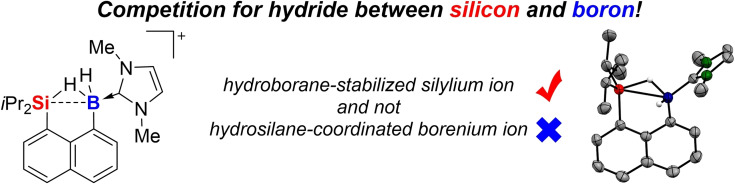

In 1996, Piers reported the ability of the strong boron Lewis acid tris(pentafluoro)phenylborane to catalyze the hydrosilylation of carbonyl compounds.^[1]^ Experimental studies by him^[2]^ and our laboratory^[3]^ along with a subsequent computational analysis^[4]^ indicated that B(C_6_F_5_)_3_ tends to active the Si−H bond of the hydrosilane rather than forming a conventional Lewis adduct with the σ‐basic carbonyl donor.^[5]^ Yet, the assumed borane/hydrosilane intermediate has remained experimentally elusive.^[6]^ Piers, Tuononen and co‐workers eventually achieved the isolation of the related adduct **1** by employing 1,2,3‐tris(pentafluorophenyl)‐4,5,6,7‐tetrafluoro‐1‐boraindene instead of B(C_6_F_5_)_3_ (Scheme 1, top).^[7]^ Since 2014, additional examples of intermolecular Si−H bond activation with Al(C_6_F_5_)_3_ as in **2**,^[8]^ a borenium ion as in **3**[B(C_6_F_5_)_4_]^[9]^ as well as a neutral borane as in **4** ^[10]^ have been disclosed. The understanding of these intermediates is highly relevant to catalysis, especially in the case of Piers’ chemistry.^[11]^ The silicon and boron centers compete for the hydride in these Lewis pairs, resulting in highly interesting bonding situations. Wang's cationic complex **3** ^+^ is a previously unprecedented example of an η^2^‐coordination of the Si−H bond to a Lewis acidic boron atom.

**Scheme 1:**
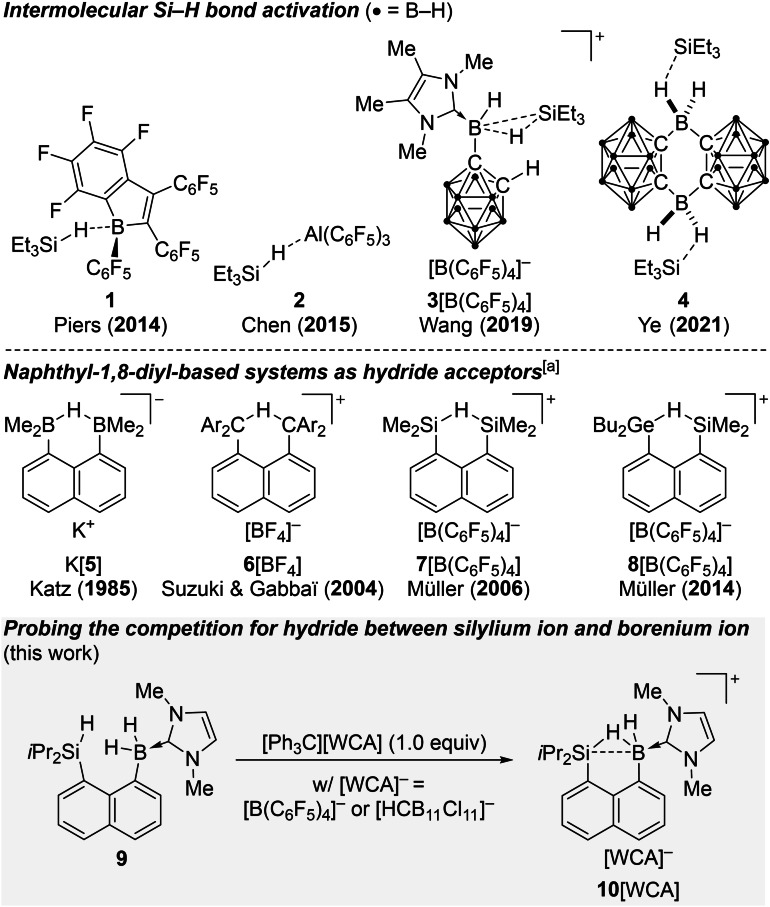
Competition for hydride in hydrosilane/main‐group Lewis acid pairs. [a] The element‐H‐element linkages are shown as symmetric structures but some are in reality nonsymmetric.

To interrogate this “competition for hydride”, we designed the neutral precursor **9** with the Si−H and B−H bonds in the same molecule in close proximity to arrive at the Si/B hydronium ion **10** ^+^ after treatment with the trityl cation (Scheme 1, gray box). Such systems based on the naphthalene‐1,8‐diyl platform have already been utilized by Katz (B/B; K[**5**]),^[12]^ Gabbaï^[13]^ as well as Suzuki^[14]^ (C/C; **6**[BF_4_]), and Müller (Si/Si; **7**[B(C_6_F_5_)_4_])^[15]^ (Scheme 1, bottom). Of note, there is only one example with two different hydride acceptors, that is a Ge/Si hydronium borate **8**[B(C_6_F_5_)_4_] described by Müller and co‐workers for which no crystallographic characterization is available.^[16]^ The key question of our present investigation is whether the Si/B hydronium ion **10** ^+^ is a hydrosilane adduct of a borenium ion or a hydroborane‐stabilized silylium ion. By this, we are bridging our long‐time expertises with Piers‐type chemistry^[11]^ and that of silylium ions.^[17]^

The neutral precursor **9** was synthesized in 24 % yield by lithiation of (8‐bromonaphthalen‐1‐yl)diisopropylsilane, followed by the addition of a toluene suspension of IMe⋅BH_2_I (IMe=1,3‐dimethylimidazol‐2‐ylidene)^[18]^ at −78 °C (see the Supporting Information for details). The *δ*(^11^B) NMR resonance of **9** in C_6_D_6_ appears as a triplet at −23.7 ppm with a ^1^ *J*_B,H_ coupling constant of 87 Hz. This is lowfield relative to *δ*(^11^B) −31.8 ppm for IMe⋅BH_2_I and in the range of arylated NHC‐boranes.^[19]^ The *δ*(^29^Si) NMR signal is observed at 18.9 ppm, and the ^1^ *J*_Si,H_ coupling constant is 182 Hz. Colorless crystals of precursor **9** suitable for X‐ray diffraction were obtained from a concentrated CH_2_Cl_2_/*n*‐hexane solution (2 : 1) at −30 °C overnight (Figure 1).^[20]^ The Si−H bond length of 1.42(2) Å is in the typical range of Si−H bonds (ca. 1.425 Å)^[21]^ and heading away from the boron atom. The distance between the silicon and the boron atoms is 3.19(2) Å, which is longer than the typical range of Si−B single bonds (1.91 Å–2.12 Å)^[22]^ but still within the sum of their van der Waals radii as a result of the steric congestion imposed by the rigid, *peri*‐substituted naphthalene backbone. The repulsion of the silyl and NHC‐boryl moieties can be seen from the deviation of C6−C1−Si1 (130.7(1)°) and C6−C10−B1 (123.0(1)°) angles from the ideal value 120°. Those tight steric constraints likely account for the moderate chemical stability of compound **9** which slowly decomposes within weeks even when kept in the glovebox.

**Figure 1:**
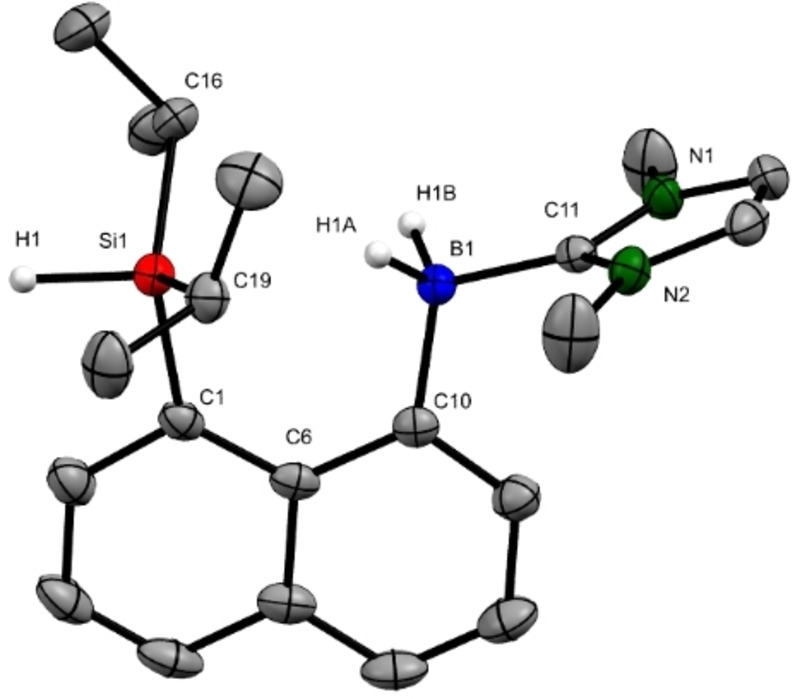
Molecular structure of precursor **9** (thermal ellipsoids are shown at 50 % probability; hydrogen atoms except H1, H1A and H1B are omitted for clarity). Selected bond lengths (Å) and angles (°): Si1−H1 1.42(2), Si1−C16 1.898(1), Si1−C19 1.904(2), Si1−C1 1.904(1), B1−H1A 1.15(2), B1−H1B 1.12(2), B1−C11 1.606(2), Si1⋅⋅⋅B1 3.190(2); Si1−C1−C6 130.7(1), B1−C10−C6 123.0(1), C1−C6−C10 124.2(1).

Treatment of precursor **9** with 1.0 equiv. of [Ph_3_C][B(C_6_F_5_)_4_] in C_6_D_6_ led to a biphasic mixture (Scheme 2). The phases were allowed to separate, and the upper phase was removed. The lower phase containing the cationic product **10**[B(C_6_F_5_)_4_] was washed three times with a few drops of C_6_D_6_ and then dissolved in 1,2‐Cl_2_C_6_D_4_ for NMR spectroscopic characterization. The chemical shift of the silicon atom in the ^29^Si NMR spectrum of **10**[B(C_6_F_5_)_4_] is significantly low‐field shifted compared to the precursor **9** [*δ*(^29^Si) 56.0 ppm versus 18.9 ppm]. Moreover, this value is close to the bissilylhydronium ions with a naphthalene‐1,8‐diyl platform reported by Müller [*δ*(^29^Si) 54.4 ppm for **7** ^+^],^[15a]^ clearly indicating the development of silylium ion character. The broad ^1^H NMR signal at *δ*(^1^H) 2.65 ppm of the bridging hydrogen atom in **10**[B(C_6_F_5_)_4_] is remarkably shifted to high field compared to the Si−H resonance value of 4.82 ppm in **9**. An integration to two protons corroborates that the two boron‐bound hydrides in **10** ^+^ are equivalent due to fast hydrogen exchange process.^[6b]^ This is consistent with the computed very low free energy barrier of only 8 kJ mol^−1^ for this process in solution (at standard conditions; Scheme S1). Due to the line width of the signal, the *J*_Si−H−B_ was not detected in the ^1^H NMR spectrum in 1,2‐Cl_2_C_6_D_4_ at 298 K. The VT NMR showed that the width of signal narrows with decreasing temperature. Thus, the average coupling constant of ^1^ *J*_Si−*H*−B(H)_ and ^3^ *J*_Si−H−B(*H*)_=28 Hz was determined by a ^1^H/^29^Si‐1D‐CLIP‐HSQMBC NMR experiment in ClC_6_D_5_ at 240 K, which is significantly reduced compared to the ^1^ *J*_Si,H_=182 Hz for **9**. The broad signal in ^11^B NMR spectrum shows a lowfield shift to −8.2 ppm relative to **9** [*δ*(^11^B) −23.7 ppm]. A different counteranion was introduced by the reaction of **9** with [Ph_3_C][HCB_11_Cl_11_], furnishing **10**[HCB_11_Cl_11_] with the same chemical shift of *δ*(^29^Si) 56.1 ppm, showing that cation and anion are well‐separated. Attempts to abstract another hydride from **10**[B(C_6_F_5_)_4_] with stoichiometric [Ph_3_C][B(C_6_F_5_)_4_] were unsuccessful even at 80 °C overnight with **10**[B(C_6_F_5_)_4_] remaining intact. This thermal stability underscores the chemical robustness of **10**[B(C_6_F_5_)_4_] whereas Müller's Si/Si system **7**[B(C_6_F_5_)_4_] reacts instantaneously with the weakly coordinating anion [B(C_6_F_5_)_4_]^−^ to afford the corresponding fluorine‐bridged cation.^[15a]^ In stark contrast, an attempt to deprotonate **10**[B(C_6_F_5_)_4_] with KHMDS resulted in decomposition to an intractable mixture.

**Scheme 2:**
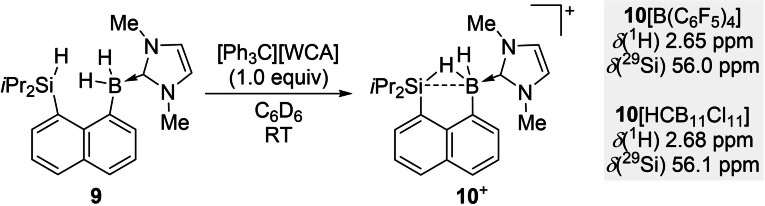
Generation and key ^1^H and ^29^Si NMR resonance signals of the hydroborane‐stabilized silylium ion **10** ^+^ with different counteranions. All NMR data were recorded in 1,2‐Cl_2_C_6_D_4_.

The ion pair **10**[B(C_6_F_5_)_4_] was crystallized at room temperature from a solution in 1,2‐Cl_2_C_6_D_4_ by slow evaporation (Figure 2).^[23]^ Single‐crystal X‐ray diffraction revealed the cationic nature of **10** ^+^, in which both the boron and silicon atoms are tetracoordinated and bridged by a hydrogen atom. The distance between Si1 and B1 is 2.458(2) Å, which is about 25 % longer than the typical range of Si−B single bonds (1.91 Å–2.12 Å)^[22]^ but still within the sum of the van der Waals radii of the silicon and boron atoms. The Si1−B1 distance is remarkably reduced compared to that in the neutral precursor **9** (3.19(2) Å) and also significantly shorter than the Si−B distances in the intermolecular hydrosilane‐activation products **3** ^+^ and **4** (2.570(6) Å and 2.659(14) Å, respectively; see Scheme 1, top). A narrowing of the Si/B−C_peri_−C_bridge_ angles of about 10 % is evidence of a stronger interaction between boron and silicon. The two hydrogen atoms bonded to B1 were located in the difference Fourier map and refined isotropically. One hydrogen atom H1 is bridged between B1 and Si1 atoms, and the B1−H1 distance (1.33(2) Å) is significantly longer than the B1−H2 bond (1.08(2) Å) and close to those B−H−B 3c2e bonds of diborane derivatives also based on naphthalene‐1,8‐diyl platform (1.280(13)–1.310(14) Å)^[24]^ and comparable to those in **3**[B(C_6_F_5_)_4_] and **4** (1.29(5) Å and 1.33(2) Å, respectively). It is worthy of note that the elongation of the Si1−H1 bond in **10**[B(C_6_F_5_)_4_] (1.59(2) Å) to that in **9** (1.42(2) Å) is clearly indicative of the activation of Si−H bond. The Si−H activation degree is comparable to those in **3** ^+^ and **4** (1.59(6) Å and 1.600(16) Å, respectively) and higher than that in **1** (1.51(2) Å) reported by Piers, Tuononen and co‐workers but lower than those in Müller's Si/Si system **7** ^+^ (1.68 Å and 1.58 Å). The sum of all C−Si−C angles (345.5°) confirms a pronounced pyramidalization at the silicon atom, and the B1−H1−Si1 angle (114.1°) is remarkably smaller than those reported Si−H−Si angles in hydride‐bridged disilyl cations[^15a^, ^25^] as well as the B1−H1−Si1 angles in **3** ^+^ and **4** (126(4)° and 130.21°, respectively). To the best of our knowledge, **10**[B(C_6_F_5_)_4_] is the first crystallographically characterized cationic naphthalene‐1,8‐diyl system bearing two different hydride acceptors.

**Figure 2:**
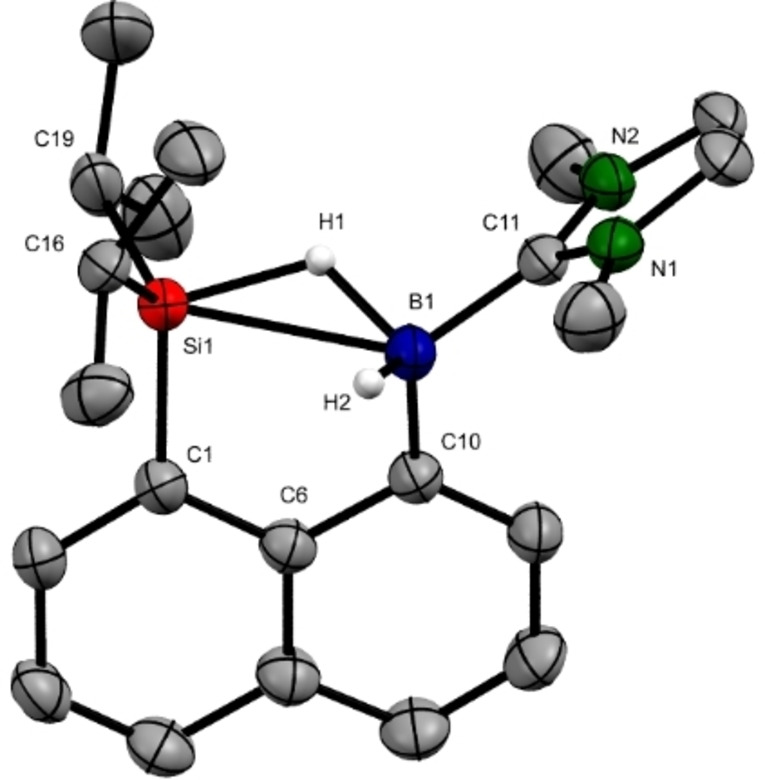
Molecular structure of the hydroborane‐stabilized silylium ion **10** [B(C_6_F_5_)_4_] (thermal ellipsoids are shown at 50 % probability; hydrogen atoms except H1, H2, the counteranion [B(C_6_F_5_)_4_]^−^ and co‐crystallized Li_0.71_Na_0.29_[B(C_6_F_5_)_4_] are omitted for clarity). Selected bond lengths (Å) and angles (°): Si1−H1 1.59(2), Si1−C16 1.866(2), Si1−C19 1.875(3), Si1−C1 1.864(2), Si1−B1 2.458(2), B1−H1 1.33(2), B1−H2 1.08(2), B1−C11 1.586(3); Si1−H1−B1 114(1), C16−Si1−C1 118.46(9), C16−Si1−C19 115.6(1), C19−Si1−C1 111.39(9), Si1−C1−C6 118.2(1), C1−C6−C10 119.9(2), B1−C10−C6 116.6(2), H2−B1−H1104(1), C11−B1−H1 100.9(9), C11−B1−H2 111(1).

Both silylium ions and NHC‐stabilized borenium ions^[26]^ can be generated by hydride abstraction with trityl salts from hydrosilanes and ‐boranes, respectively. To probe whether **10** ^+^ is a hydroborane‐stabilized silylium ion or a borenium‐ion‐activated hydrosilane, quantum chemical calculations using DFT methods were performed (see the Supporting Information for the computational details).

The calculated ^29^Si and ^11^B chemical shifts for the DFT‐optimized structure of **10** ^+^ in 1,2‐Cl_2_C_6_H_4_ (using a continuum solvent model) are in excellent agreement with the experimental values (see Table S4): *δ*(^29^Si) 55.3 ppm and *δ*(^11^B) −8.5 ppm (computed) versus *δ*(^29^Si) 56.0 ppm and *δ*(^11^B) −8.2 ppm for **10**[B(C_6_F_5_)_4_] (experimental). This indicates a correct description of electronic structure details at the chosen computational levels. Of note, the ^11^B chemical shift is particularly sensitive to geometrical distortions in the present case. This provided a good basis for closer analyses of bonding. Natural bond orbital (NBO) and natural resonance theory (NRT) analyses confirm the identification of the B−H−Si moiety in **10** ^+^ as a delocalized 3c2e bond, and the obtained natural bond orders (BOs) are consistent with asymmetrical multicenter σ‐bonding (Figure 3, left and Table S5). Specifically, both a higher total bond order (0.47 versus 0.37) as well as a larger covalent character (0.29 versus 0.19) for the B−H1 bond compared to the Si−H1 bond are found. We also note that the computed Wiberg bond indices show a similar picture (Table S5) but with an even stronger bonding asymmetry in the same direction. The absence of a bond critical point (BCP) between boron and silicon in atoms‐in‐molecules (AIM) analyses does not support the weak B⋅⋅⋅Si interaction suggested by the NRT analyses (BO=0.17), and instead points to the presence of an open Si−H−B 3c2e bond (Figure 3, right). Closely comparable AIM and NBO results were obtained by Wang and co‐workers in their computational studies of **3** ^+^, emphasizing the similarities of B−H−Si multicenter bonding in both compounds despite the different synthetic approaches and molecular compositions.

**Figure 3:**
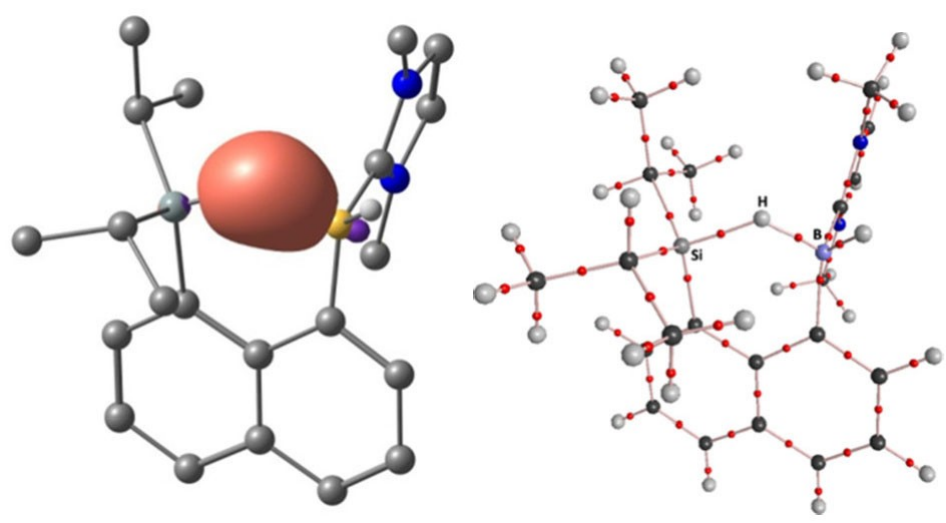
Orbital plot (isosurface value 0.05 a.u.) of the B−H−Si 3c2e NBO in **10** ^+^ (TPSSh/def2‐TZVP) (left) and AIM‐based molecular graph of **10** ^+^ (right).

Based on their results, Wang and co‐workers classified **3** ^+^ as borenium‐ion‐activated hydrosilane. A closer analysis of **10** ^+^ by means of two NBO Lewis structures (LS) featuring either an explicit B−H1 (LS_BH1_) or Si−H1 (LS_SiH1_) σ‐bond with otherwise identical bonding setups (Figure 4) reveals that LS_BH1_ provides a moderately but notably better fit (e. g. a smaller residual non‐Lewis density) of the total density matrix than LS_SiH1_ (3.989e versus 4.125e; Table S7). Compared to the occupancies of the corresponding NBOs in the precursor **9**, substantial charge delocalization from the Si/B−H1 σ‐bond takes place in both cases but significantly more so in LS_SiH1_ (0.47e) than in LS_BH1_ (0.31e). As expected, the predominant acceptor is the (formally) vacant p‐type atomic orbital on the opposite center in each case, which is consequently populated significantly (LS_SiH1_: 0.53e; LS_BH1_: 0.38e). Back‐donation of charge density into the Si/B−H1 σ*‐antibonding orbital is negligible in both cases, which was also observed by Wang and co‐workers for **3** ^+^.

**Figure 4:**
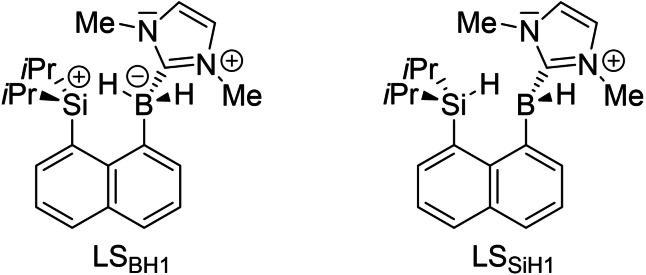
Lewis structures LS_BH1_ (left) and LS_SiH1_ (right) used for NBO analyses, featuring either an explicit B−H1 or Si−H1 bond.

We also estimated the relative Lewis acidity of the silicon and boron centers in **10** ^+^ by computing their fluoride‐ion affinities (FIAs) using F_2_CO as a standard for the appropriate isodesmic reactions (Scheme S2). The results clearly indicate a larger electrophilicity of the silicon atom (644 kJ mol^−1^) compared to the boron atom (609 kJ mol^−1^). Together with essentially all other bonding indicators (see above), this also is consistent with the picture of an open 3c2e Si−H−B bond that tends to be somewhat closer to a hydroborane‐stabilized silylium ion than to a hydrosilane‐stabilized borenium ion.

In conclusion, we presented herein the synthesis of naphthalene‐1,8‐diyl‐based Si/B hydronium ion **10** ^+^ paired with [B(C_6_F_5_)_4_]^−^ and [HCB_11_Cl_11_]^−^ by hydride abstraction from neutral precursor **9**. Ion pairs **10** ^+^ were fully characterized by NMR spectroscopy and X‐ray diffraction. X‐ray crystallography analysis and DFT calculations provide strong evidence for a delocalized 3c2e B−H−Si bond with more pronounced silylium‐ion than borenium‐ion character. The high activation degree of the Si−H bond in **10** ^+^ and the structure of **10**[B(C_6_F_5_)_4_] can be viewed as a snapshot of the “competition for hydride” between two different main‐group element Lewis acid centers, an important feature in Piers‐type chemistry. With an appropriate tether, it may even be possible to synthesize a silylium/borenium dication.^[27]^

## Conflict of interest

The authors declare no conflict of interest.

## Supporting information

As a service to our authors and readers, this journal provides supporting information supplied by the authors. Such materials are peer reviewed and may be re‐organized for online delivery, but are not copy‐edited or typeset. Technical support issues arising from supporting information (other than missing files) should be addressed to the authors.

Supporting Information

## Data Availability

The data that support the findings of this study are available from the corresponding author upon reasonable request.
