# Supplementary material for: Competition for Hydride Between Silicon and Boron: Synthesis and Characterization of a Hydroborane‐Stabilized Silylium Ion
Source: Chemistry. 2022 Jan 10;28(12):e202104464. doi: 10.1002/chem.202104464 (PMC9303568; doi:10.1002/chem.202104464)
Supplement: Supplementary file 1 — Supporting Information [file CHEM-28-0-s001.pdf]

Chemistry–A European Journal

Supporting Information

Competition for Hydride Between Silicon and Boron: Synthesis and Characterization of a Hydroborane-Stabilized Silylium Ion

Haopeng Gao, Robert Müller, Elisabeth Irran, Hendrik F. T. Klare, Martin Kaupp,* and Martin Oestreich*

Table of Contents

**1**	**General Information**	**S2**
**2**	**Experimental Details for the Preparation of Neutral Precursor 9**	**S3**
2.1	(8-Bromonaphthalen-1-yl)diisopropylsilane (**S1**)	S3
2.2	1,3-Dimethylimidazol-2-ylidene iodoborane (IMe·BH_2I)	S4
2.3	Precursor **9**	S4
**3**	**Experimental Details for the Generation of Hydroborane-Stabilized Silylium Ion 10$^+$**	**S6**
3.1	Generation of Si/B Hydronium Borate **10**$^+[B(C_6F_5)_4]^–$	S6
3.2	Generation of Si/B Hydronium Carborate **10**$^+[HCB_{11}Cl_{11}]^–$	S7
**4**	**Variable Temperature NMR Spectroscopic Measurements**	**S8**
**5**	**IR Spectra**	**S10**
**6**	**NMR Spectra**	**S12**
**7**	**Crystallographic Data**	**S45**
7.1	Molecular Structure of Precursor **9**	S45
7.2	Molecular Structure of Si/B Hydronium Borate **10**$^+[B(C_6F_5)_4]^–$	S47
**8**	**Computational Details**	**S49**
**9**	**References**	**S61**

1 General Information

All reactions were performed in flame-dried glassware using an *MBraun* glovebox or conventional Schlenk techniques under a static pressure of argon (glovebox) or nitrogen (fume hood) unless otherwise stated. Standard solvents and reagents were obtained from commercial suppliers and used as received unless otherwise stated. Technical grade solvents for extraction or chromatography (dichloromethane (CH_2Cl_2), ethyl acetate (EtOAc), and *n*-pentane) were distilled prior to use. Diethyl ether (Et_2O) and tetrahydrofuran (THF) were dried over potassium/benzophenone and freshly distilled prior to use. Dry benzene, *n*-hexane, and *n*-pentane were obtained from an *MBraun* solvent purification system (SPS-800), degassed by three freeze-pump-thaw cycles and stored in a glovebox over thermally activated 4 Å molecular sieves. Trityl salt $[Ph_3C][HCB_{11}Cl_{11}]$[S1] and IMe•BH_3 (IMe = 1,3-Dimethylimidazol-2-ylidene)[S2] were synthesized according to reported procedures. Trityl salt $[Ph_3C][B(C_6F_5)_4]$ was purchased from *Boulder Scientific Company* and used as received. Analytical thin-layer chromatography (TLC) was performed on silica gel 60 F254 glass plates. Flash column chromatography was performed on silica gel 60 (40–63 µm, 230–400 mesh ASTM) by *VWR Chemicals* using the indicated solvents. ^{1}H, ^{11}B, ^{13}C, ^{19}F, and ^{29}Si NMR spectra were recorded in C_6D_6, ClC_6D_5, 1,2-$Cl_2C_6D_4$, or $CDCl_3$ on a *Bruker* AV500 and AV700 instrument, respectively. C_6D_6, ClC_6D_5, and 1,2-$Cl_2C_6D_4$ were degassed by three freeze-pump-thaw cycles and stored in a glovebox over thermally activated 4 Å molecular sieves. Chemical shifts are reported in parts per million (ppm) and are referenced to the residual solvent resonance as the internal standard (C_6D_5H: δ 7.16 ppm for ^{1}H NMR and C_6D_6: δ 128.06 ppm for ^{13}C NMR; 1,2-$Cl_2C_6D_3H$: δ 6.94 and 7.20 ppm for ^{1}H NMR and 1,2-$C_6D_4Cl_2$: δ 127.1, 130.1, and 132.5 ppm for ^{13}C NMR; ClC_6D_4H: δ 6.96, 6.99 and 7.14 ppm for ^{1}H NMR; $CHCl_3$: δ 7.26 ppm for ^{1}H NMR and $CDCl_3$: δ 77.16 ppm for ^{13}C NMR). ^{11}B, ^{19}F, and ^{29}Si NMR spectra are referenced in compliance with the unified scale for NMR chemical shifts as recommended by the IUPAC stating the chemical shift relative to $BF_3{\cdot}Et_2O$, CCl_3F, and Me_4Si, respectively.[S3] Data are reported as follows: chemical shift, multiplicity (s = singlet, d = doublet, t = triplet, q = quartet, sept = septet, m = multiplet, m_c = centrosymmetric multiplet, br = broad signal), coupling constants (Hz) and integration. Infrared (IR) spectra were recorded on an *Agilent Technologies* Cary 630 instrument. For air-sensitive compounds, IR spectra were recorded in a glovebox using a *Thermo* Nicolet Magna-IR 750 spectrophotometer equipped with an ATR unit. The signals are reported in wavenumbers (cm^{-1}). High resolution mass spectra (HRMS) were obtained from *the Laboratory of Mass Spectrometry at the Institut für Chemie, Technische Universität Berlin*.

2 Experimental Details for the Preparation of Neutral Precursor 9

2.1 (8-Bromonaphthalen-1-yl)diisopropylsilane (S1)

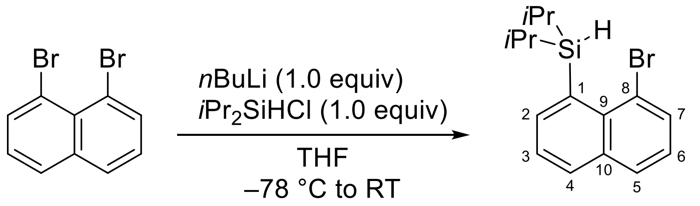

S1
$C_{16}H_{21}BrSi$
M = 321.33 g/mol

n-Butyllithium (0.98 mL of a 2.5 M solution in *n*-hexane, 2.45 mmol, 1.0 equiv) was added dropwise to a solution of 1,8-dibromonaphthalene (0.70 g, 2.45 mmol, 1.0 equiv) in THF (10 mL) at –78 °C. After complete addition, the mixture was stirred at this temperature for another 1 h. Chlorodiisopropylsilane (0.36 mL, 0.319 g, 2.45 mmol, 1.0 equiv) was added dropwise at –78 °C, and the reaction mixture was allowed to slowly warm to room temperature overnight. The reaction was quenched by the addition of saturated aqueous NH_4Cl solution (10 mL). The phases were separated, and the aqueous phase was extracted with EtOAc (3 × ~20 mL). The combined organic phases were dried over anhydrous Na_2SO_4, and the solvent was removed under vacuum. The residue was purified by flash column chromatography on silica gel using *n*-pentane as eluent to afford (8-bromonaphthalen-1-yl)diisopropylsilane (**S1**) as a colorless oil (0.71 g, 90% yield).

IR (ATR): ṽ = 3159, 3052, 2937. 2858, 2716, 2609, 2358, 2080 (s, Si–H), 1593, 1491, 1459, 1380, 1304, 1233, 1191, 1144, 1071, 1006, 973, 876, 811, 763, 707, 662 cm^{-1}. **^{1}H NMR** (500 MHz, $CDCl_3$, 298 K): δ = 0.89 (d, *J* = 7.4 Hz, 6H, $CH(CH_3)_2$), 1.20 (d, *J* = 7.4 Hz, 6H, $CH(CH_3)_2$), 1.64 (m_c, 2H, $CH(CH_3)_2$), 4.18 (br, 1H, Si*H*), 7.29 (dd, *J* = 7.8 Hz, 1H, 6-H_{Ar}), 7.44 (dd, *J* = 7.8 Hz, 1.15 Hz, 1H, 3-H_{Ar}), 7.82 (dd, *J* = 7.8 Hz, 1.2 Hz, 1H, 7-H_{Ar}), 7.86 (m, *J* = 7.6 Hz, 2H, 4,5-H_{Ar}), 8.09 (d, *J* = 6.8 Hz, 1H, 2-H_{Ar}) ppm. **^{13}C{^{1}H} NMR** (101 MHz, $CDCl_3$, 298 K): δ = 15.7 ($CH(CH_3)_2$), 20.2 ($CH(CH_3)_2$), 20.4 ($CH(CH_3)_2$), 123.3 (8-C_{Ar}), 125.5 (3-C_{Ar}), 125.8 (6-C_{Ar}), 129.7 (7-C_{Ar}), 131.4 (4-C_{Ar}), 131.9 (5-C_{Ar}), 135.1 (1-C_{Ar}), 136.1 (10-C_{Ar}), 136.8 (9-C_{Ar}), 142.1 (2-C_{Ar}) ppm. **^{1}H/^{29}Si HMQC NMR** (500/99 MHz, $CDCl_3$, 298 K, optimized for *J* = 7 Hz): δ = 0.89/25.8, 1.20/25.8 ppm. **HRMS** (APCI): calculated for $C_{16}H_{20}BrSi^{\bullet+}$ $[M–H]^{\bullet+}$: 319.0518; found 319.0510.

2.2 1,3-Dimethylimidazol-2-ylidene iodoborane (IMe·BH2I)

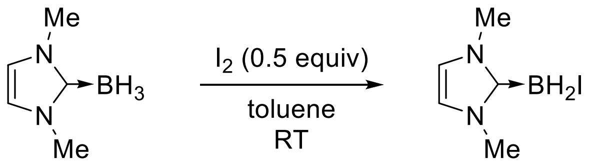

A solution of iodine (0.29 g, 1.14 mmol, 0.5 equiv) in toluene (5 mL) was added dropwise to a suspension of IMe·BH_3[S2] (0.25 g, 2.27 mmol, 1.0 equiv) in toluene (10 mL) at room temperature. Gas evolution was observed upon addition. After stirring the resulting orange suspension at room temperature for another 1 h, the solvent was removed under vacuum to yield IMe·BH_2I as a light-yellow solid (0.53 g, quantitative).

^{1}H NMR (500 MHz, C_6D_6, 298 K): δ = 2.90 (s, 6H, CH_3), 5.35 (s, 2H, NCHCHN) ppm. **^{11}B NMR** (161 MHz, C_6D_6, 298 K): δ = –31.8 (t, $^1J_{B,H}$ = 128 Hz) ppm. **^{13}C{^{1}H} NMR** (126 MHz, C_6D_6, 298 K): δ = 34.9 (CH_3), 119.8 (CH) ppm. The NMR spectroscopic data are in accordance with those reported.[S4]

2.3 Precursor 9

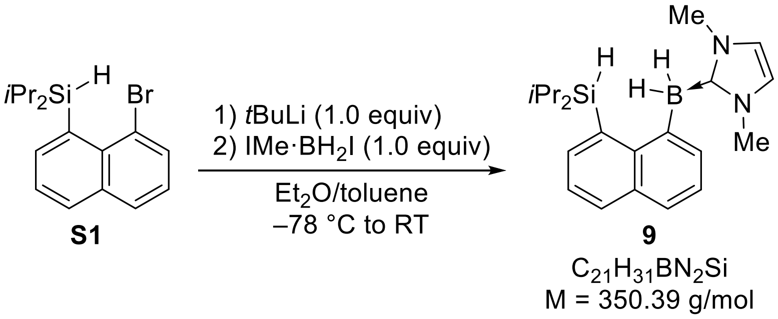

tert-Butyllithium (0.25 mL of a 1.9 M solution in *n*-pentane, 0.47 mmol, 1.0 equiv) was added dropwise to a solution of (8-bromonaphthalen-1-yl)diisopropylsilane (**S1**, 150 mg, 0.47 mmol, 1.0 equiv) in Et_2O (10 mL) at –78 °C. After stirring at this temperature for another 1 h, a suspension of IMe·BH_2I (109 mg, 0.47 mmol, 1.0 equiv) in toluene (5 mL) precooled to –78 °C was transferred to the reaction mixture using a cannula. The reaction mixture was allowed to slowly warm to room temperature overnight. The Et_2O of the resulting white suspension was removed under vacuum, CH_2Cl_2 (8 mL) was added, and the suspension was filtered using a filter cannula. The remaining white residue was extracted with toluene (2 × ~5 mL) and filtered. The filtrates were combined and filtered again. The solvent was removed under vacuum, and the resulting colorless viscous residue was washed with *n*-hexane (3 × ~2 mL) and dried under vacuum to afford precursor **9** as a white solid (40 mg, 24%). Colorless crystals of **9** suitable for X-ray diffraction analysis were obtained from a concentrated CH_2Cl_2/*n*-hexane (2:1) solution at –30 °C (see section 7.1). CCDC 2116775 contains the supplementary crystallographic data

for precursor **9**. These data are provided free of charge by The Cambridge Crystallographic Data Centre.

IR (ATR): ṽ = 2938, 2884, 2857, 2361 (B–H), 2300 (B–H), 2054 (Si–H), 1572, 1479, 1440, 1379, 1299, 1229, 1210, 1198, 1170, 1155, 1142, 1092, 1080, 1051, 1006, 994, 980, 878, 872, 803, 774, 752, 734, 724, 670, 633, 614, 591, 585. **^{1}H NMR** (500 MHz, C_6D_6, 298 K): δ = 1.30 (d, ^3J = 7.4 Hz, 6H, CH(C*H*$_3$)$_2$), 1.48 (d, ^3J = 7.4 Hz, 6H, CH(C*H*$_3$)$_2$), 2.0 (br, 2H, C*H*(CH$_3$)$_2$), 2.84 (s, 6H, CH$_3$), 3.00–3.50 (q, $^1J_{B,H}$ = 87 Hz, 2H, BH$_2$), 4.82 (br, $^1J_{Si,H}$ = 182 Hz, 1H, SiH), 5.54 (s, 2H, NCHCHN), 7.00 (br, 1H, 7-H$_{Ar}$), 7.33 (dd, J = 7.0 Hz, 0.8 Hz, 1H, 6-H$_{Ar}$), 7.40 (dd, J = 7.0 Hz, 0.8 Hz, 1H, 3-H$_{Ar}$), 7.71 (d, J = 8.0 Hz, 1H, 5-H$_{Ar}$), 7.87 (dd, J = 8.0 Hz, 1.0 Hz, 1H, 4-H$_{Ar}$), 8.30 (d, J = 6.8 Hz, 1H, 2-H$_{Ar}$) ppm. **^{11}B NMR** (161 MHz, C_6D_6, 298 K): δ = –23.7 (t, $^1J_{B,H}$ = 87 Hz) ppm **^{13}C{^{1}H} NMR** (225 MHz, C_6D_6, 298 K): δ = 15.6 (*C*H(CH$_3$)$_2$), 20.2 (CH(*C*H$_3$)$_2$), 20.7 (CH(*C*H$_3$)$_2$), 35.0 (CH$_3$), 119.2 (NCHCHN), 123.4 (3-C$_{Ar}$), 124.5 (6-C$_{Ar}$), 126.4 (5-C$_{Ar}$), 131.4 (4-C$_{Ar}$), 131.7 (7-C$_{Ar}$), 134.9 (10-C$_{Ar}$), 137.5 (1-C$_{Ar}$), 138.3* (2-C$_{Ar}$), 144.3 (9-C$_{Ar}$), 152.5* (8-C$_{Ar}$), 173.0* (C$_{carbene}$) ppm (*Determined by a ^{1}H/^{13}C HMBC NMR experiment). **^{1}H/^{29}Si HMQC NMR** (500/99 MHz, C_6D_6, 298 K, optimized for J = 7 Hz): δ = 1.30/18.9, 1.48/18.9 ppm. **^{1}H/^{29}Si HMQC NMR** (500/99 MHz, C_6D_6, 298 K, optimized for J = 200 Hz): δ = 4.82/18.9 ppm. **HRMS** (APCI): calculated for $C_{21}H_{28}BN_2Si_2^{\bullet+}$ [M–3H]$^{\bullet+}$: 347.2115; found 347.2123.

3 Experimental Details for the Generation of Hydroborane-Stabilized Silylium Ion 10⁺

3.1 Generation of Si/B Hydronium Borate $10^+[B(C_6F_5)_4]^-$

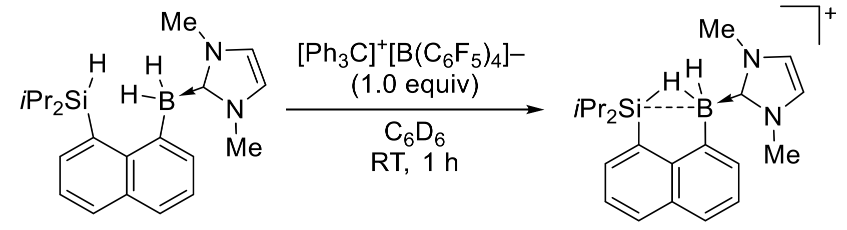

9

10$^+[B(C_6F_5)_4]^-$ $C_{45}H_{30}B_2F_{20}N_2Si$
M = 1028.42 g/mol

Precursor **9** (12.8 mg, 0.037 mmol, 1.00 equiv) was dissolved in C_6D_6 (~15 drops) and added to a suspension of $[Ph_3C][B(C_6F_5)_4]$ (33.7 mg, 0.037 mmol, 1.00 equiv) in C_6D_6 (5 drops). After stirring at room temperature for 1 h, the biphasic mixture was allowed to separate. The upper phase was removed, and the oily lower phase was washed with C_6D_6 (3 × ~5 drops). The residue was dissolved in 1,2-$Cl_2C_6D_4$ and directly subjected to NMR spectroscopic measurements, revealing the formation of Si/B hydronium borate **10**$^+[B(C_6F_5)_4]^-$. Yellow crystals suitable for X-ray diffraction analysis were obtained by slow evaporation from a 1,2-$Cl_2C_6D_4$ solution at room temperature (see section 7.2). CCDC 2116777 contains the supplementary crystallographic data for **10**$^+$. These data are provided free of charge by The Cambridge Crystallographic Data Centre.

^{1}H NMR (500 MHz, 1,2-$Cl_2C_6D_4$, 298 K): δ = 0.95 (d, ^3J = 7.5 Hz, 6H, CH(CH_3)$_2$), 0.98 (d, ^3J = 7.5 Hz, 6H, CH(CH_3)$_2$), 1.58 (sept, ^3J = 7.5 Hz, 2H, CH(CH$_3$)$_2$), 2.65 (br, 2H, SiHB(H)), 3.33 (s, 6H, CH$_3$), 6.71 (s, 2H, NCHCHN), 6.91 (overlapping, 1H, 7-H$_{Ar}$), 7.35 (dd, J = 6.9 Hz, J = 1.1 Hz, 1H, 6-H$_{Ar}$), 7.49 (dd, J = 6.9 Hz, J = 1.1 Hz, 1H, 3-H$_{Ar}$), 7.55 (dd, J = 6.8 Hz, J = 1.1 Hz, 1H, 2-H$_{Ar}$), 7.71 (d, J = 8.0 Hz, 1H, 5-H$_{Ar}$), 7.91 (d, J = 8.2 Hz, 1H, 4-H$_{Ar}$) ppm. **^{11}B NMR** (161 MHz, 1,2-$Cl_2C_6D_4$, 298 K): δ = –8.2 (br, SiHB(H)), –16.2 (s, $[B(C_6F_5)_4]^-$) ppm. **^{11}B{^{1}H} NMR** (161 MHz, 1,2-$Cl_2C_6D_4$, 298 K): δ = –8.2 (br, SiHB(H)), –16.2 (s, $[B(C_6F_5)_4]^-$) ppm. **^{13}C{^{1}H} NMR** (126 MHz, 1,2-$Cl_2C_6D_4$, 298 K): δ = 15.2 (CH(CH$_3$)$_2$), 17.2 (CH(CH$_3$)$_2$), 17.3 (CH(CH$_3$)$_2$), 36.6 (CH$_3$), 123.9 (NCHCHN), 125.5 (3-C$_{Ar}$), 126.6 (6-C$_{Ar}$), 126.7 (1-C$_{Ar}$), 128.6 (5-C$_{Ar}$), 129.3 (7-C$_{Ar}$), 130.2 (8-C$_{Ar}$), 132.1 (2-C$_{Ar}$), 132.5 (4-C$_{Ar}$), 132.9 (10-C$_{Ar}$), 135.6 (C$_{Ar}$-$[B(C_6F_5)_4]^-$), 137.5 (C$_{Ar}$-$[B(C_6F_5)_4]^-$), 139.4 (C$_{Ar}$-$[B(C_6F_5)_4]^-$), 141.0 (9-C$_{Ar}$), 149.7 (C$_{Ar}$-$[B(C_6F_5)_4]^-$), 152.4* (C$_{carbene}$) ppm (*Determined by a ^{1}H/^{13}C HMBC NMR experiment). **^{19}F NMR** (471 MHz, 1,2-$Cl_2C_6D_4$, 298 K): δ –166.0 (t, J = 20.9 Hz), –162.1 (t, J = 21.5 Hz), –131.7 (br) ppm. **^{1}H/^{29}Si HMQC NMR** (500/99 MHz, 1,2-$Cl_2C_6D_4$, 298 K, optimized for J = 7 Hz): δ = 1.00–1.05/56.0, 7.60/56.0 ppm **^{1}H/^{29}Si HMQC NMR** (500/99 MHz, 1,2-$Cl_2C_6D_4$, 298 K, optimized for J = 45 Hz): δ = 1.00–1.05/56.0, 2.65/56.0 ppm. **^{1}H/^{29}Si HMQC NMR** (500/99 MHz, 1,2-$Cl_2C_6D_4$, 298 K, optimized

for *J* = 200 Hz): δ = 1.00–1.05/56.0, 2.65/56.0 ppm. **^{1}H/^{29}Si-1D-CLIP-HSQMBC NMR** (500/99 MHz, ClC_6D_5, 240 K): 7.57 (d, $J_{Si,H}$ = 10.2 Hz), 2.64 (d, $J_{Si–H–B(H)}$ = 28.0 Hz), 1.40 (m), 1.01–1.03 (d, $J_{Si,H}$ = 10.2 Hz), 0.98–1.00 (d, $J_{Si,H}$ = 10.2 Hz) ppm.

3.2 Generation of Si/B Hydronium Carborate $10^+[HCB_{11}Cl_{11}]^–$

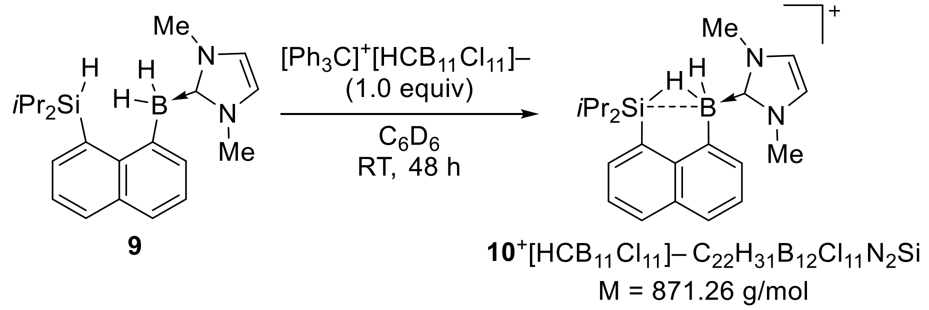

Precursor **9** (14.0 mg, 0.04 mmol, 1.00 equiv) was dissolved in C_6D_6 (~15 drops) and added to a suspension of $[Ph_3C][HCB_{11}Cl_{11}]$ (30.6 mg, 0.04 mmol, 1.00 equiv) in C_6D_6 (10 drops). After stirring at room temperature for 48 h, *n*-pentane (0.5 mL) was added to the white suspension to ensure that all ionic products had precipitated. The precipitate was collected by decantation, washed with C_6D_6 (~5 drops) and *n*-pentane (3 × ~5 drops), and briefly dried under vacuum to afford Si/B hydronium carborate **10**$^+[HCB_{11}Cl_{11}]^–$ as an off-white solid (26 mg, 75%).

IR (ATR): ṽ = 3150, 3119, 2922, 2486 (B–$H_{terminal}$), 2457 (B–$H_{terminal}$), 1607, 1567, 1486, 1436, 1408, 1347, 1229, 1168, 1146, 1101, 1080, 971, 894, 757, 712, 616, 586, 581, 570, 561. **^{1}H NMR** (500 MHz, 1,2-$Cl_2C_6D_4$, 298 K): δ = 0.96 (d, ^3J = 7.5 Hz, 6H, CH(CH_3)$_2$), 0.98 (d, ^3J = 7.5 Hz, 6H, CH(CH_3)$_2$), 1.48 (sept, ^3J = 7.5 Hz, 2H, C*H*(CH_3)$_2$), 2.68 (br, 2H, SiHB(H)), 3.01 (s, 1H, $[HCB_{11}Cl_{11}]^-$), 3.46 (s, 6H, CH_3), 6.90 (s, 2H, NCHCHN), 6.95 (overlapping, 1H, 7-H_{Ar}) 7.35 (t, *J* = 7.8 Hz, 1H, 6-H_{Ar}), 7.49 (t, *J* = 7.9 Hz, 1H, 3-H_{Ar}), 7.56 (d, *J* = 6.8 Hz, 1H, 2-H_{Ar}), 7.70 (d, *J* = 8.2 Hz, 1H, 5-H_{Ar}), 7.90 (d, *J* = 8.2 Hz, 1H, 4-H_{Ar}). **^{11}B NMR** (161 MHz, 1,2-$Cl_2C_6D_4$, 298 K): δ = –12.6 (s, $B_{carborate}$), –9.5 (s, SiHB(H) and $B_{carborate}$), –2.0 (s, $B_{carborate}$) ppm. **^{13}C{^{1}H} NMR** (126 MHz, 1,2-$Cl_2C_6D_4$, 298 K): δ = 15.2 (*C*H(CH_3)$_2$), 17.2 (CH(*C*H_3)$_2$), 37.0 (CH_3), 47.4 ($C_{carborane}$), 124.3 (NCHCHN), 125.5 (3-C_{Ar}), 126.6 (6-C_{Ar}), 126.7(1-C_{Ar}), 128.6 (5-C_{Ar}), 129.4 (7-C_{Ar}), 130.3 (8-C_{Ar}),132.0 (2-C_{Ar}), 132.5 (4-C_{Ar}), 132.9 (10-C_{Ar}),141.0 (9-C_{Ar}), 152.1* ($C_{carbene}$) ppm (*Determined by a ^{1}H/^{13}C HMBC NMR experiment). **^{1}H/^{29}Si HMQC NMR** (500/99 MHz, 1,2-$Cl_2C_6D_4$, 298 K, optimized for *J* = 7 Hz): δ = 0.95–1.00/56.1, 7.57/56.1 ppm. **^{1}H/^{29}Si HMQC NMR** (500/99 MHz, 1,2-$Cl_2C_6D_4$, 298 K, optimized for *J* = 45 Hz): δ = 0.95–1.00/56.1 ppm. **^{1}H/^{29}Si HMQC NMR** (500/99 MHz, 1,2-$Cl_2C_6D_4$, 298 K, optimized for *J* = 200 Hz): δ = 0.95–1.00/56.1 ppm.

4 Variable Temperature NMR Spectroscopic Measurements

Variable temperature NMR spectroscopic analysis was performed with Si/B hydronium borate **10**$^+$[B(C_6F_5)$_4$]$^-$ in 1,2-$Cl_2C_6D_4$. Due to the melting point of 1,2-$Cl_2C_6D_4$ (256 K), the measurements were conducted between 260 K and 323 K. In all cases, the NMR spectra were similar, providing no indication of a change in the stabilization mode of Si/B hydronium borate **10**$^+$[B(C_6F_5)$_4$]$^-$. The line width of the signal of the bridging hydrogen atoms in Si–H–B(H) at δ(^{1}H) 2.6–2.7 ppm narrows with decreasing temperature. The average coupling constant of $^1J_{Si–H–B(H)}$ and $^3J_{Si–H–B(H)}$ = 28 Hz was determined by a ^{1}H/^{29}Si-1D-CLIP-HSQMBC NMR experiment in ClC_6D_5 at 240 K, which is significantly reduced compared to $^1J_{Si,H}$ = 182 Hz for precursor **9**.

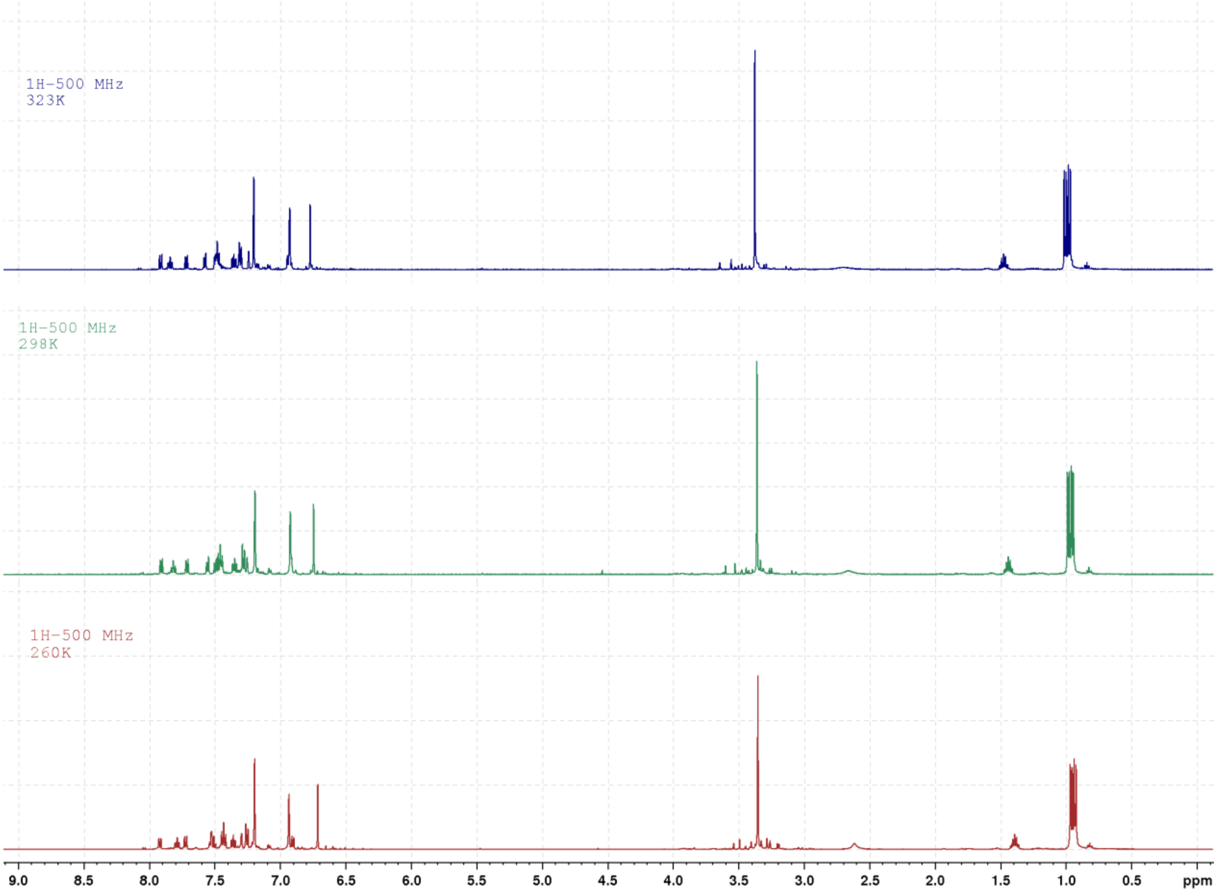

Figure S1. ^{1}H NMR spectra (500 MHz, 1,2-$Cl_2C_6D_4$) of Si/B hydronium borate **10**$^+$[B(C_6F_5)$_4$]$^-$ at 323 K (blue, top), 298 K (green, middle), and 260 K (red, bottom).

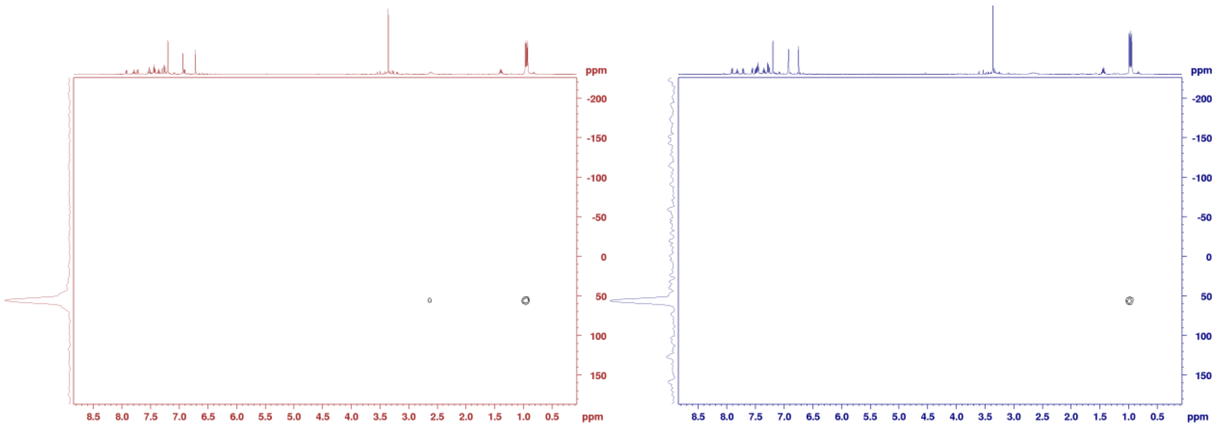

Figure S2. ^{1}H/^{29}Si HMQC NMR spectra (500/99 MHz, 1,2-$Cl_2C_6D_4$, optimized for J = 45 Hz) of Si/B hydronium borate **10**$^+$[B(C_6F_5)$_4$]$^–$ at 323 K (blue, right) and 260 K (red, left).

5 IR Spectra

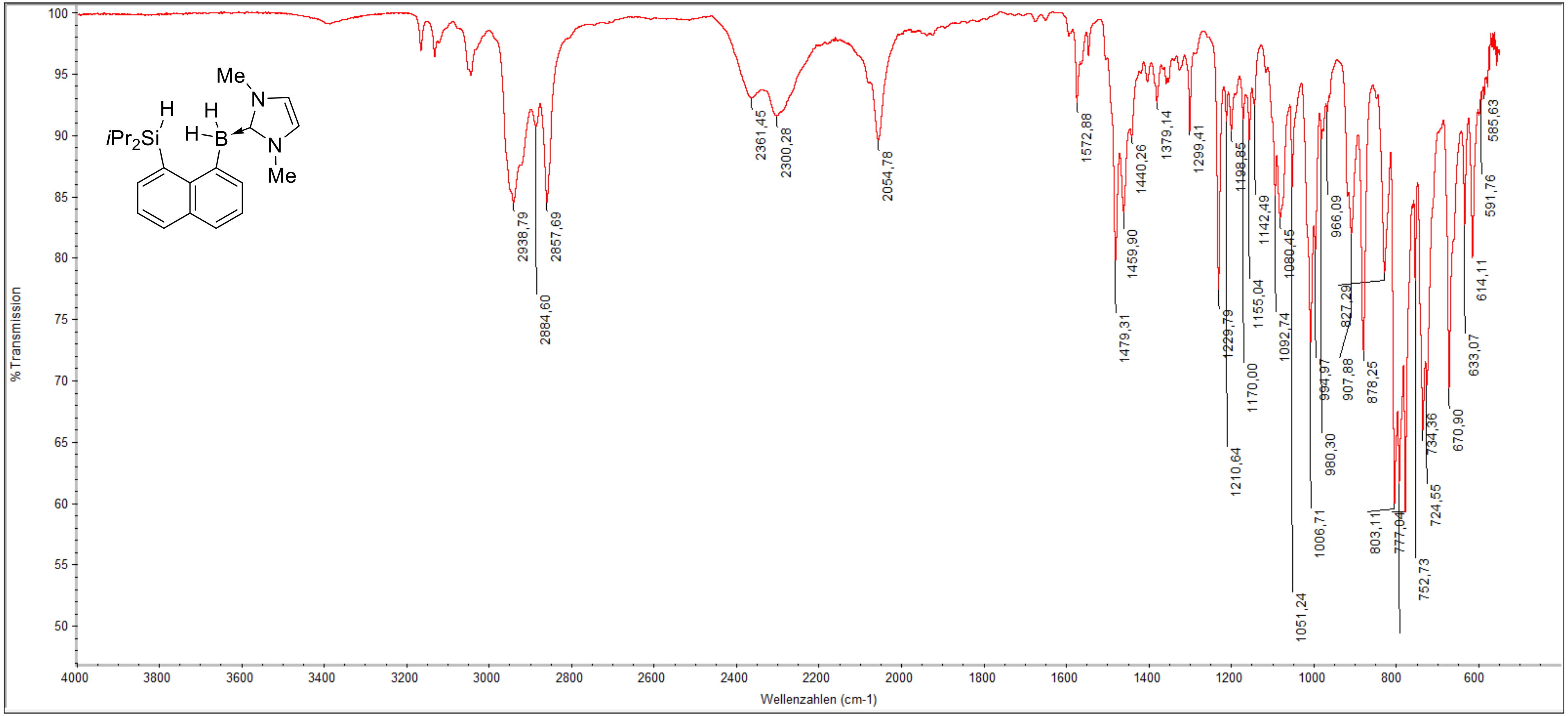

Figure S3. IR spectrum (ATR) of precursor **9**.

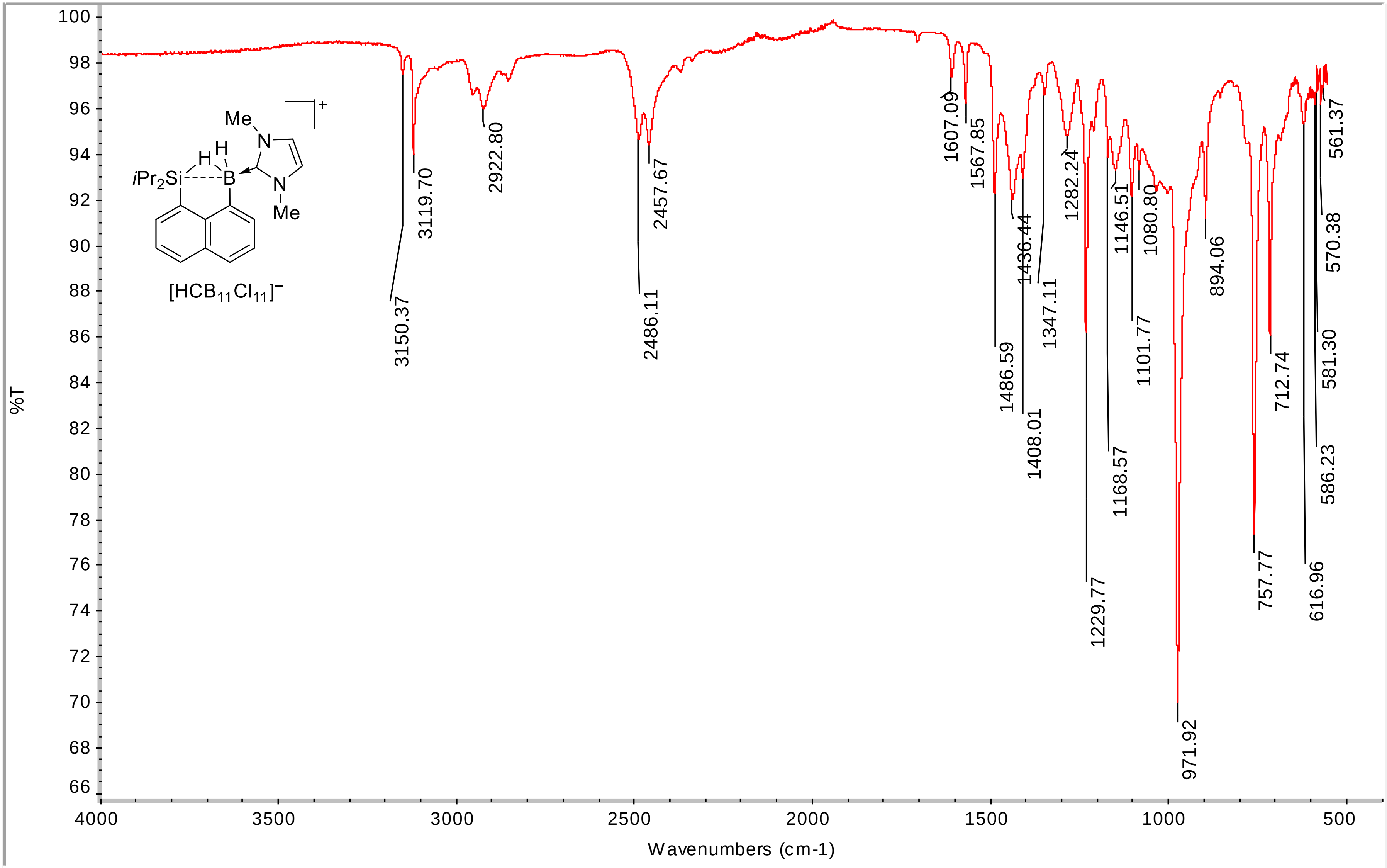

Figure S4. IR spectrum (ATR) of Si/B hydronium carborate **10**$^{+}$[$HCB_{11}Cl_{11}$]$^{-}$.

6 NMR Spectra

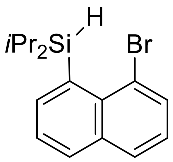

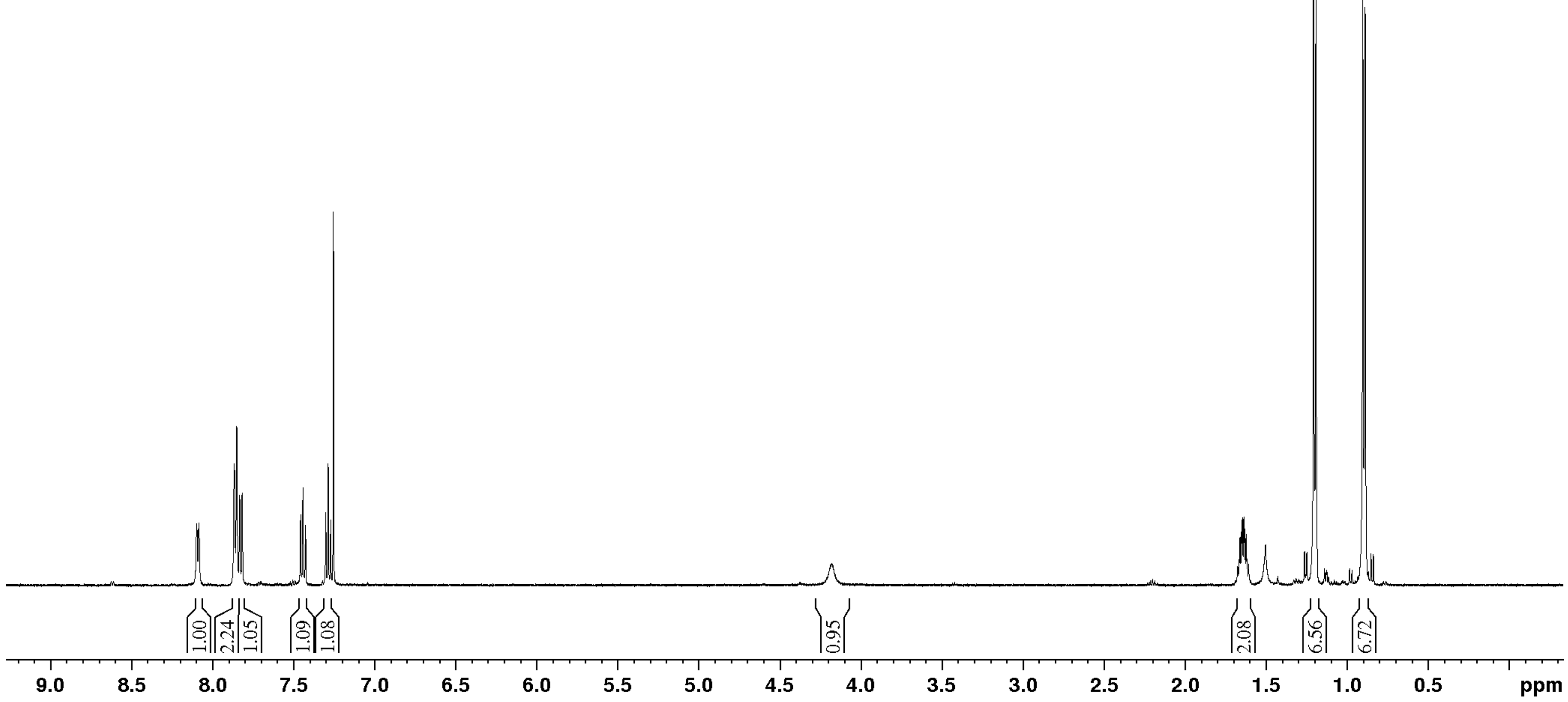

Figure S5. ^{1}H NMR spectrum (500 MHz, $CDCl_3$, 298 K) of (8-bromonaphthalen-1-yl)diisopropylsilane (**S1**).

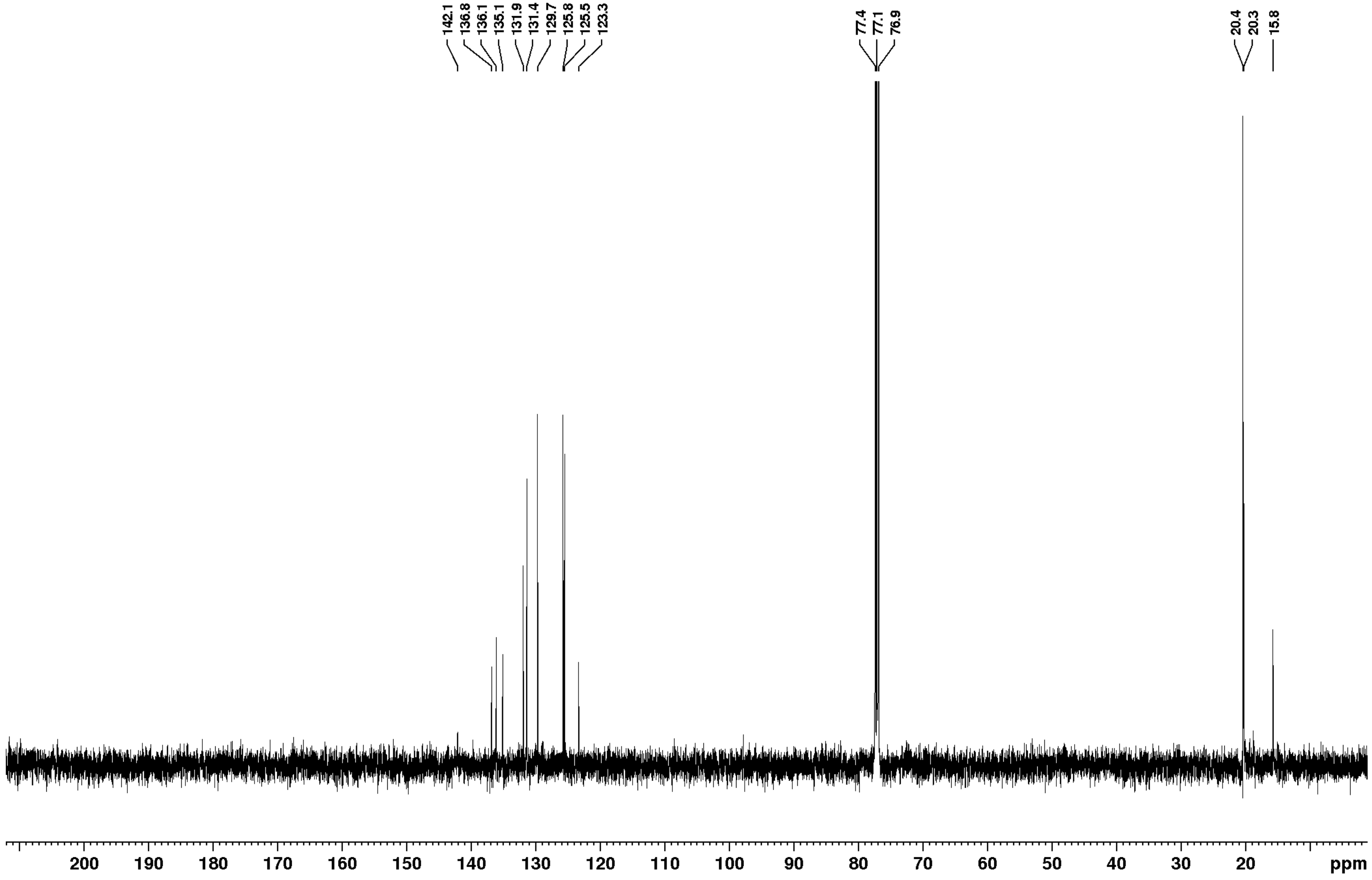

Figure S6. $^{13}C\{^{1}H\}$ NMR spectrum (126 MHz, $CDCl_3$, 298 K) of (8-bromonaphthalen-1-yl)diisopropylsilane (**S1**).

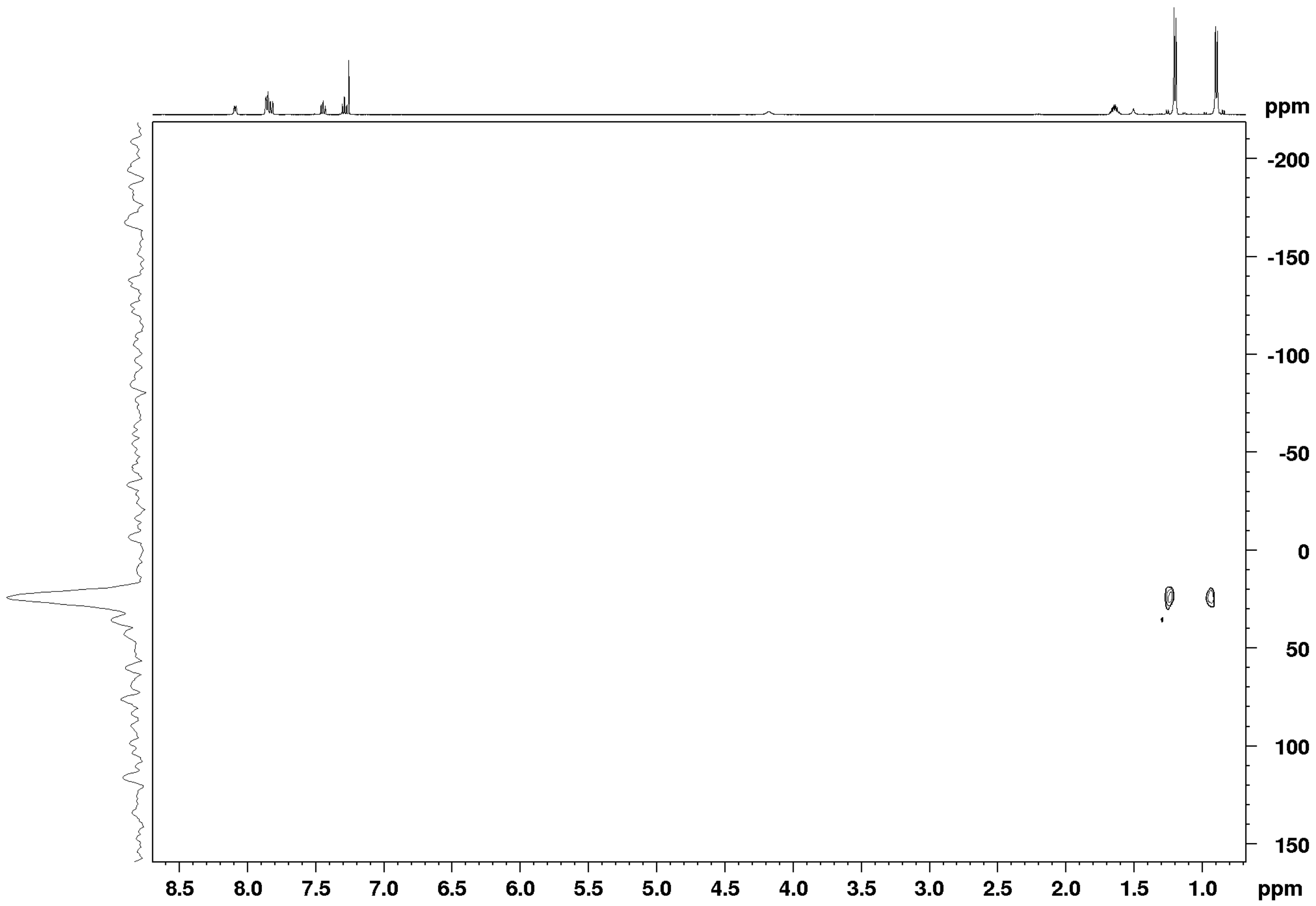

Figure S7. $^{1}H/^{29}Si$ HMQC NMR (500/99 MHz, $CDCl_3$, 298 K, optimized for J = 7 Hz) of (8-bromonaphthalen-1-yl)diisopropylsilane (**S1**).

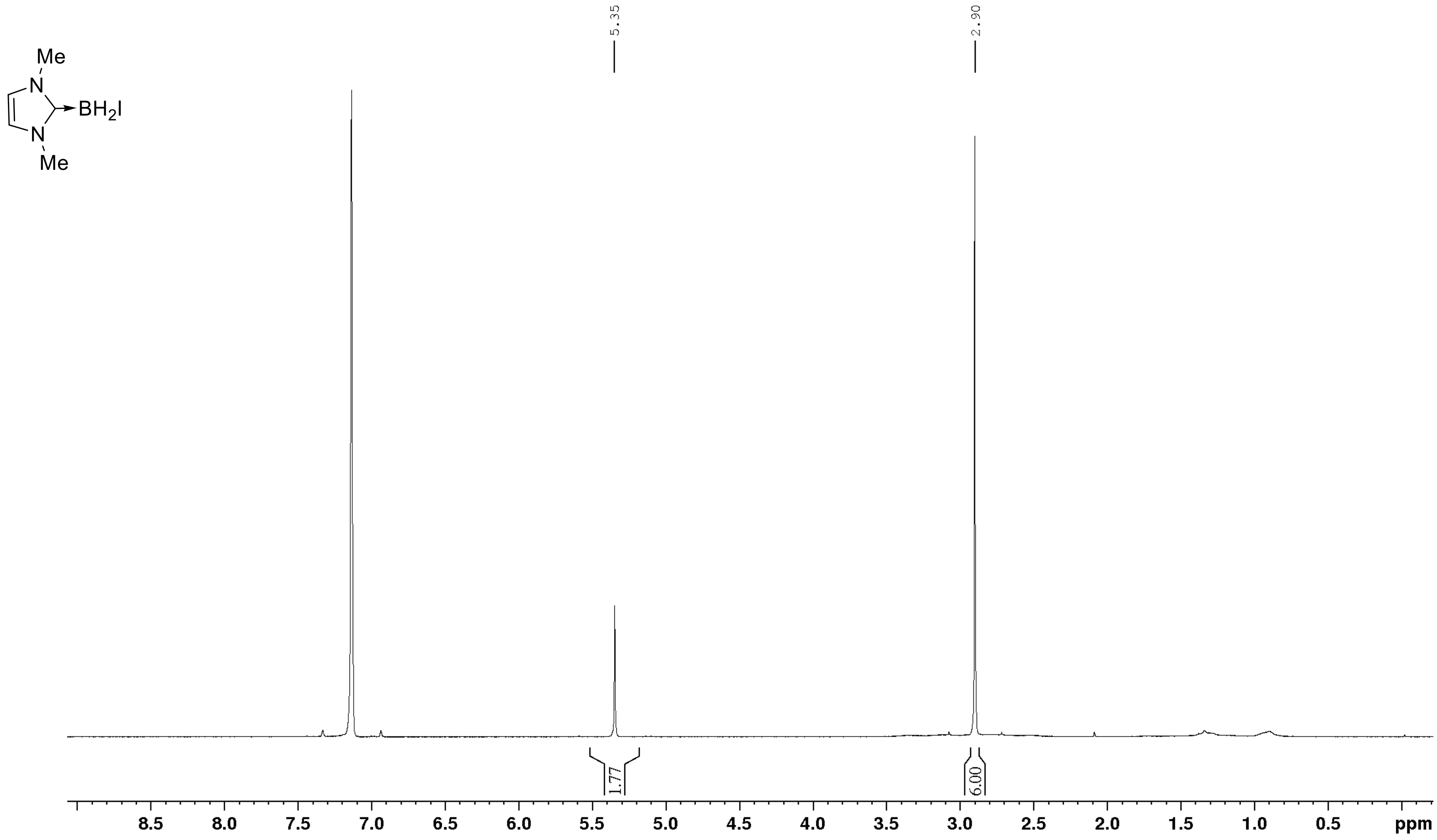

Figure S8. ^{1}H NMR spectrum (500 MHz, C_6D_6, 298 K) of IMe·BH_2I.

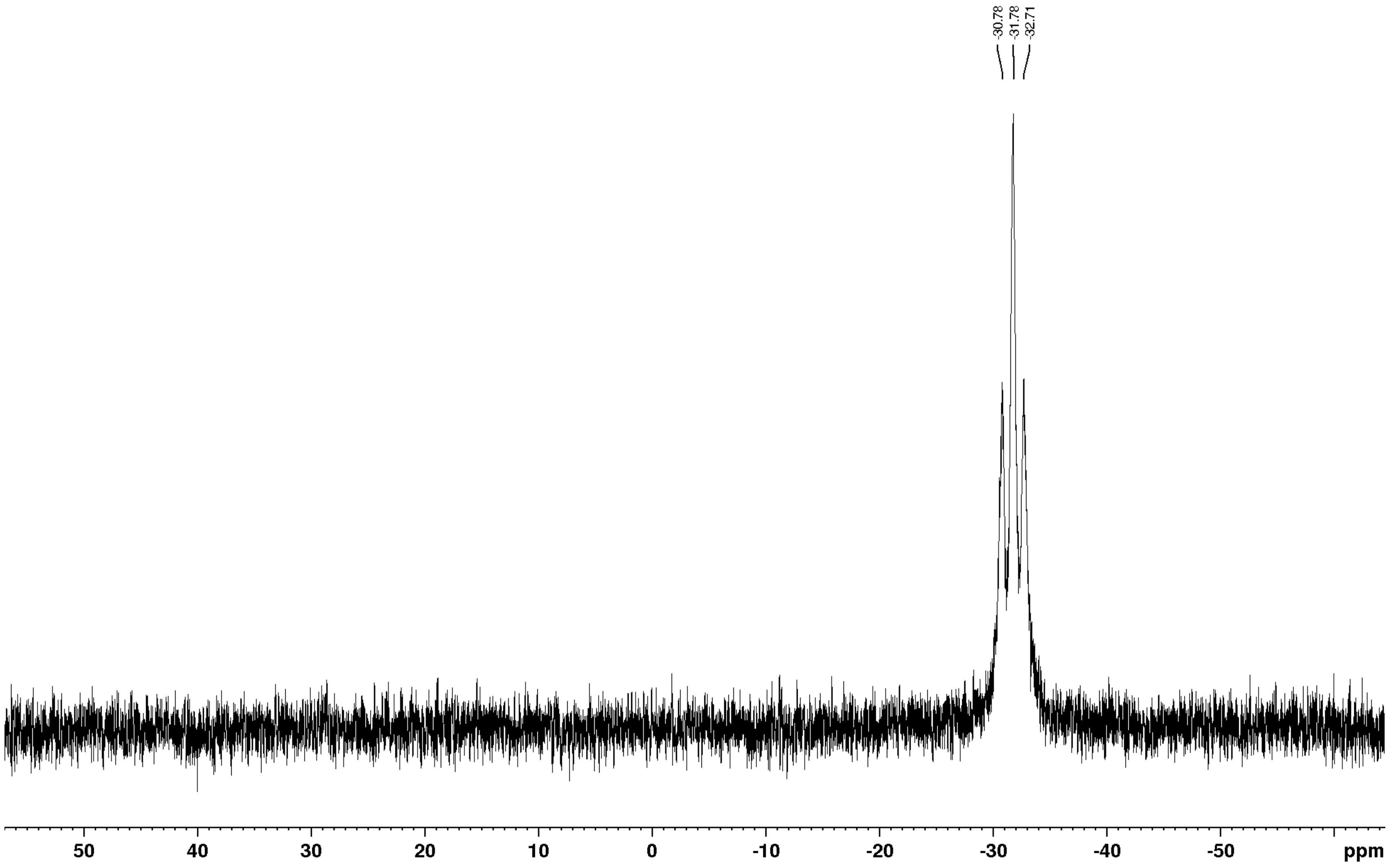

Figure S9. ^{11}B NMR spectrum (161 MHz, C_6D_6, 298 K) of IMe·BH_2I.

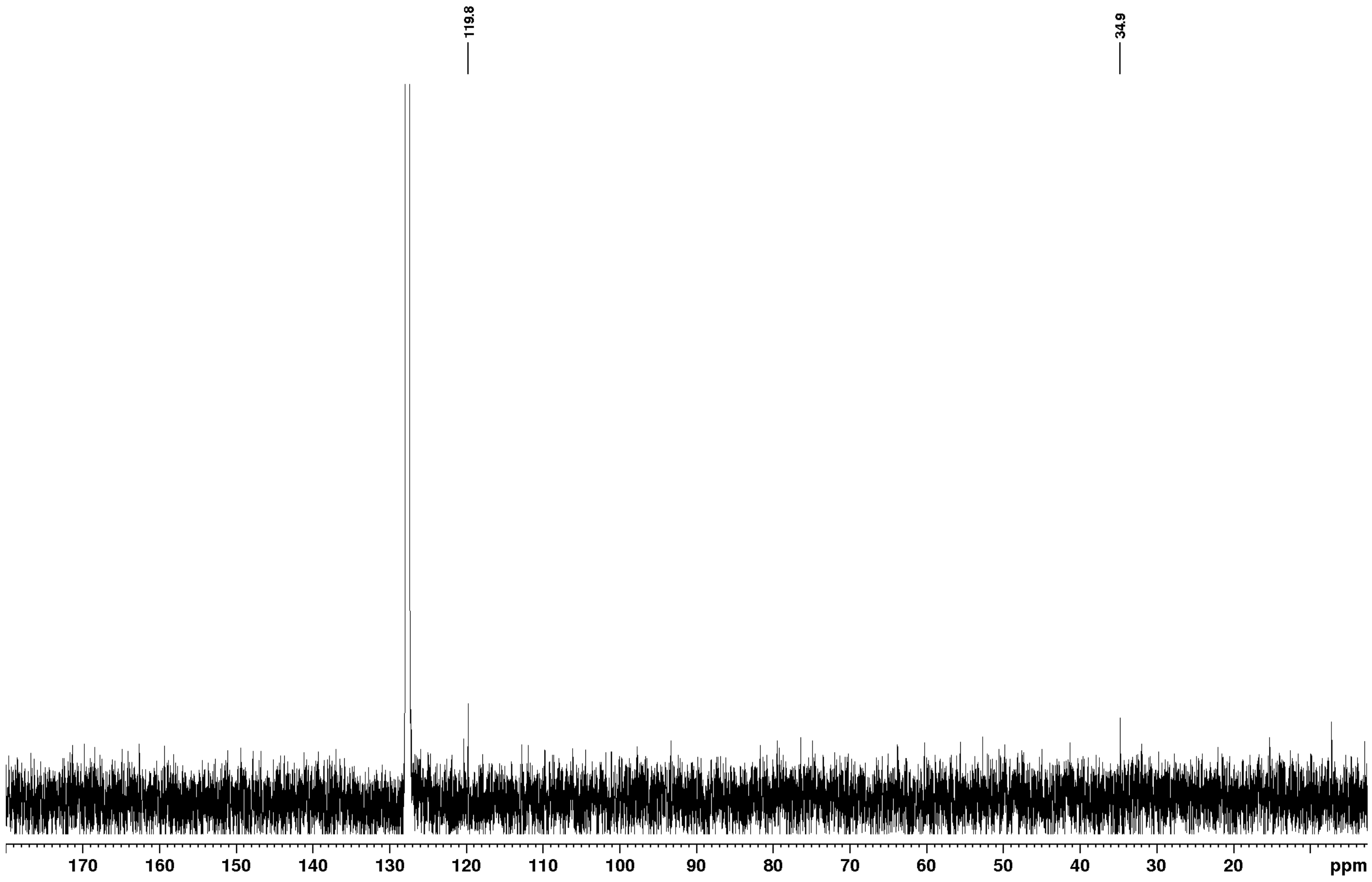

Figure S10. $^{13}C\{^1H\}$ NMR spectrum (126 MHz, C_6D_6, 298 K) of IMe·BH_2I.

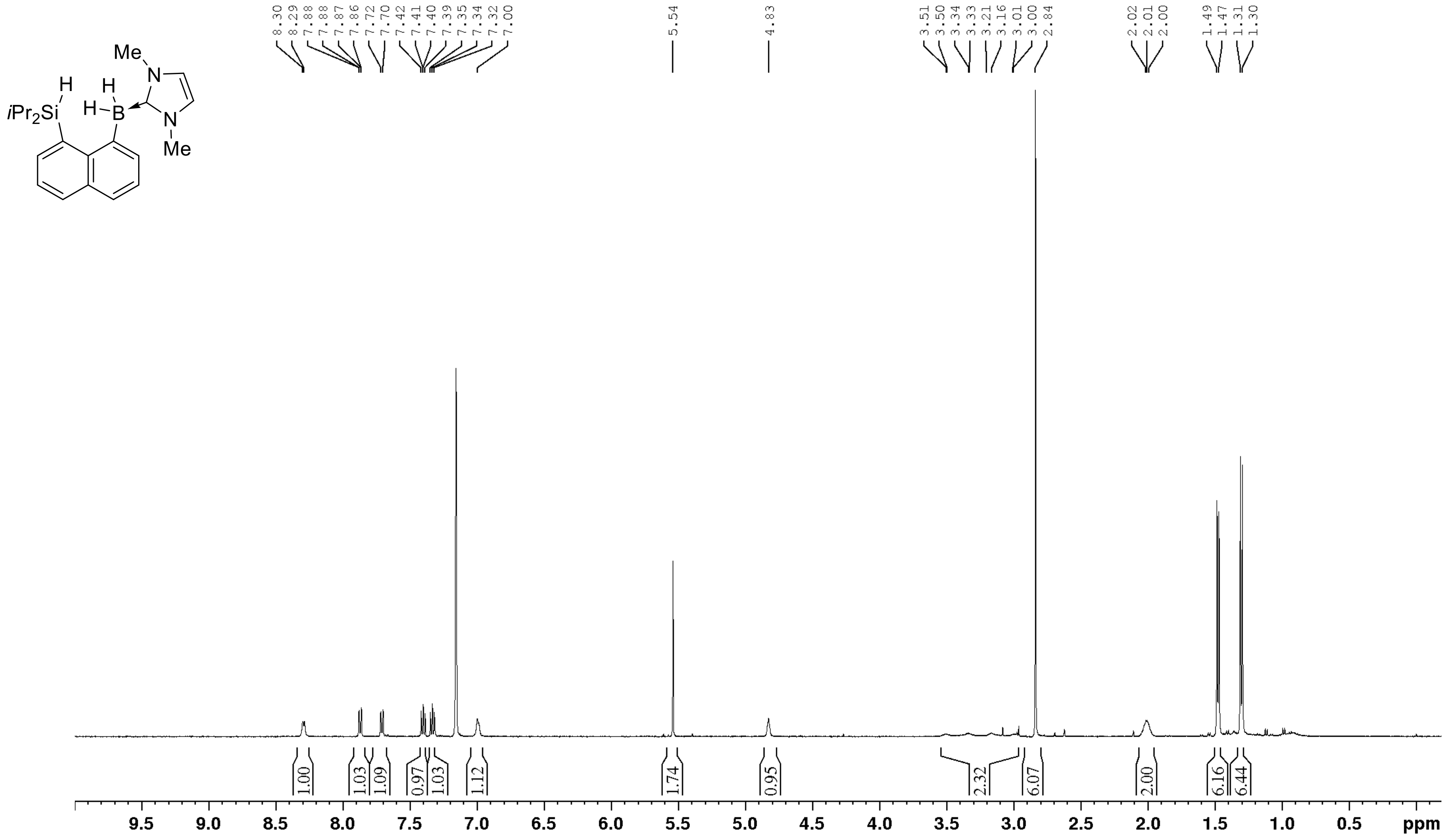

Figure S11. ^{1}H NMR spectrum (500 MHz, C_6D_6, 298 K) of precursor **9**.

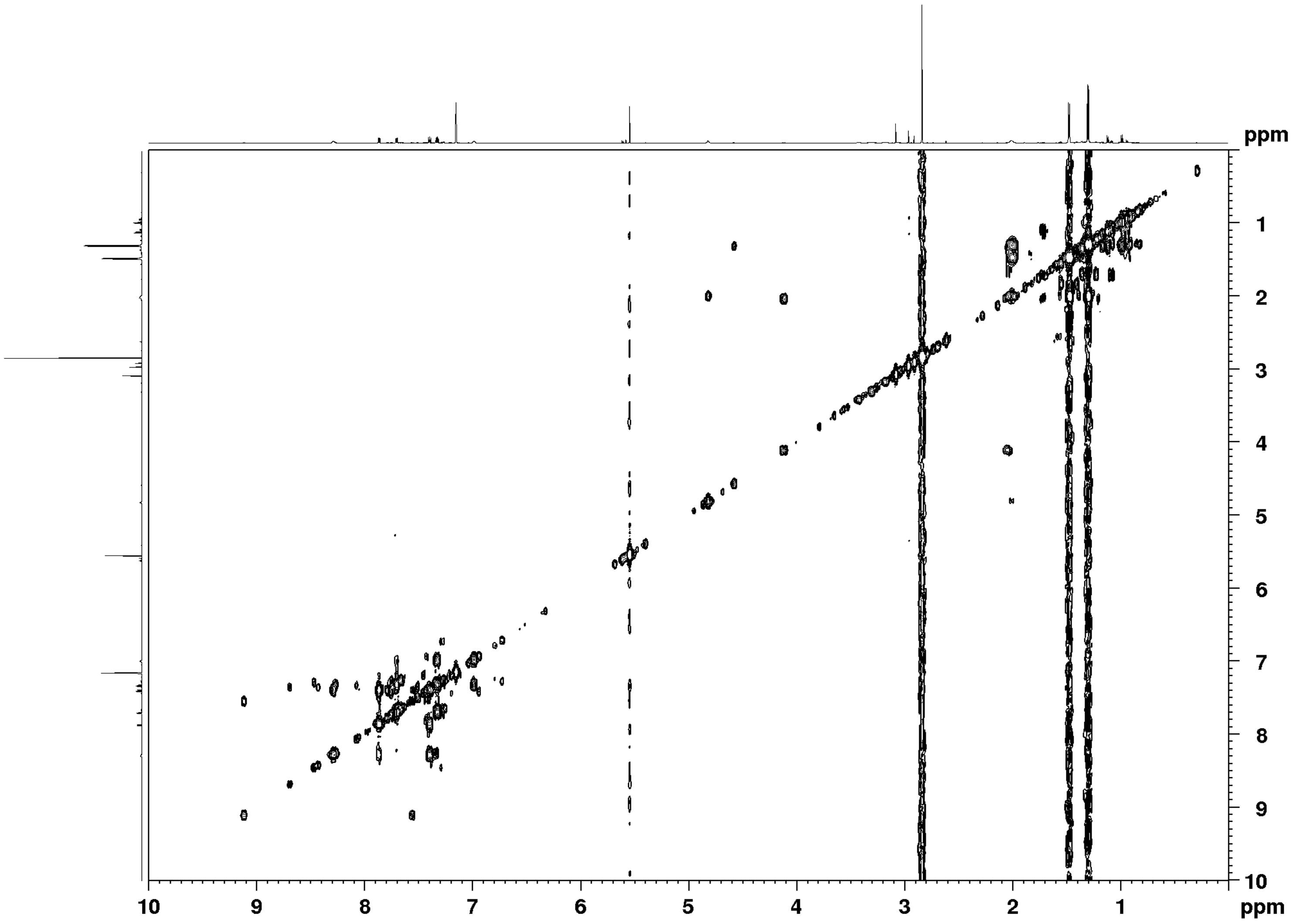

Figure S12. $^{1}H/^{1}H$ COSY NMR (700/700 MHz, C_6D_6, 298 K) of precursor **9**.

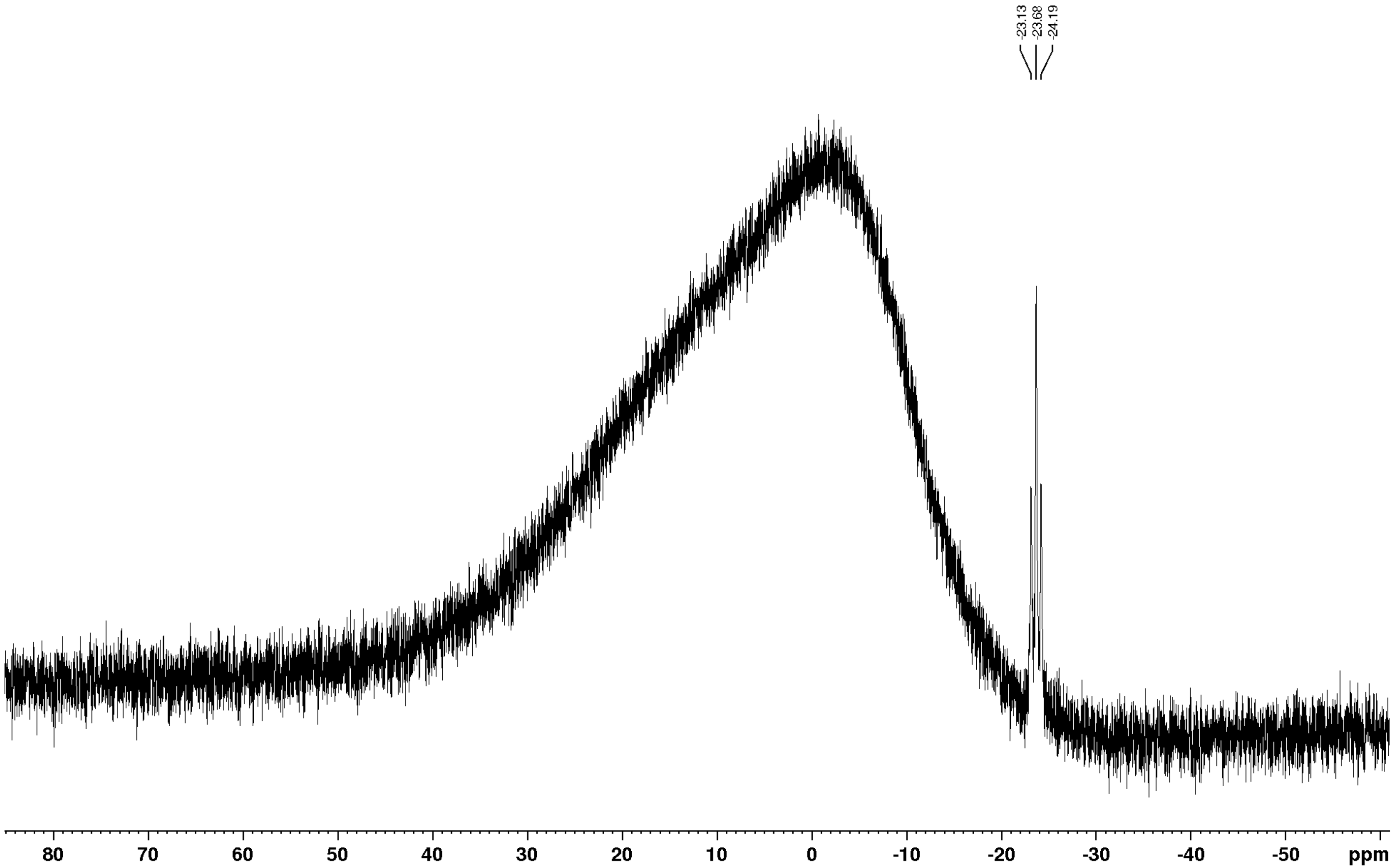

Figure S13. ^{11}B NMR spectrum (161 MHz, C_6D_6, 298 K) of precursor **9**.

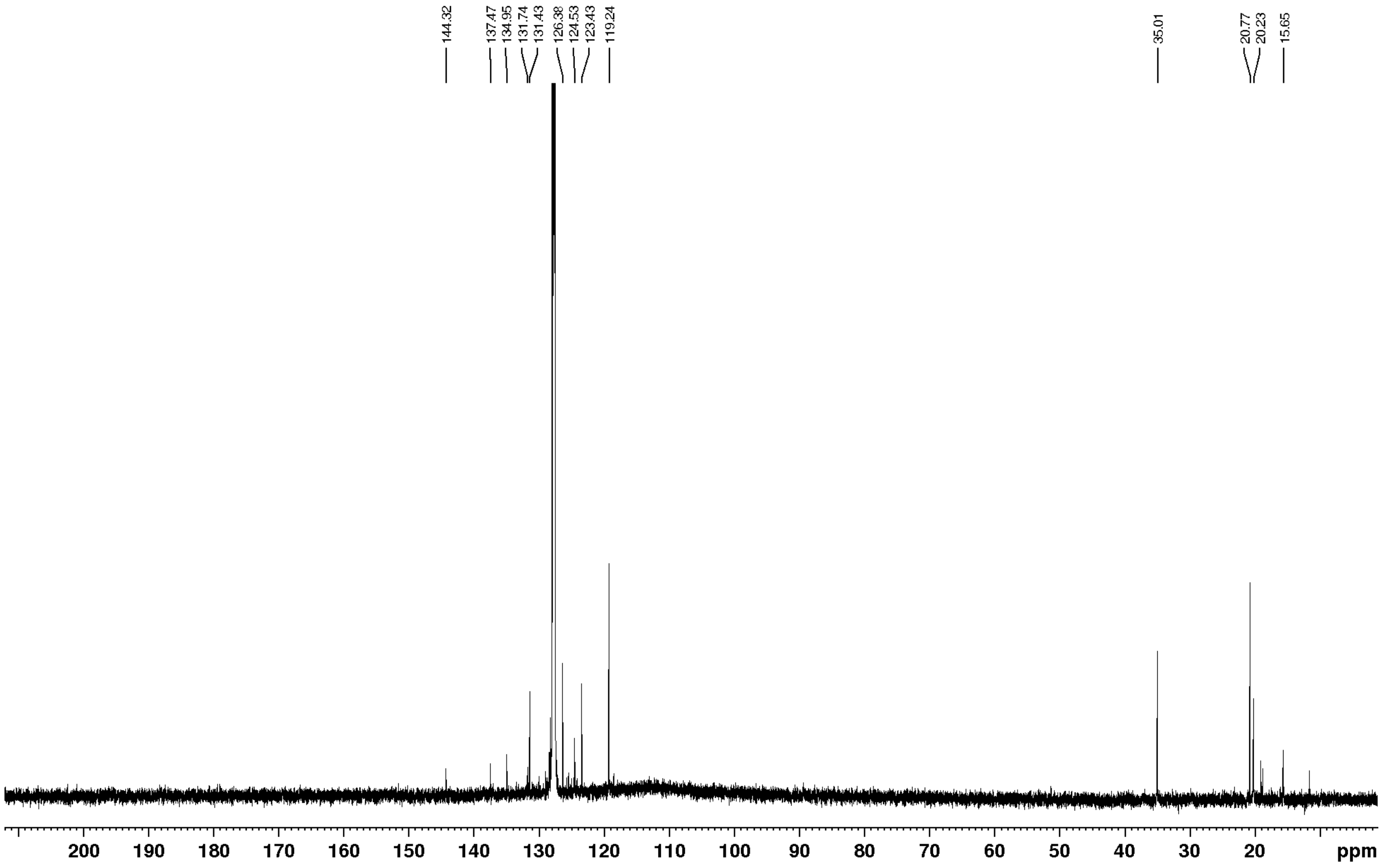

Figure S14. $^{13}C\{^1H\}$ NMR (175 MHz, C_6D_6, 298 K) of precursor **9**.

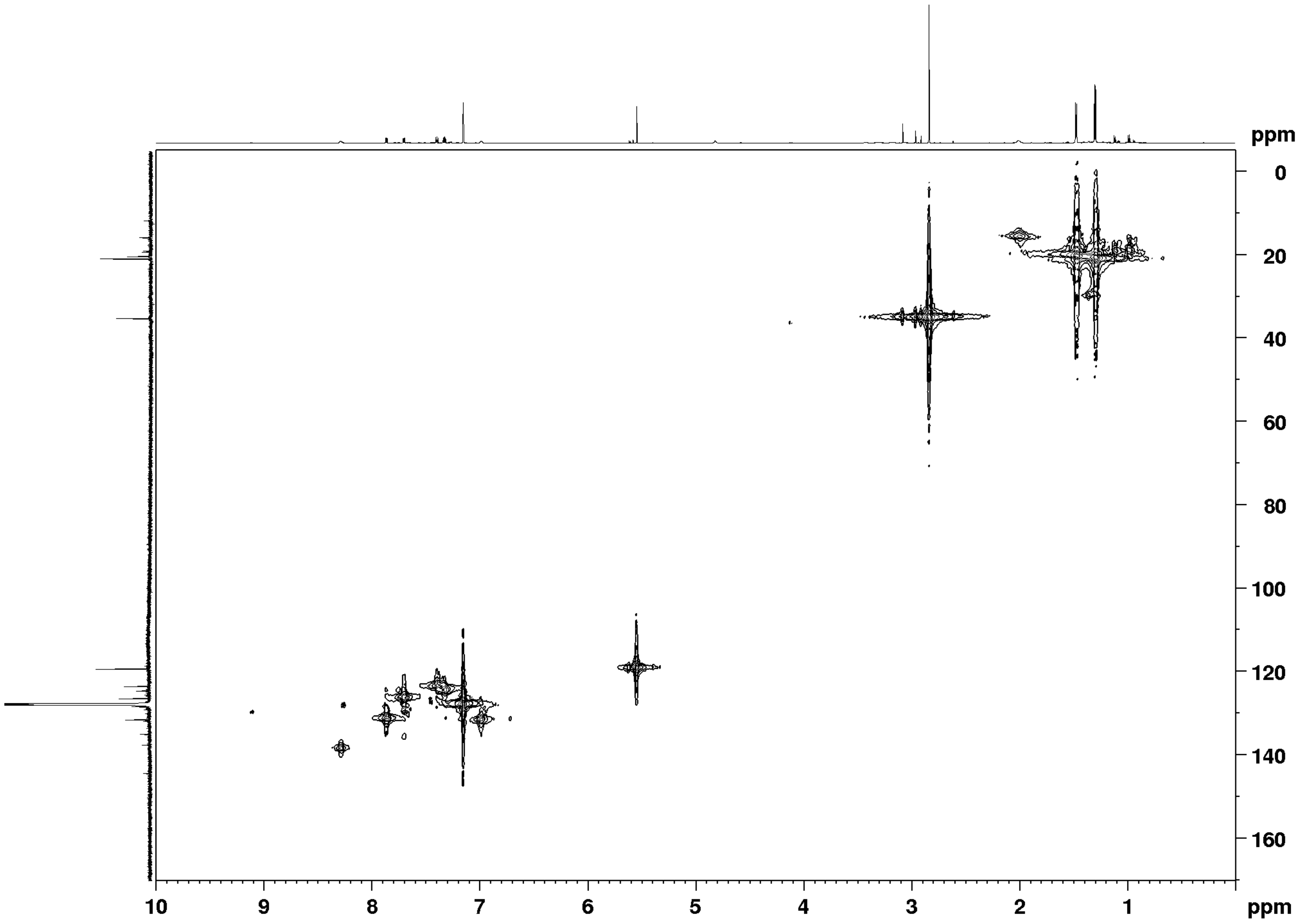

Figure S15. $^1H/^{13}C$ HSQC NMR (700/175 MHz, C_6D_6, 298 K) of precursor **9**.

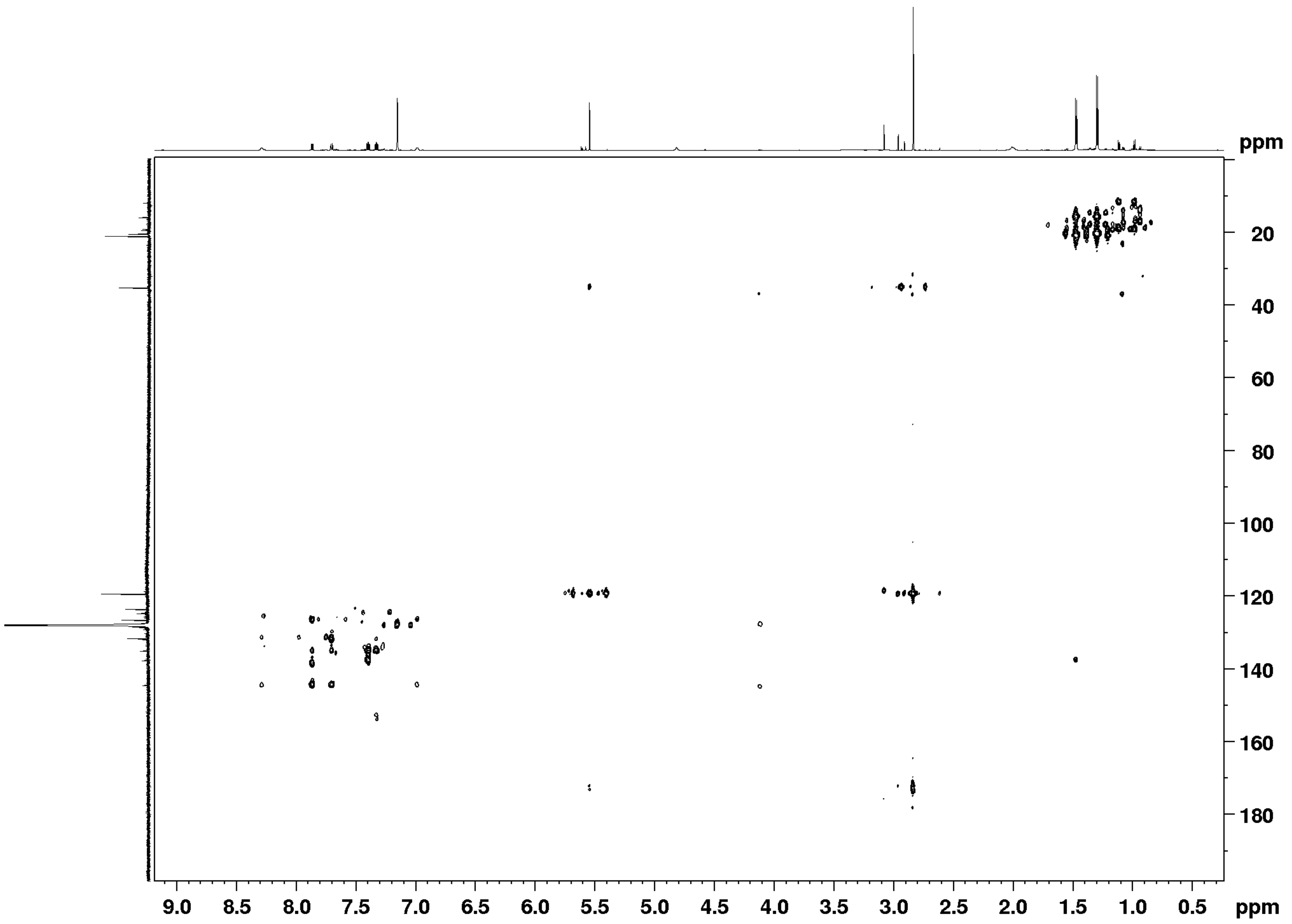

Figure S16. $^{1}H/^{13}C$ HMBC NMR (700/175 MHz, C_6D_6, 298 K) of precursor **9**.

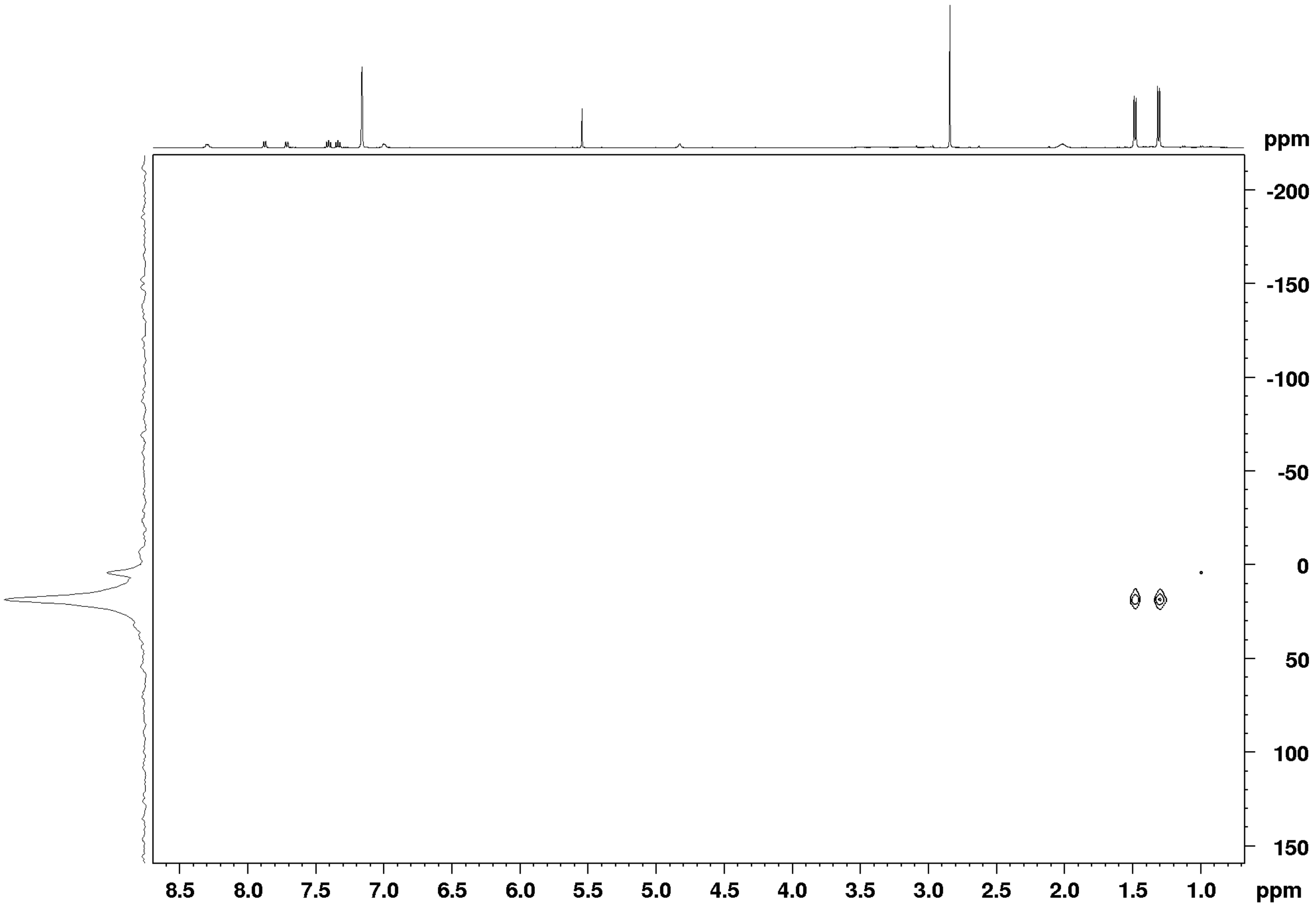

Figure S17. $^{1}H/^{29}Si$ HMQC NMR (500/99 MHz, C_6D_6, 298 K, optimized for *J* = 7 Hz) of precursor **9**.

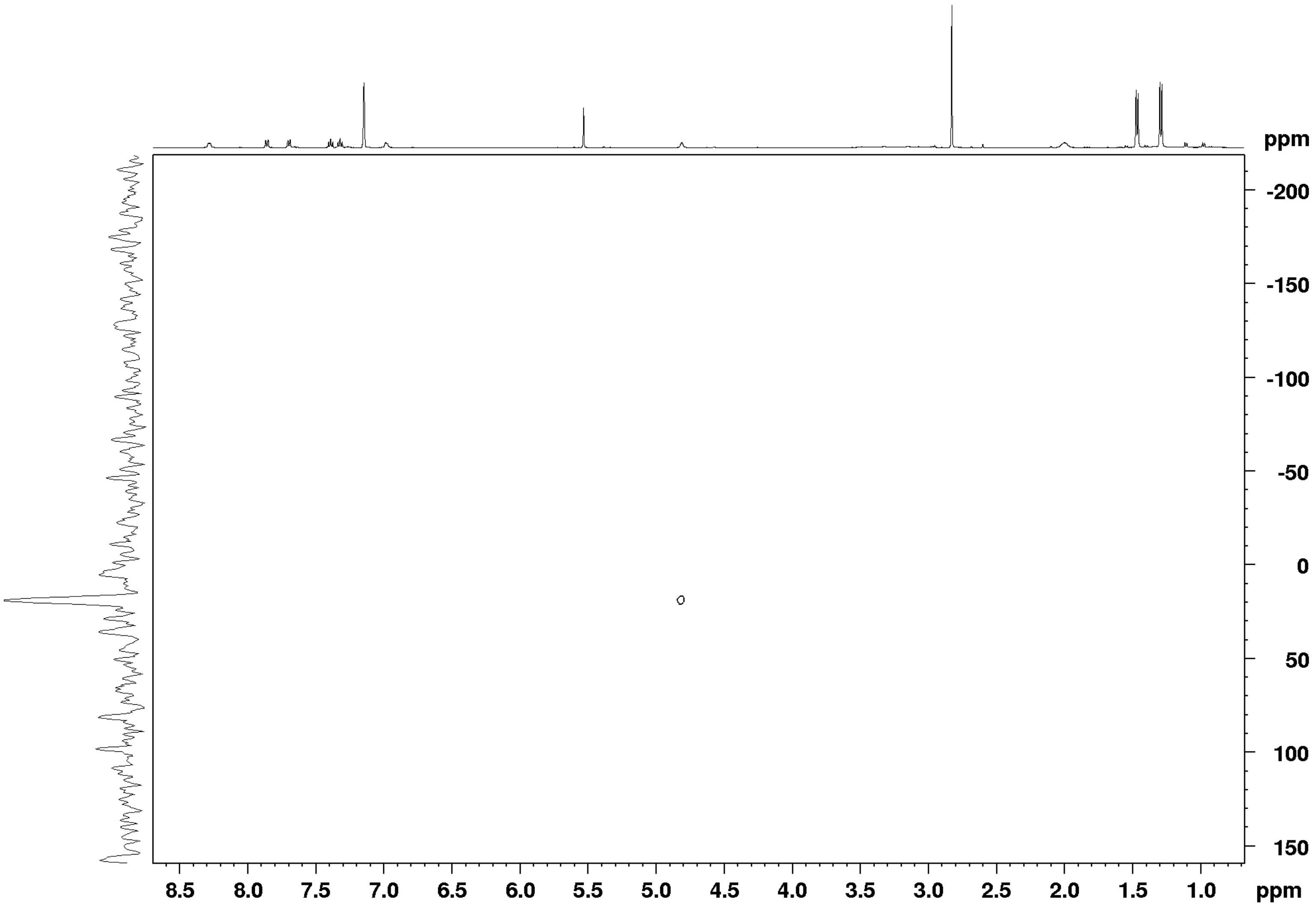

Figure S18. $^{1}H/^{29}Si$ HMQC NMR (500/99 MHz, C_6D_6, 298 K, optimized for J = 200 Hz) of precursor **9**.

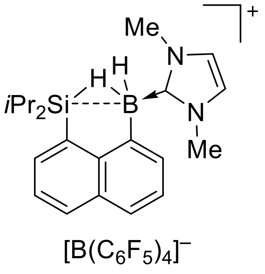

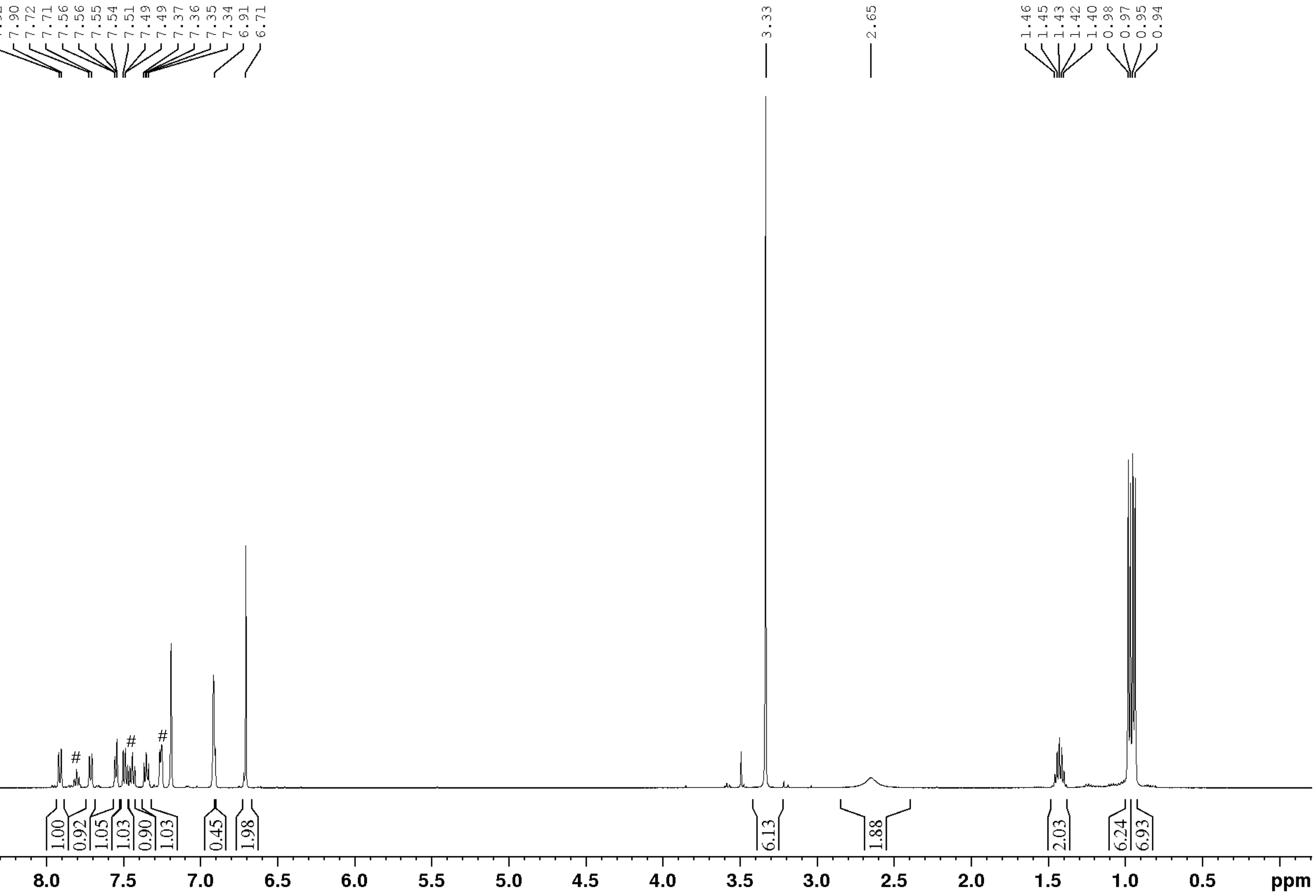

Figure S19. ^{1}H NMR spectrum (500 MHz, 1,2-$Cl_2C_6D_4$, 298 K) of Si/B hydronium borate **10**$^{+}[B(C_6F_5)_4]^{-}$ (# = trityl salt).

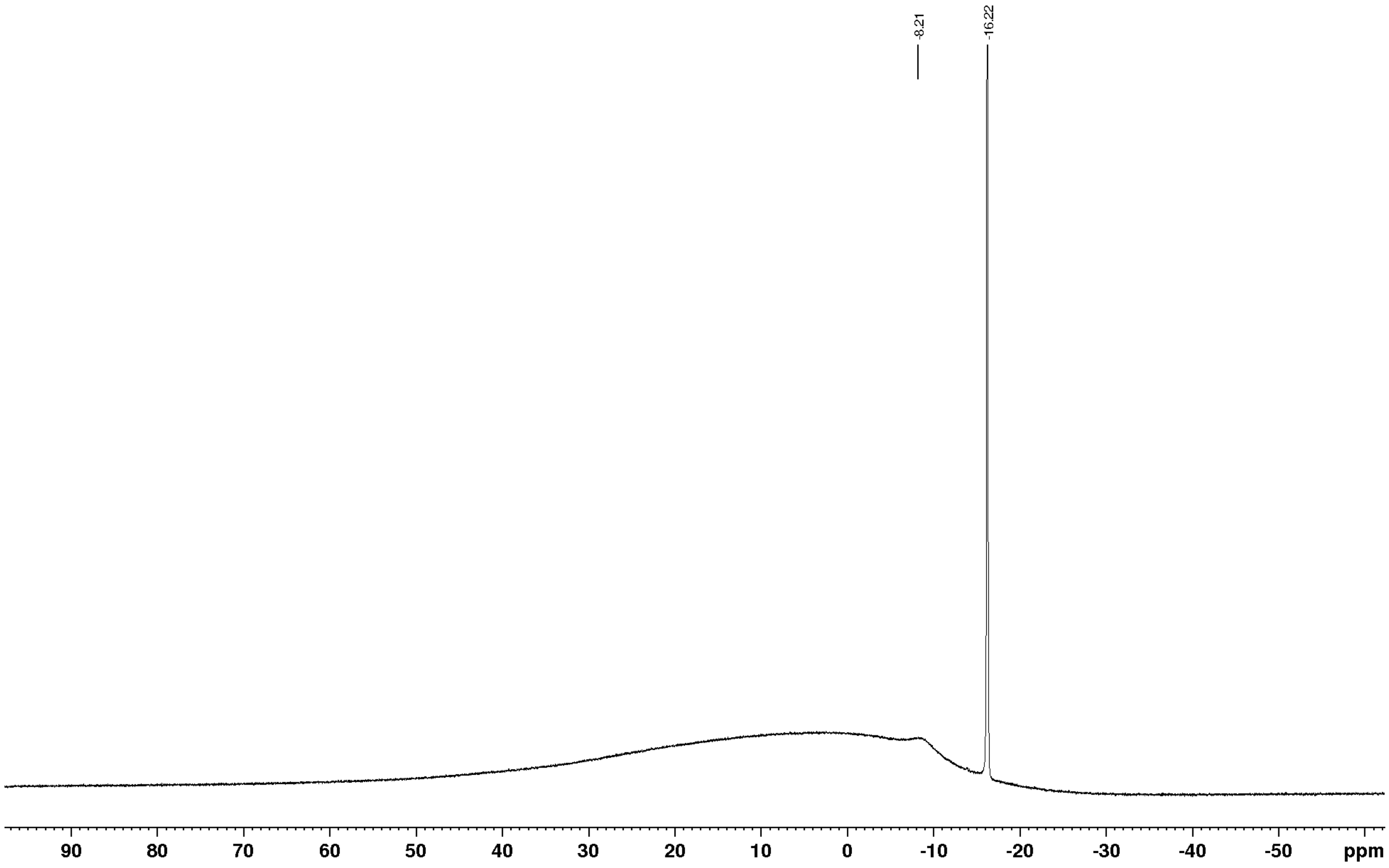

Figure S20. ^{11}B NMR spectrum (161 MHz, 1,2-$Cl_2C_6D_4$, 298 K) of Si/B hydronium borate **10**$^{+}$[B(C_6F_5)$_4$]$^{-}$.

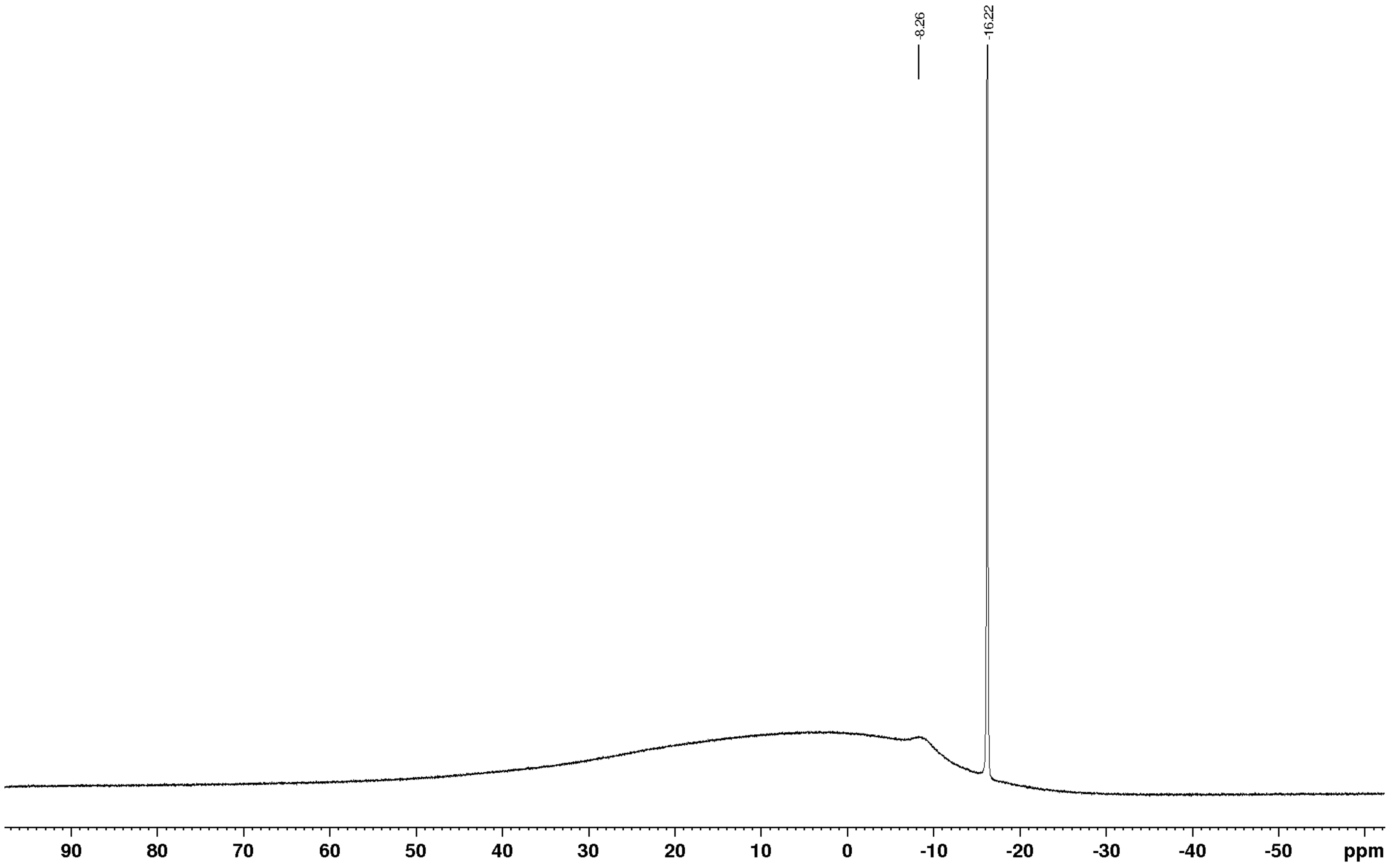

Figure S21. $^{11}B\{^1H\}$ NMR spectrum (161 MHz, 1,2-$Cl_2C_6D_4$, 298 K) of Si/B hydronium borate **10**$^+[B(C_6F_5)_4]^-$.

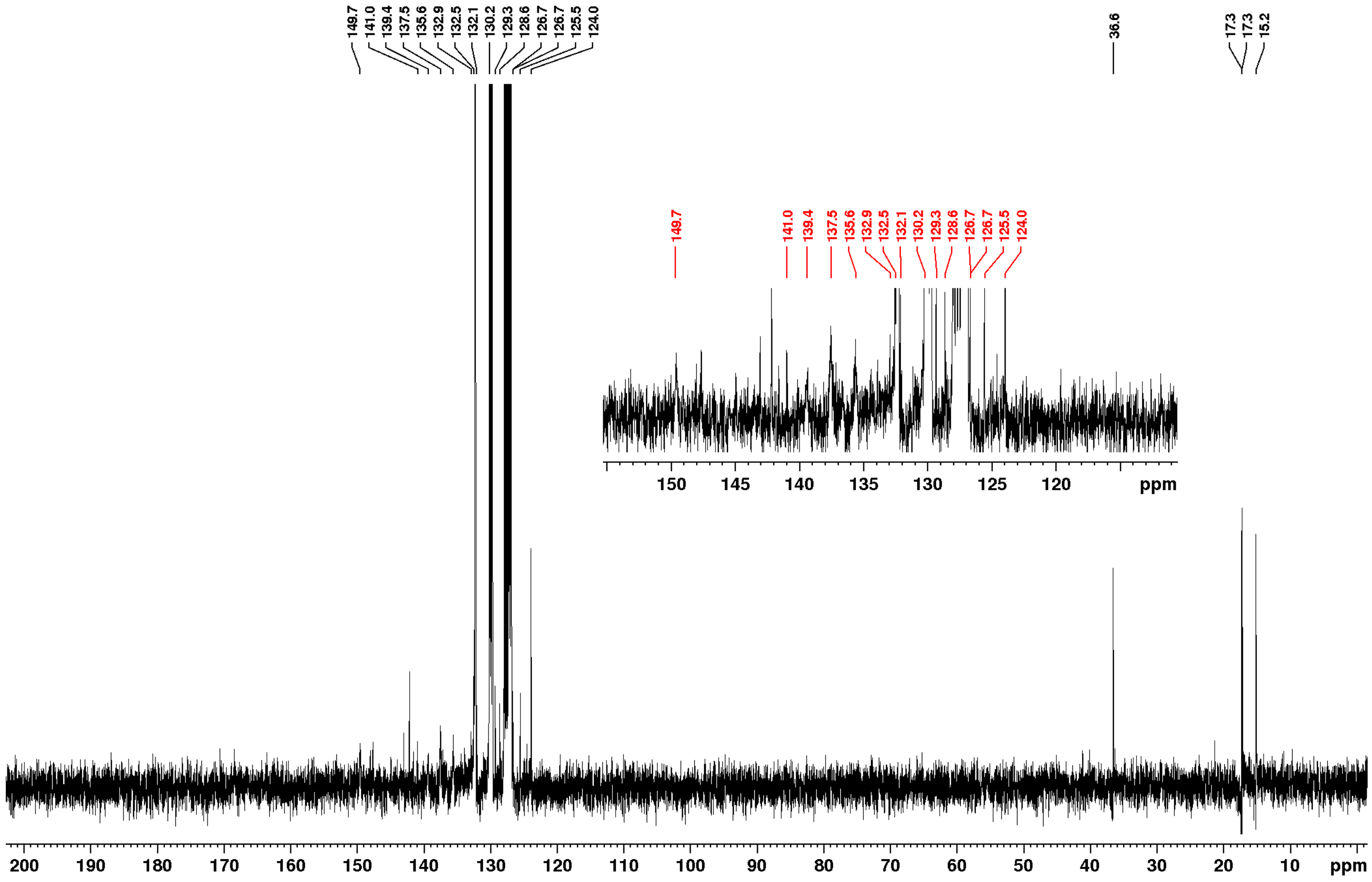

Figure S22. $^{13}C\{^{1}H\}$ NMR (126 MHz, 1,2-$Cl_2C_6D_4$, 298 K) of Si/B hydronium borate **10**$^{+}$[B(C_6F_5)$_4$]$^{-}$.

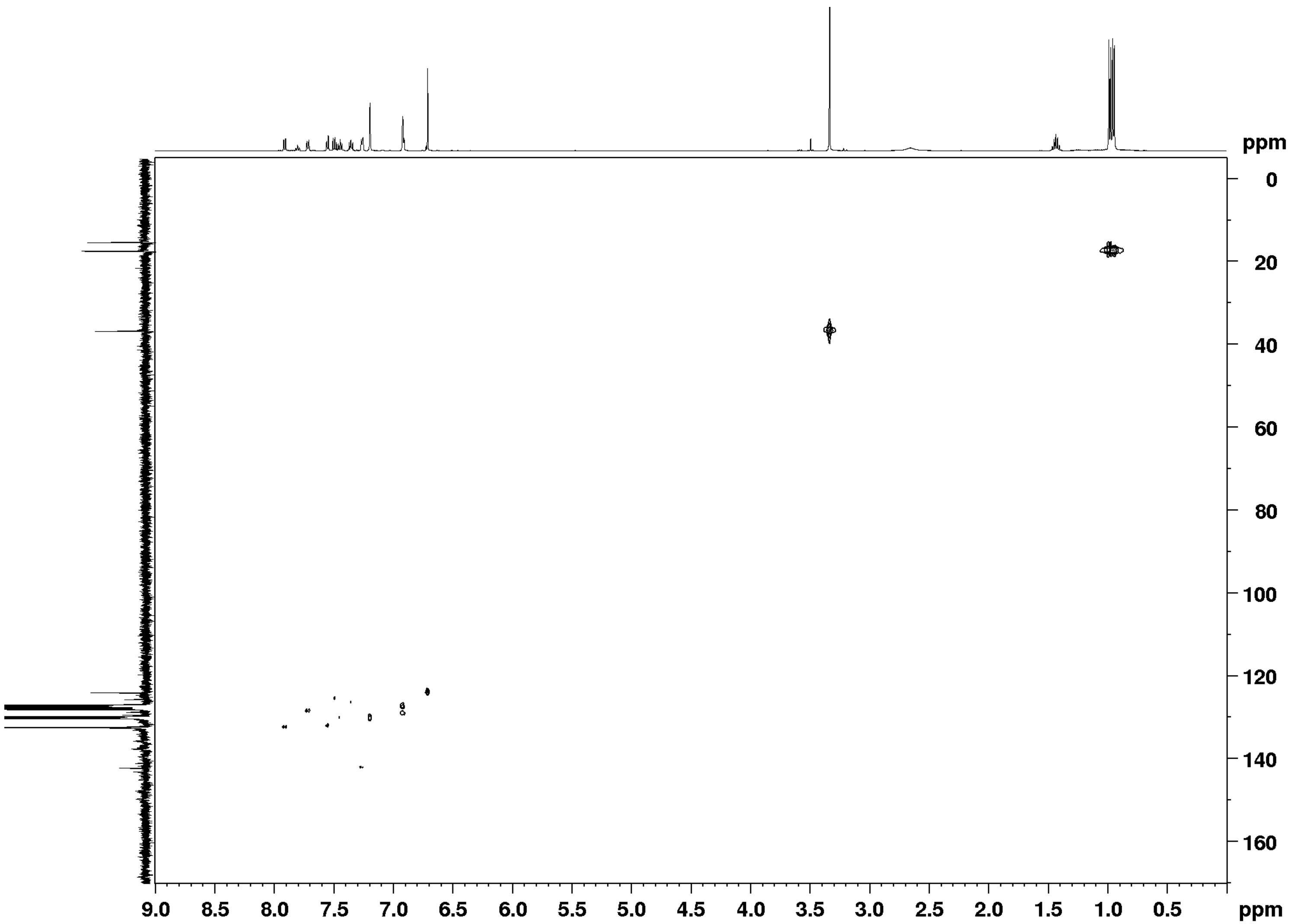

Figure S23. $^{1}H/^{13}C$ HSQC NMR (500/126 MHz, 1,2-$Cl_2C_6D_4$, 298 K) of Si/B hydronium borate **10**$^{+}[B(C_6F_5)_4]^{-}$.

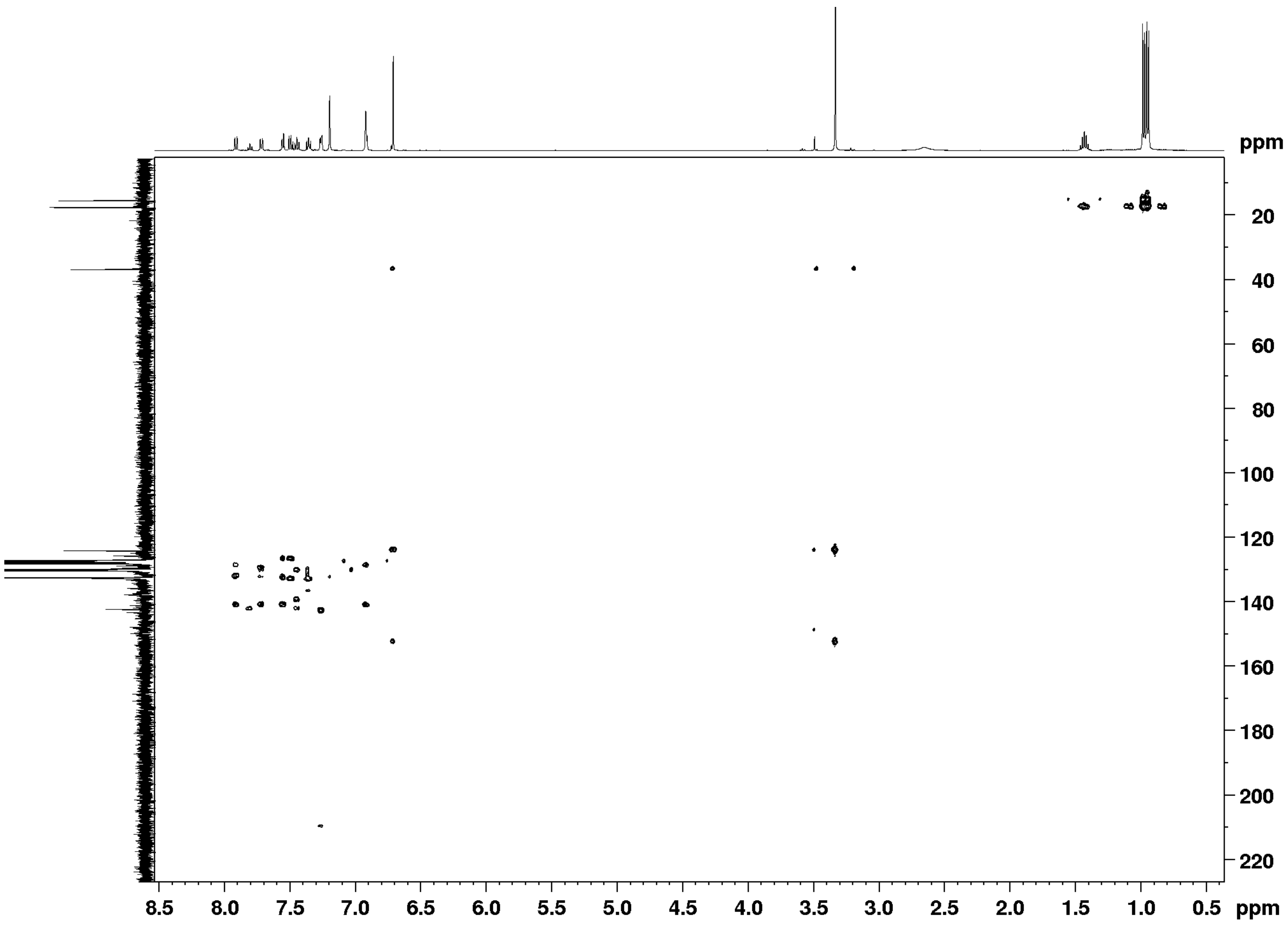

Figure S24. $^{1}H/^{13}C$ HMBC NMR (500/126 MHz, 1,2-$Cl_2C_6D_4$, 298 K) of Si/B hydronium borate **10**$^{+}[B(C_6F_5)_4]^{-}$.

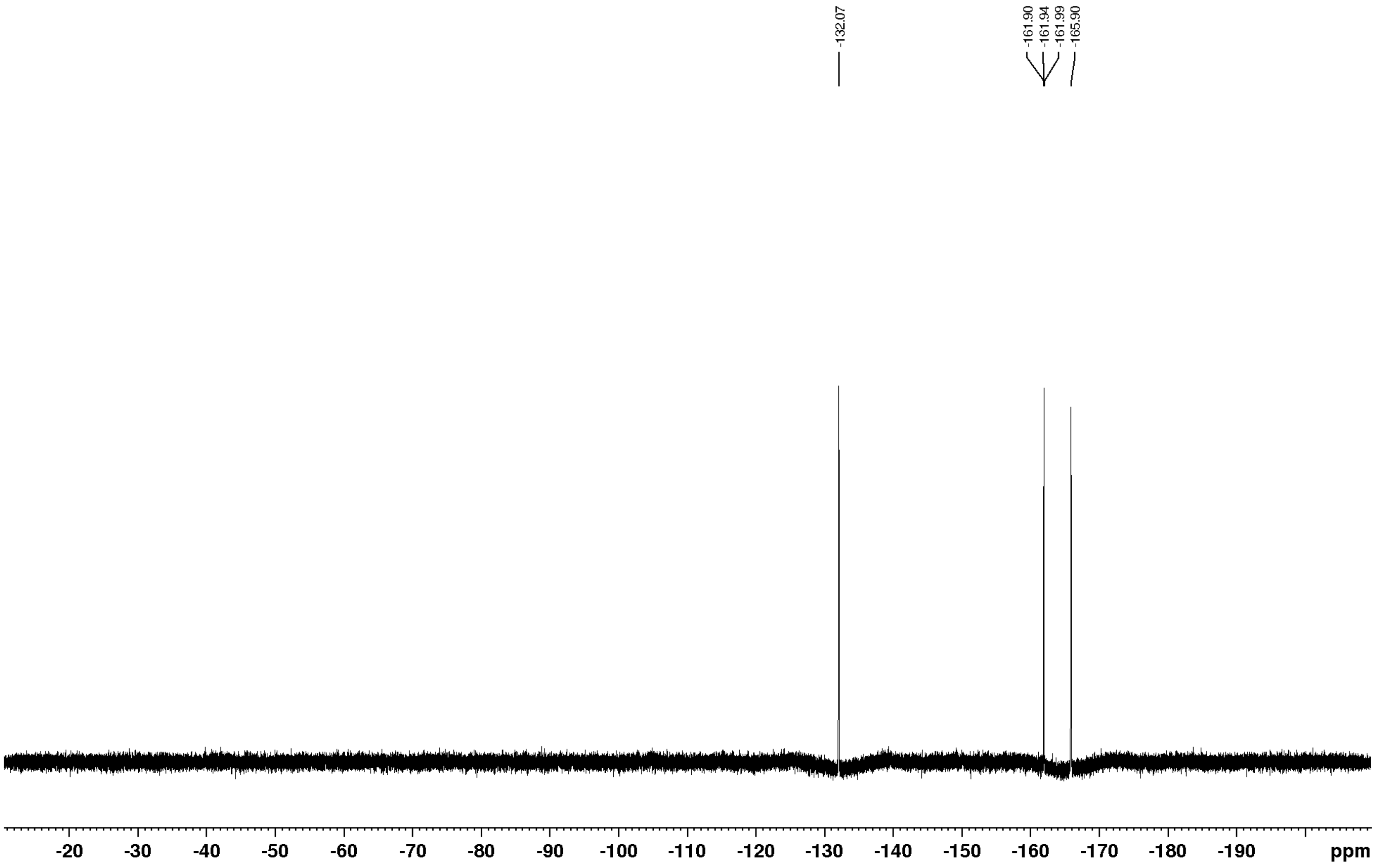

Figure S25. ^{19}F NMR spectrum (471 MHz, 1,2-$Cl_2C_6D_4$, 298 K) of Si/B hydronium borate **10**$^{+}$[B(C_6F_5)$_4$]$^{-}$.

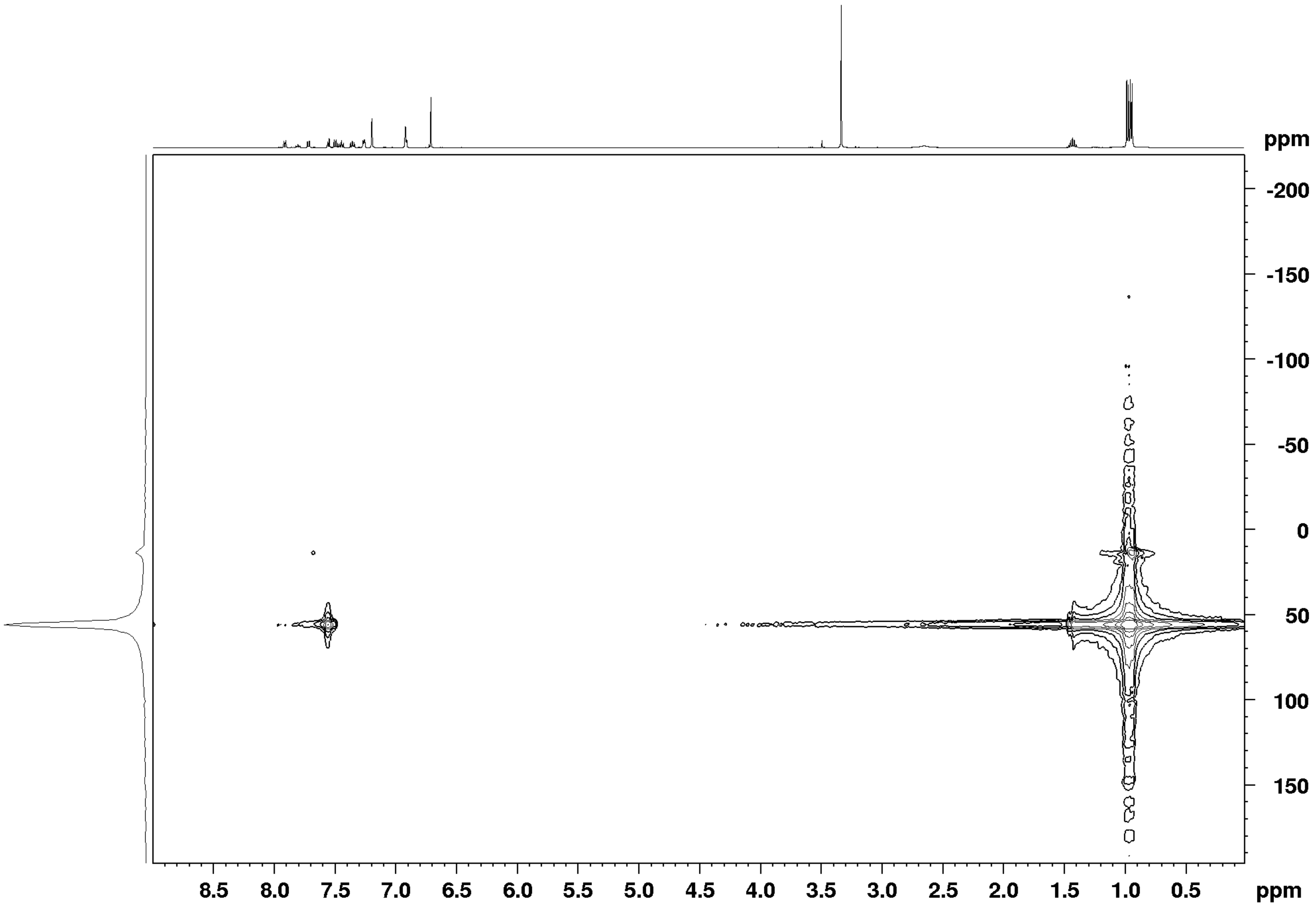

Figure S26. $^{1}H/^{29}Si$ HMQC NMR (500/99 MHz, 1,2-$Cl_2C_6D_4$, 298 K, optimized for *J* = 7 Hz) of Si/B hydronium borate **10**$^{+}$[$B(C_6F_5)_4$]$^{-}$.

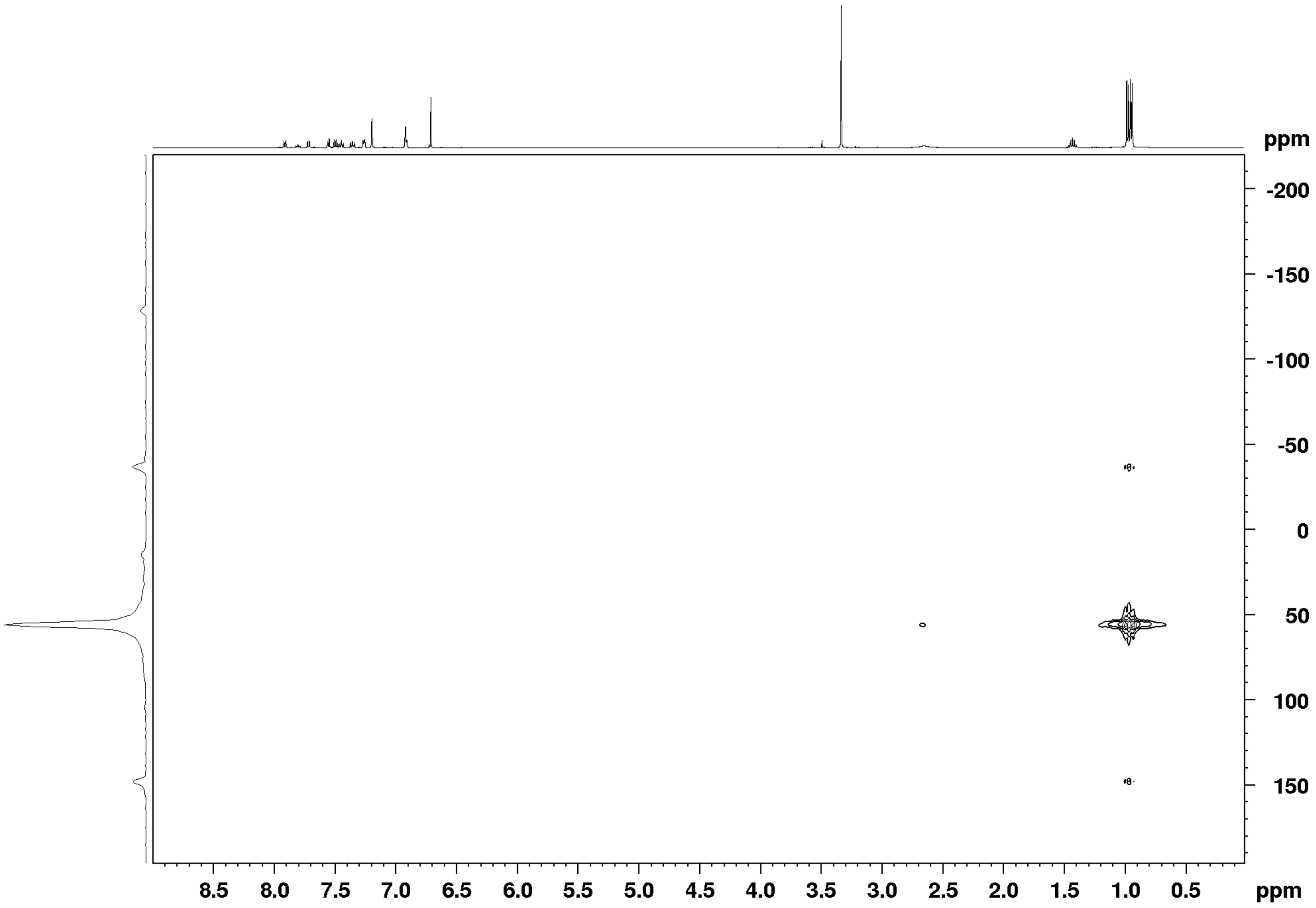

Figure S27. $^{1}H/^{29}Si$ HMQC NMR (500/99 MHz, 1,2-$Cl_2C_6D_4$, 298 K, optimized for *J* = 45 Hz) of Si/B hydronium borate **10**$^{+}[B(C_6F_5)_4]^{-}$.

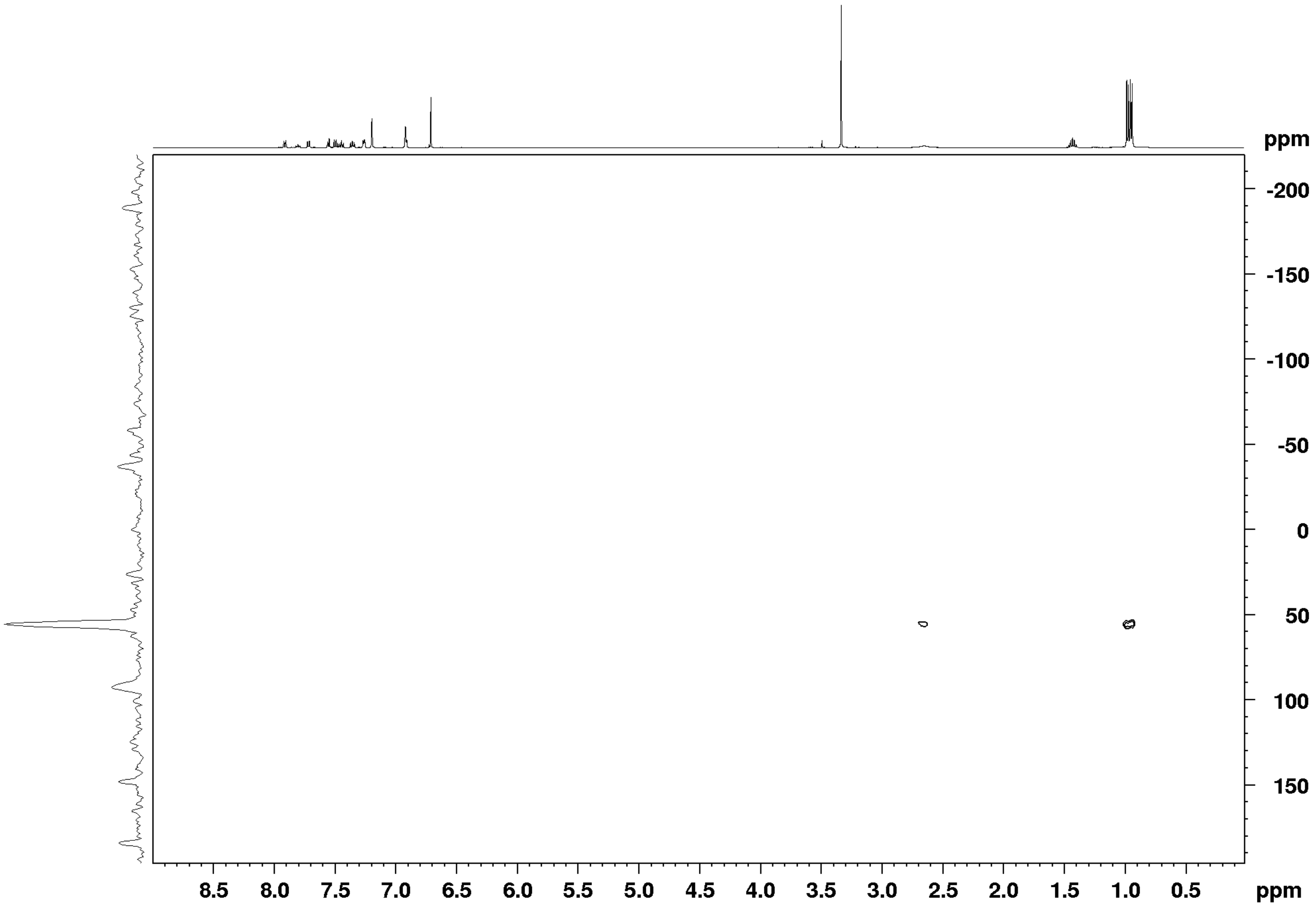

Figure S28. $^{1}H/^{29}Si$ HMQC NMR (500/99 MHz, 1,2-$Cl_2C_6D_4$, 298 K, optimized for J = 200 Hz) of Si/B hydronium borate **10**$^{+}[B(C_6F_5)_4]^{-}$.

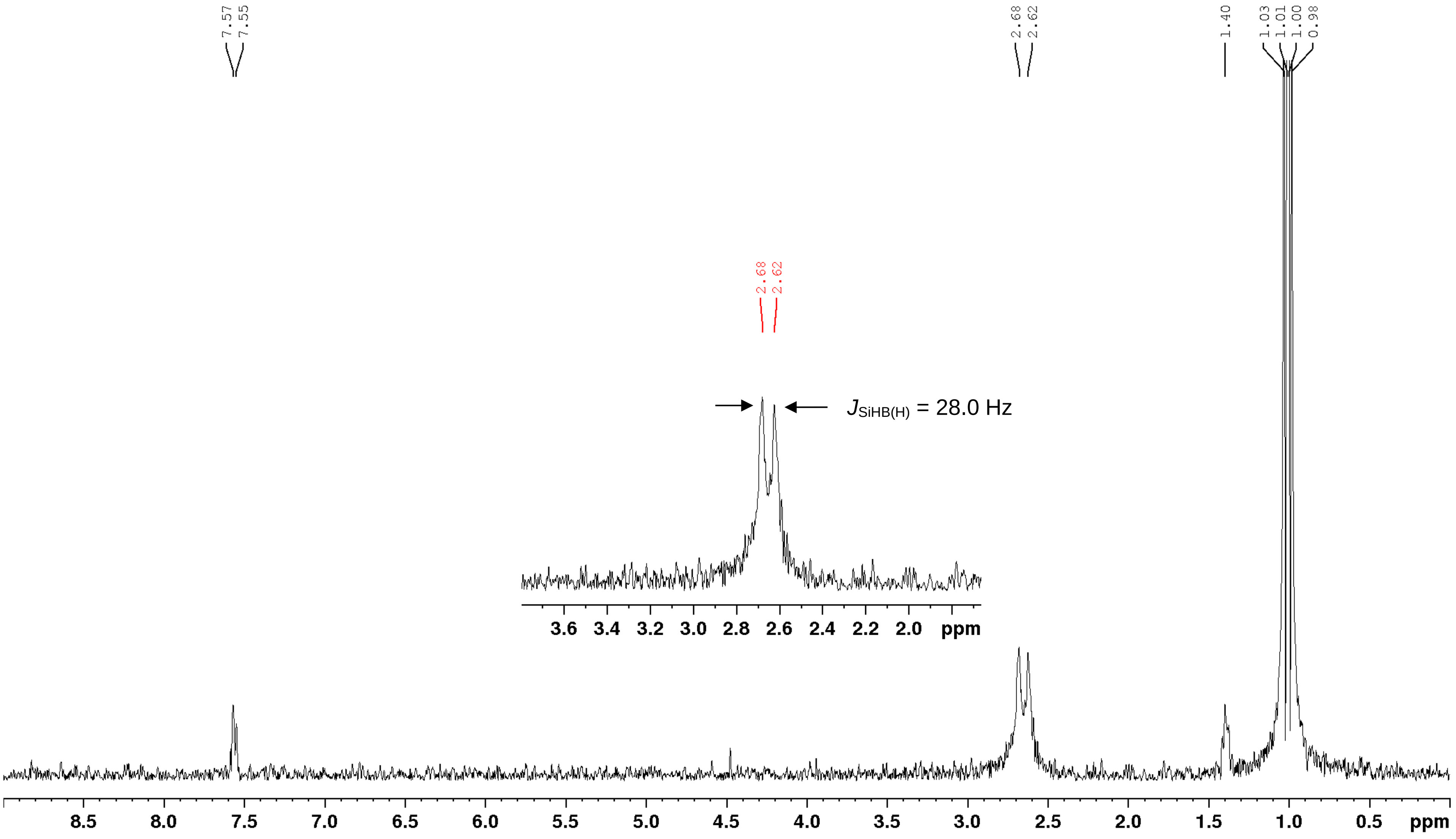

Figure S29. $^{1}H/^{29}Si$-1D-CLIP-HSQMBC NMR (500/99 MHz, ClC_6D_5, 240 K) of Si/B hydronium borate **10**$^{+}[B(C_6F_5)_4]^{-}$.

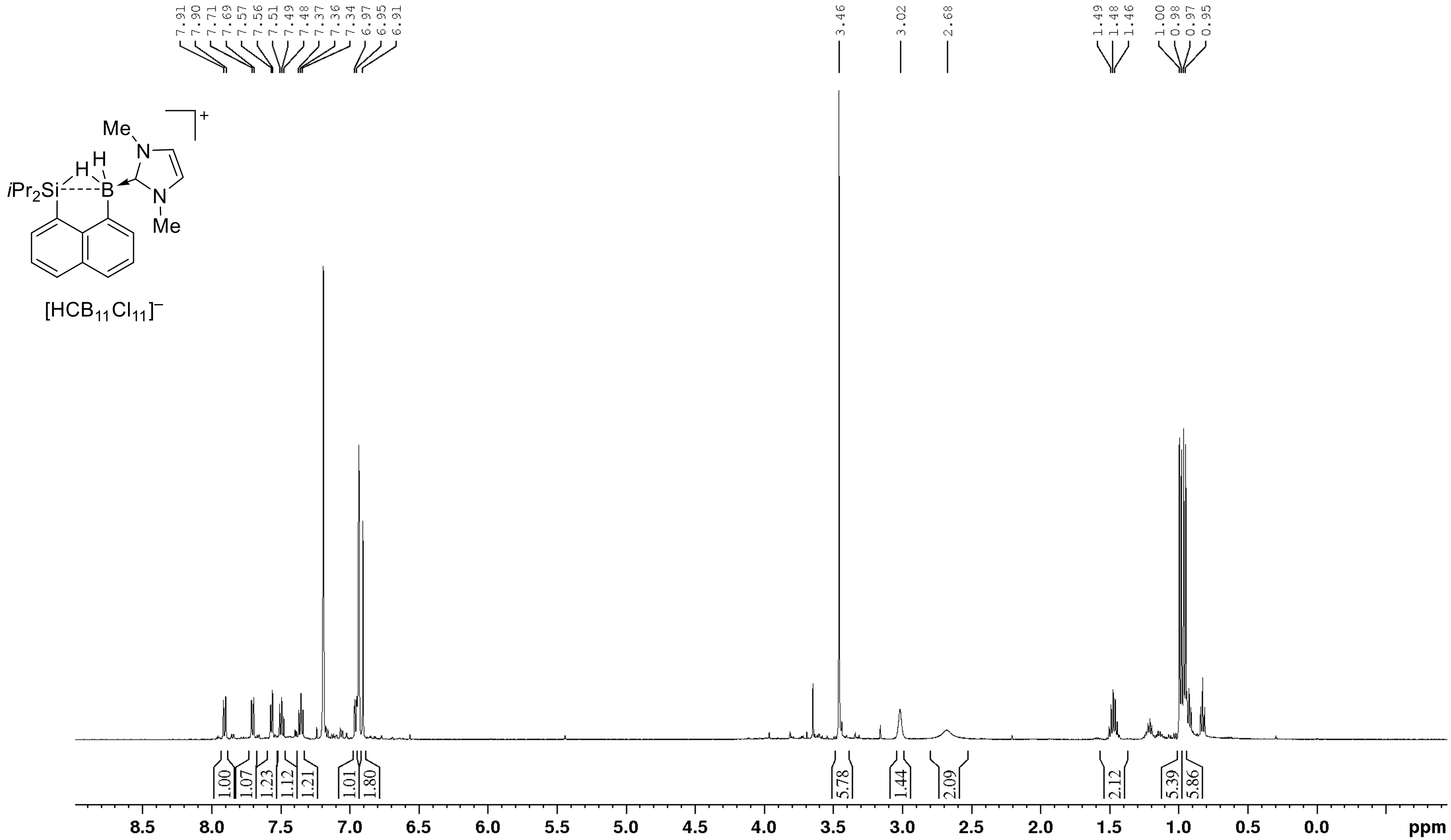

Figure S30. ^{1}H NMR spectrum (500 MHz, 1,2-$Cl_2C_6D_4$, 298 K) of Si/B hydronium carborate **10**$^+$[$HCB_{11}Cl_{11}$]$^-$.

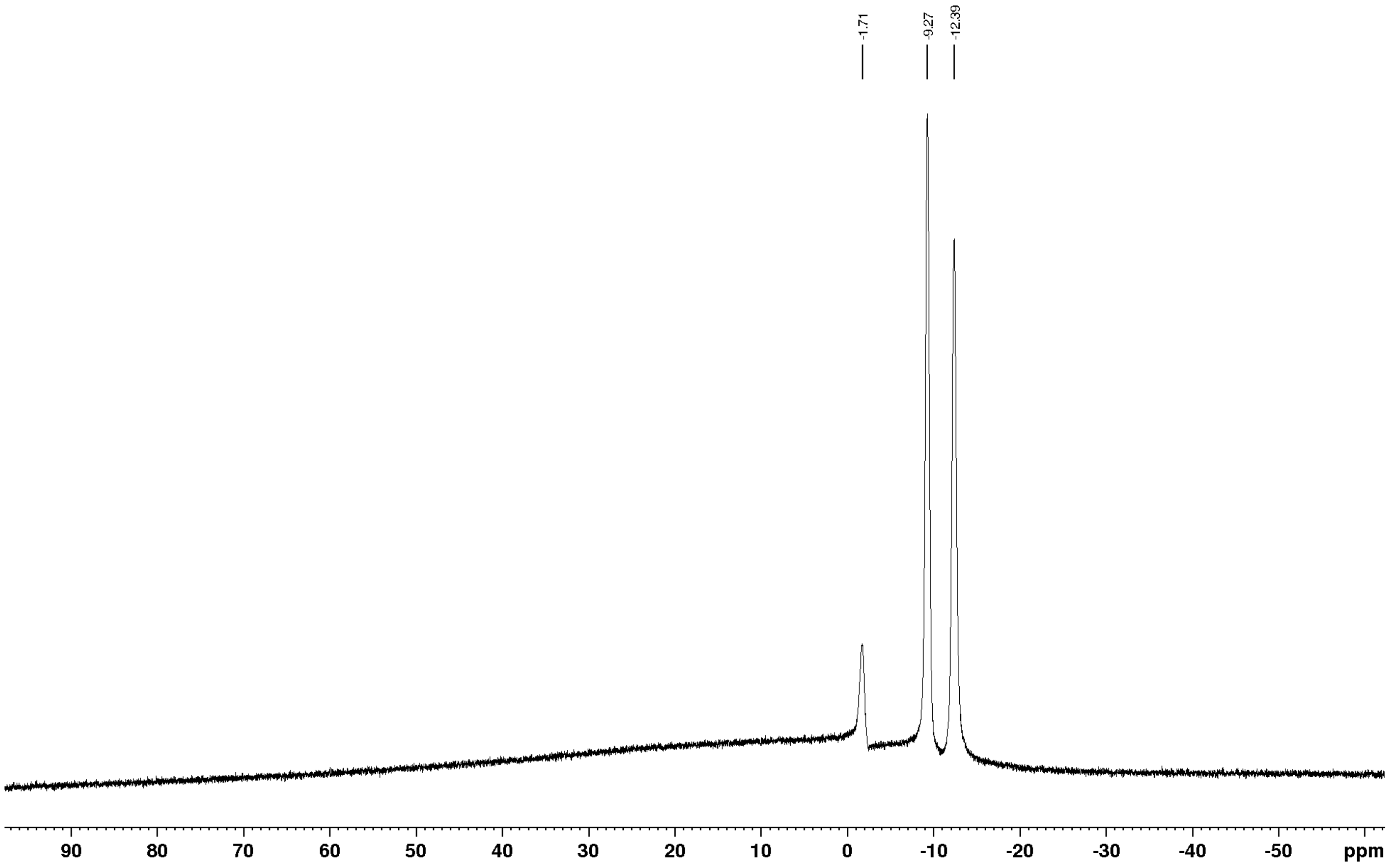

Figure S31. ^{11}B NMR spectrum (161 MHz, 1,2-$Cl_2C_6D_4$, 298 K) of Si/B hydronium carborate **10**$^+[HCB_{11}Cl_{11}]^-$.

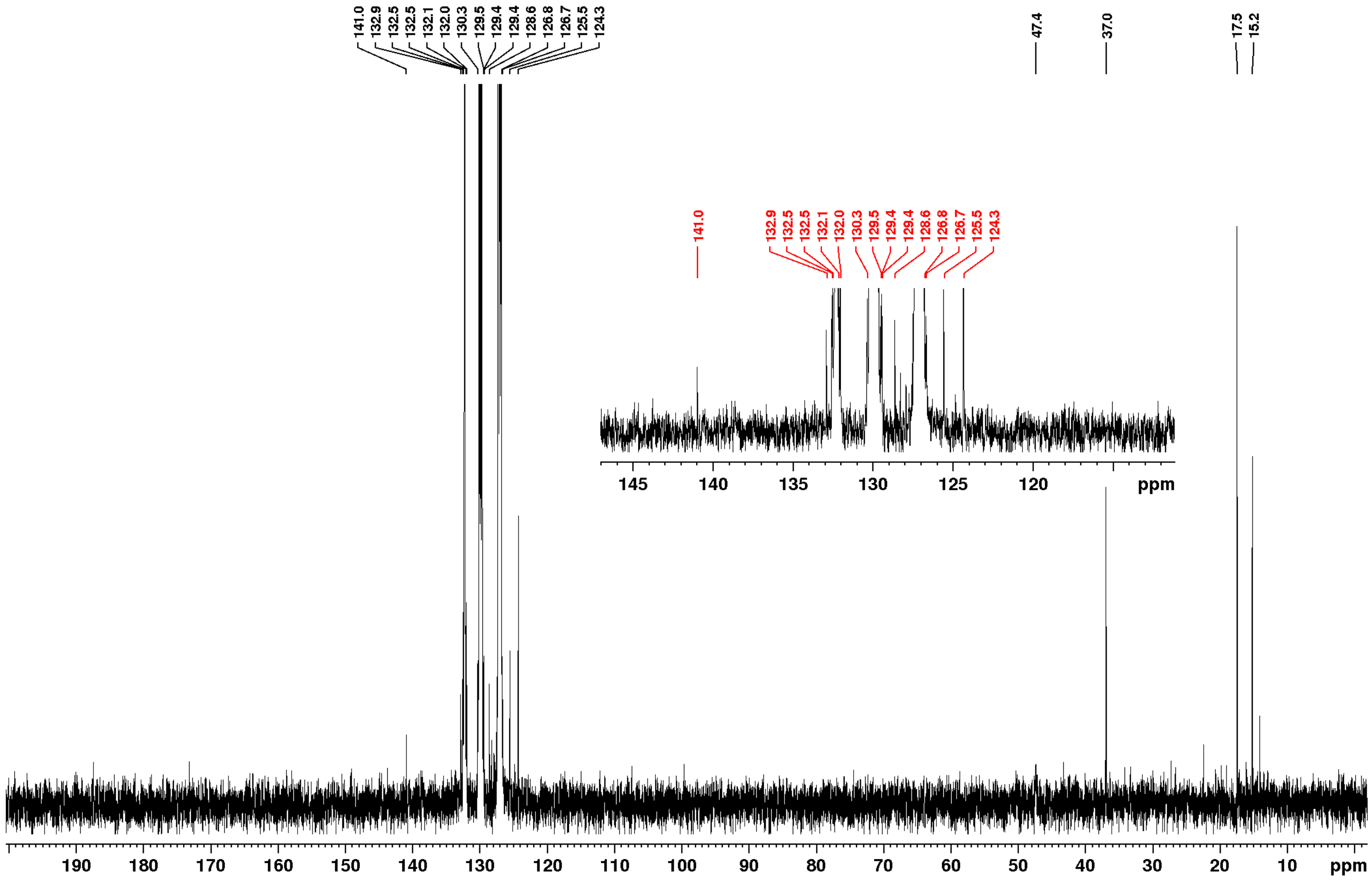

Figure S32. $^{13}C\{^{1}H\}$ NMR (126 MHz, 1,2-$Cl_2C_6D_4$, 298 K) of Si/B hydronium carborate **10**$^{+}$[$HCB_{11}Cl_{11}$]$^{-}$.

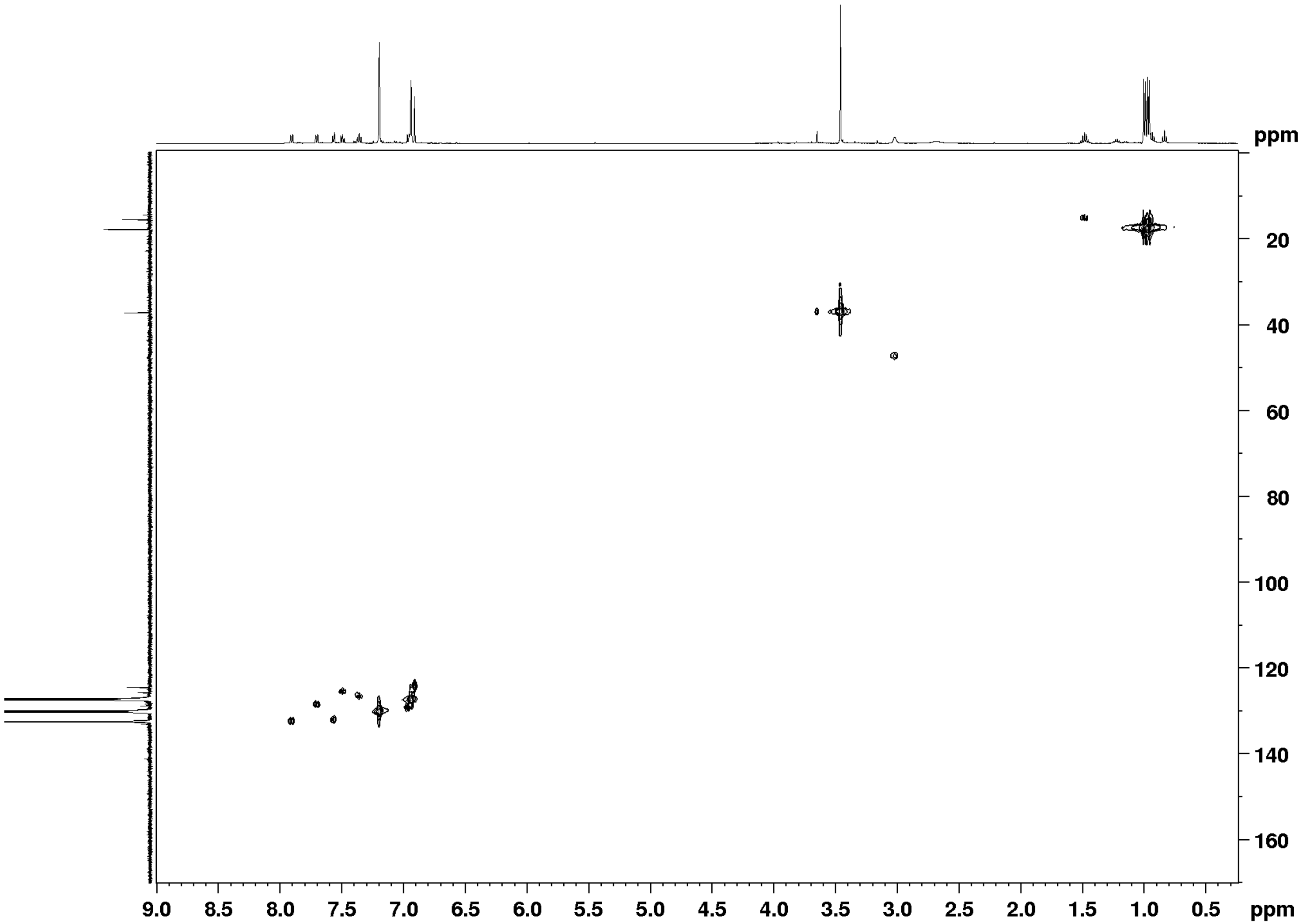

Figure S33. $^{1}H/^{13}C$ HSQC NMR (500/126 MHz, 1,2-$Cl_2C_6D_4$, 298 K) of Si/B hydronium carborate **10**$^{+}$[$HCB_{11}Cl_{11}$]$^{-}$.

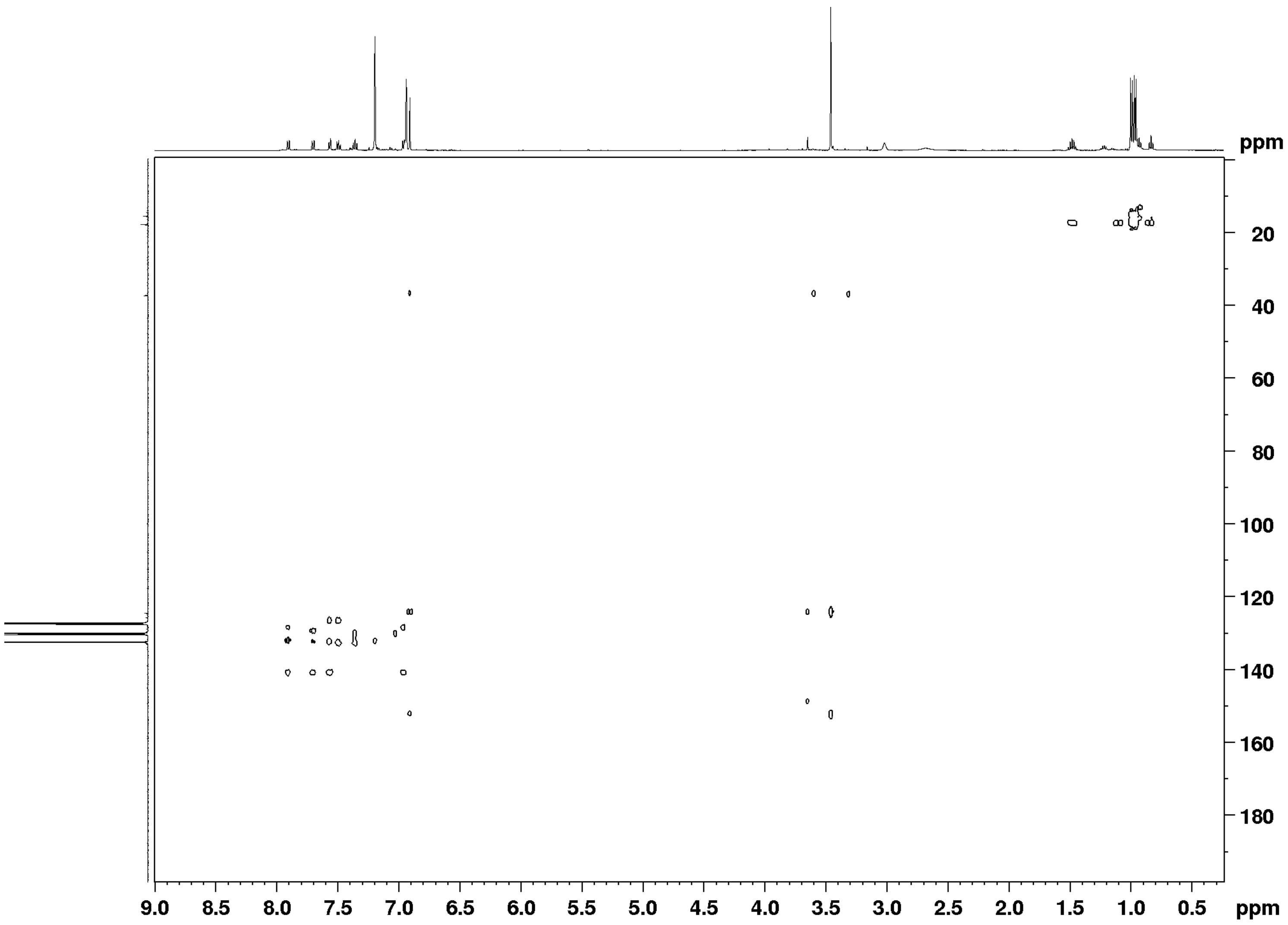

Figure S34. $^{1}H/^{13}C$ HMBC NMR (500/126 MHz, 1,2-$Cl_2C_6D_4$, 298 K) of Si/B hydronium carborate **10**$^{+}$[$HCB_{11}Cl_{11}$]$^{-}$.

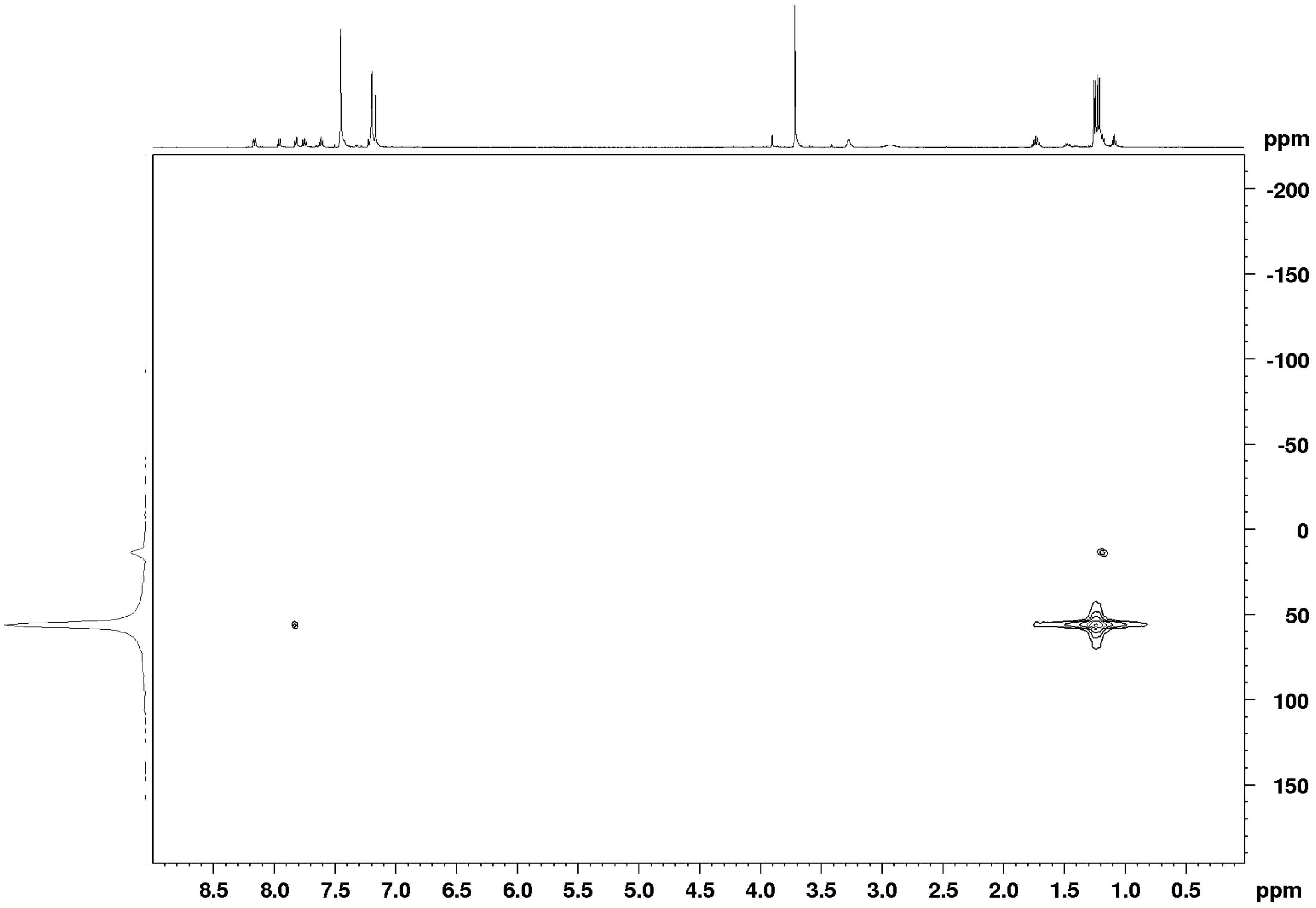

Figure S35. $^{1}H/^{29}Si$ HMQC NMR (500/99 MHz, 1,2-$Cl_2C_6D_4$, 298 K, optimized for *J* = 7 Hz) of Si/B hydronium carborate **10**$^{+}[HCB_{11}Cl_{11}]^{-}$.

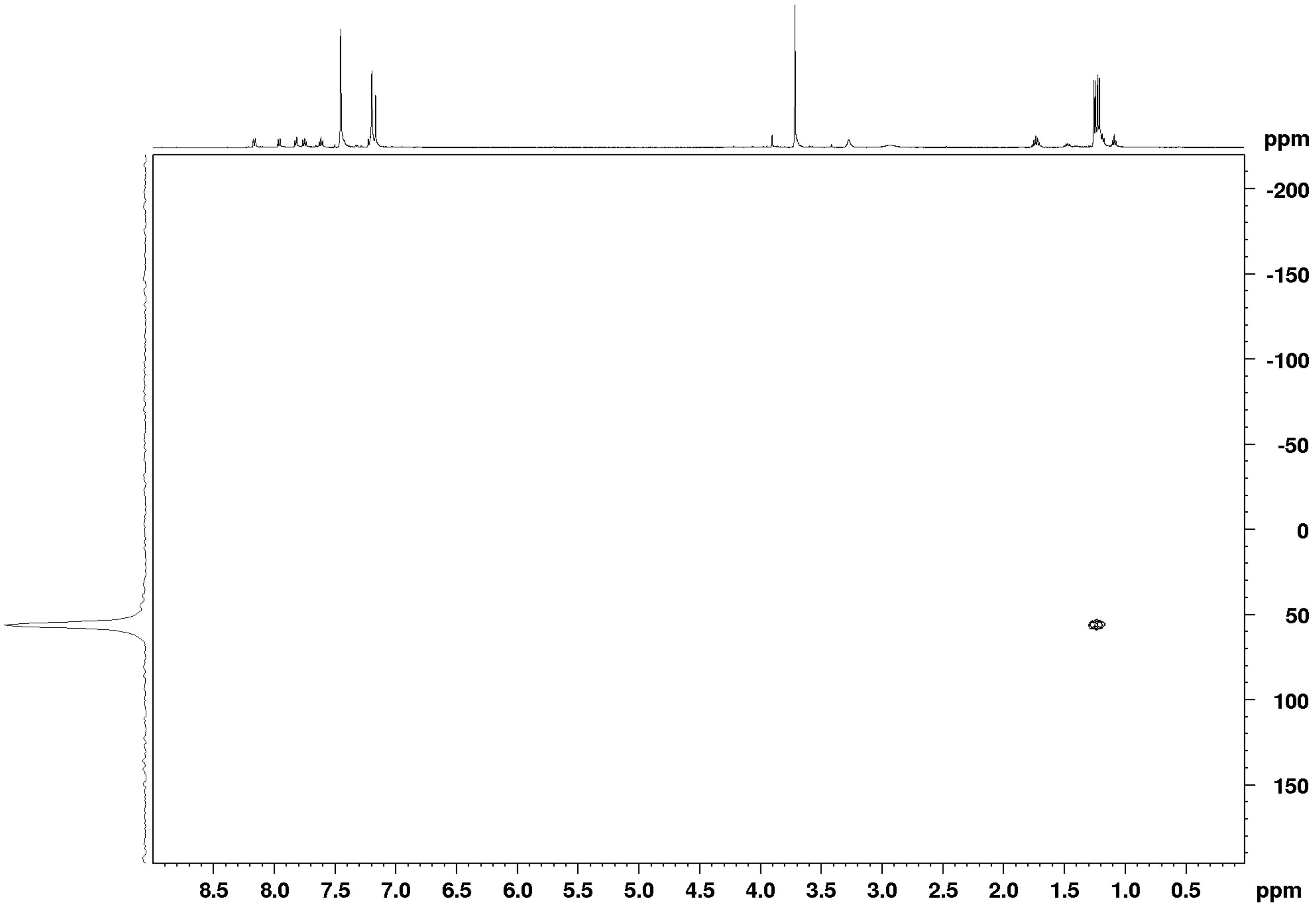

Figure S36. $^{1}H/^{29}Si$ HMQC NMR (500/99 MHz, 1,2-$Cl_2C_6D_4$, 298 K, optimized for *J* = 45 Hz) of Si/B hydronium carborate **10**$^{+}$[$HCB_{11}Cl_{11}$]$^{-}$.

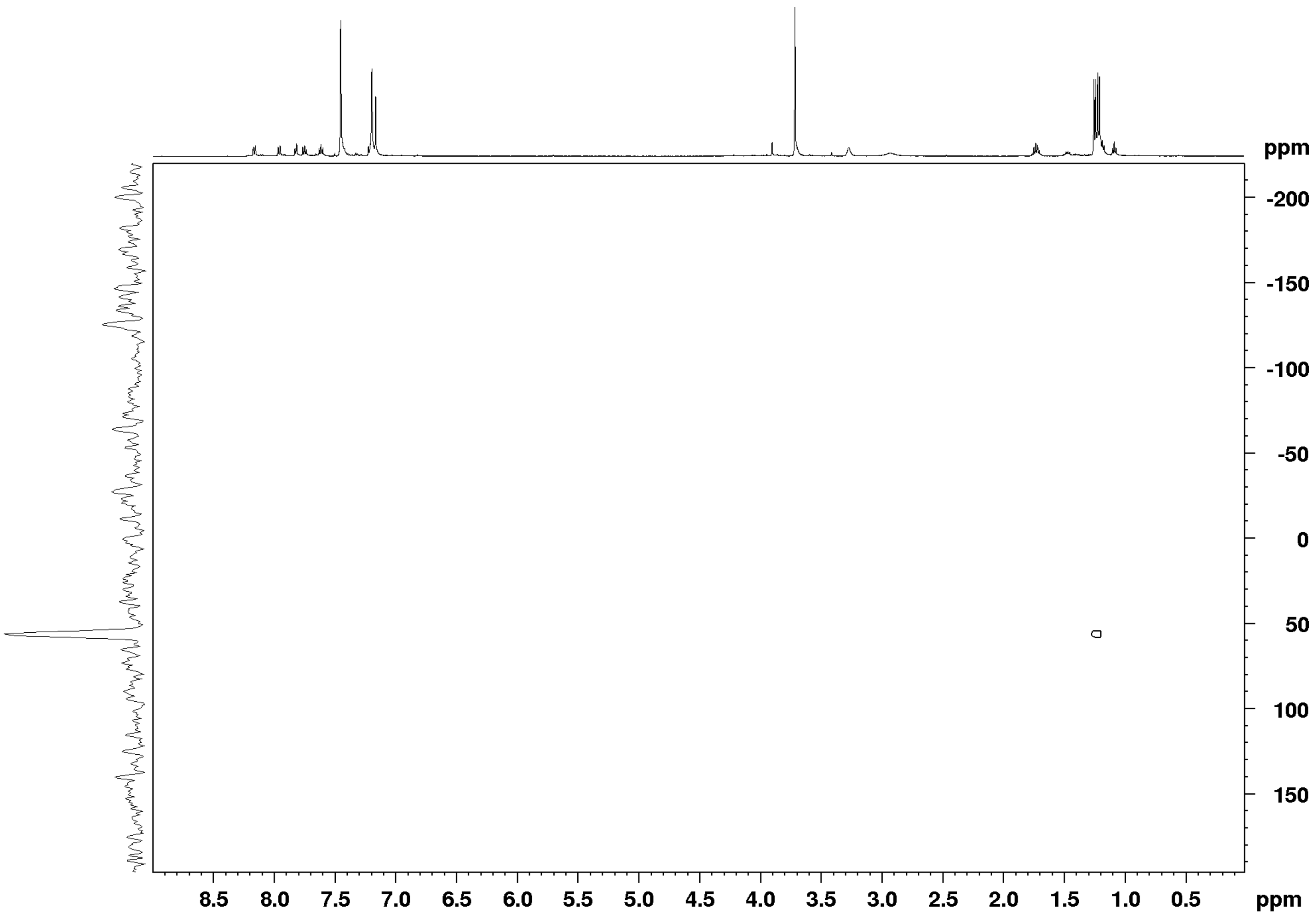

Figure S37. $^{1}H/^{29}Si$ HMQC NMR (500/99 MHz, 1,2-$Cl_2C_6D_4$, 298 K, optimized for *J* = 200 Hz) of Si/B hydronium carborate **10**$^{+}[HCB_{11}Cl_{11}]^{-}$.

7 Crystallographic Data

Data for the single-crystal structure determination were collected with an *Agilent* SuperNova diffractometer equipped with a CCD area Atlas detector and a mirror monochromator by utilizing Cu-K_α radiation (λ = 1.5418 Å). Software packages used: CrysAlis PRO for data collection, cell refinement and data reduction,[S5] SHELXS-97 for structure solution,[S6] SHELXL-97 for structure refinement,[S7] and Mercury[S8] for graphics.

7.1 Molecular Structure of Precursor 9 (CCDC 2116775)

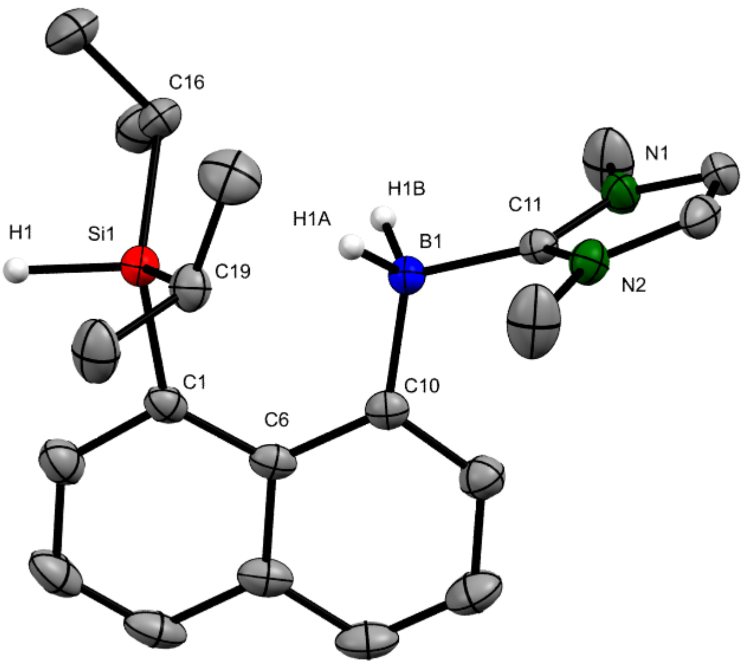

Figure S38. Molecular structure of precursor **9** (thermal ellipsoids are shown at 50% probability; hydrogen atoms except H1, H1A and H1B are omitted for clarity). Selected bond length (Å) and angles (°): Si1–H1 1.42(2), Si1–C16 1.898(1), Si1–C19 1.904(2), Si1–C1 1.904(1), B1–H1A 1.15(2), B1–H1B 1.12(2), B1–C11 1.606(2), Si1···B1 3.190(2); Si1–C1–C6 130.7(1), B1–C10–C6 123.0(1), C1–C6–C10 124.2(1).

Table S1. Crystal data and structure refinement.

Identification code	Cu-4401
Empirical formula	$C_{21}H_{31}BN_2Si$
Formula weight	350.38
Temperature	150.01(10) K
Wavelength	1.54184 Å
Crystal system	Monoclinic
Space group	$P2_1/c$

Unit cell dimensions	a = 10.93159(19) Å	α = 90°
	b = 13.8079(3) Å	β = 92.6310(16)°
	c = 13.7007(2) Å	γ = 90°
Volume	2065.84(6) $Å^3$	
Z	4	
Density (calculated)	1.127 mg/m^3	
Absorption coefficient	1.020 mm^{-1}	
F(000)	760	
Crystal size	0.22 × 0.17 × 0.09 mm^3	
Theta range for data collection	4.05 to 67.48°	
Index ranges	–12<=h<=13, –16<=k<=15, –10<=l<=16	
Reflections collected	7539	
Independent reflections	3726 [R(int) = 0.0174]	
Completeness to theta = 67.48°	99.9 %	
Absorption correction	Semi-empirical from equivalents	
Max. and min. transmission	0.9138 and 0.8098	
Refinement method	Full-matrix least-squares on F^2	
Data / restraints / parameters	3726 / 0 / 244	
Goodness-of-fit on F^2	1.062	
Final R indices [I>2sigma(I)]	R1 = 0.0351, wR2 = 0.0878	
R indices (all data)	R1 = 0.0394, wR2 = 0.0916	
Largest diff. peak and hole	0.233 and –0.251 e.$Å^{-3}$	

7.2 Molecular Structure of Si/B Hydronium Borate $10^{+}[B(C_6F_5)_4]^{-}$ (CCDC 2116777)

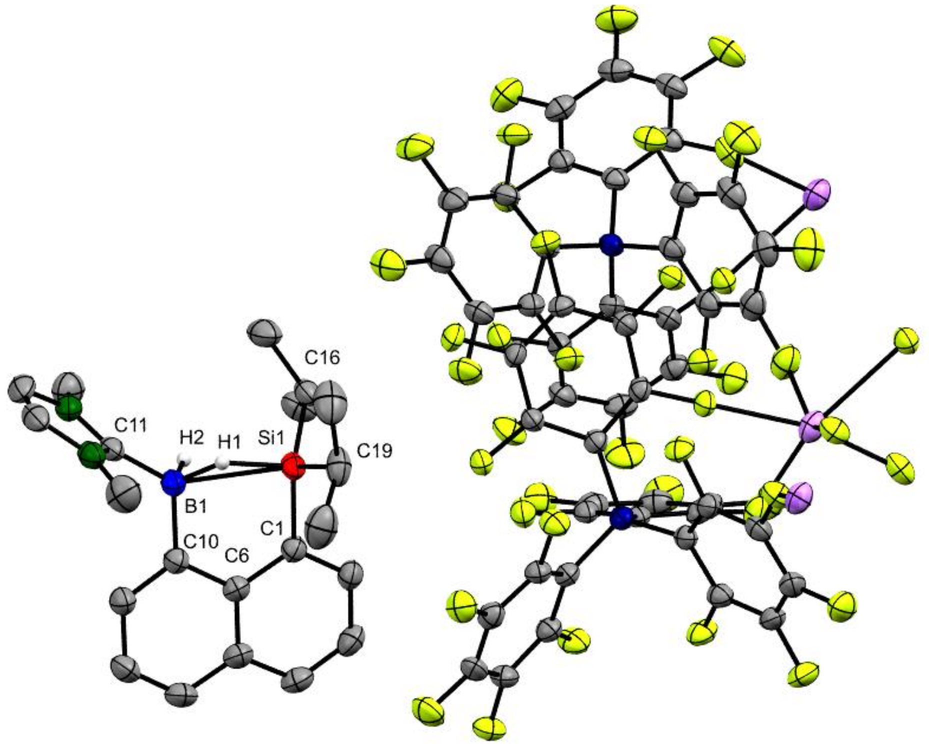

Figure S39. Molecular structure of **10**$[B(C_6F_5)_4]\cdot Li_{0.71}Na_{0.29}[B(C_6F_5)_4]$ (thermal ellipsoids are shown at 50% probability; hydrogen atoms except H1 and H2 are omitted for clarity). Selected bond length (Å) and angles (°): Si1–H1 1.59(2), Si1–C16 1.866(2), Si1–C19 1.875(3), Si1–C1 1.864(2), Si1–B1 2.458(2), B1–H1 1.33(2), B1–H2 1.08(2), B1–C11 1.586(3); Si1–H1–B1 114(1), C16–Si1–C1 118.46(9), C16–Si1–C19 115.6(1), C19–Si1–C1 111.39(9), Si1–C1–C6 118.2(1), C1–C6–C10 119.9(2), B1–C10–C6 116.6(2), H2–B1–H1104(1), C11–B1–H1 100.9(9), C11–B1–H2 111(1).

Table S2. Crystal data and structure refinement.

Identification code	Cu-4436	
Empirical formula	$C_{69}H_{30}B_3F_{40}Li_{0.71}N_2Na_{0.29}Si$	
Formula weight	1719.06	
Temperature	150.01(10) K	
Wavelength	1.54184 Å	
Crystal system	Triclinic	
Space group	P-1	
Unit cell dimensions	a = 12.9487(4) Å	α = 79.792(3)°
	b = 13.9150(4) Å	β = 81.725(3)°
	c = 19.8079(6) Å	γ = 71.060(3)°
Volume	3308.01(18) Å^3	
Z	2	

Density (calculated)	1.726 mg/m^3
Absorption coefficient	1.809 mm^{-1}
F(000)	1705
Crystal size	0.28 × 0.07 × 0.05 mm^3
Theta range for data collection	3.39 to 67.48°
Index ranges	–14<=h<=15, –16<=k<=16, –23<=l<=23
Reflections collected	23429
Independent reflections	11918 [R(int) = 0.0228]
Completeness to theta = 67.48°	99.9 %
Absorption correction	Semi-empirical from equivalents
Max. and min. transmission	0.9214 and 0.6314
Refinement method	Full-matrix least-squares on F^2
Data / restraints / parameters	11918 / 0 / 1060
Goodness-of-fit on F^2	1.018
Final R indices [I>2sigma(I)]	R1 = 0.0326, wR2 = 0.0802
R indices (all data)	R1 = 0.0436, wR2 = 0.0876
Largest diff. peak and hole	0.269 and –0.337 e. Å^{-3}

8 Computational Details

Structure optimizations were performed with the Turbomole program, version 7.5.0,[S9–11] at the DFT-TPSSh[S12,S13] level with def2-TZVP[S14] basis sets for all atoms, and employing the multipole-accelerated resolution-of-identity approximation (MARIJ)[S15–18] as well as the D3 empirical dispersion correction[S19] with Becke-Johnson damping (BJ).[S20] The counteranion of **10**[$B(C_6F_5)_4$] was neglected in all computations due to its experimentally known minor influence. In addition to gas phase calculations, all structures were subsequently optimized using the COSMO continuum solvent model,[S21,22] employing a relative permittivity ε = 9.9949 for 1,2-dichlorobenzene (1,2-$Cl_2C_6H_4$, *o*-DCB). For compounds **9** and **10**$^+$, conformational pre-screenings were performed using the Crest program, version 2.7.1,[S23–25] employing the GFN2-xTB[S26] extended tight binding method and standard program settings. The obtained structure ensemble was subsequently re-optimized at the higher DFT level described above to identify the minimum-energy conformer. Harmonic vibrational frequency calculations (analytical (gas phase) or numerical (COSMO) second derivatives of analytical gradients) have been performed to verify that the structures are true minima on the potential energy surface. Based on these calculations, the IR spectrum of **10**$^+$ was simulated using a Lorentz-type line shape and a convolution of the raw intensities with 11 cm^{-1} broadening. Additionally, based on the experimental structure of **10**[$B(C_6F_5)_4$], a model **10**$^+_{\mathbf{cs}}$ was considered, in which only the hydrogen atoms were re-optimized at MARIJ-TPSSh-D3(BJ)/def2-TZVP[/COSMO(*o*-DCB)] level, while keeping all other atomic coordinates fixed at their original position.

Free energy of solvation contributions (ΔG_{solv}) to the total Gibbs free energy of reaction ($\Delta_{rx}G_{soln}$) and activation ($\Delta_{TS}G_{soln}$) for the terminal-to-bridging hydrogen exchange reaction at boron as well as the NHC shift from boron to silicon in **10**$^+$ in *o*-DCB solution at standard state conditions (298.15 K, 0.1 MPa) were obtained using the COSMO-RS solvation model.[S27–30] To this end, additional single point calculations at the COSMO(*o*-DCB) and gas-phase optimized structures were carried out. These calculations were performed at the MARIJ-BP86[S31,S32] level with COSMO, setting an infinite permittivity and using the refined COSMO cavity construction algorithm (keyword $cosmo_isorad),[S28] as well as in the gas phase, employing def2-TZVPD[S14] basis sets for all atoms. Based on these single point calculations, subsequent COSMO-RS computations to obtain ΔG_{solv} used the COSMOtherm program, version C30_1201, and a BP-TZVPD-FINE level parameterization (BP_TZVPD_FINE_HB2012_C30_1201). A further refinement of $\Delta_{rx}G_{soln}$ and $\Delta_{TS}G_{soln}$ used total electronic energies obtained from single-point calculations at the gas-phase optimized structures at MARIJ-ωB97M-V[S33]/def2-TZVPP[S14] level.

Fluoride ion affinities (FIAs) of the silicon and boron centers in **10**$^+$ were computed employing the isodesmic reaction F_2CO + **10**$^+$–F → F_3CO^- + **10**$^+$. The final FIA values were obtained by adding the experimentally known FIA of F_2CO (209 kJ mol^{-1}).[S34,S35]

NMR nuclear shieldings for **10**$^+$ and **10**$^+_{cs}$ have been obtained from single-point calculations at the optimized (COSMO(*o*-DCB) or gas phase) structures using the current-density variant[S36] of the B97M-V[S37] functional (cB97M-V) in conjunction with pcSseg-2[S38] basis sets for all atoms. The obtained ^{29}Si and ^{11}B shieldings were converted to chemical shifts (δ, in ppm) relative to the shielding of tetramethylsilane ($Si(CH_3)_4$, TMS; gas phase: σ[^{29}Si] = 367.3; *o*-DCB(COSMO): σ[^{29}Si] = 366.9) and boron trifluoride diethyl etherate ($BF_3{\cdot}OEt_2$; gas phase: σ[^{11}B] = 101.8; *o*-DCB(COSMO): σ[^{11}B] = 102.8) as standard, computed at identical computational level. All nuclear shielding calculations have been performed with a local developers' version of the Turbomole program, based on version 7.5.0.

Further electronic structure analyses used the NBO 7.0.7[S39] program linked to single point TPSSh/def2-TZVP calculations at the optimized structures (gas phase and *o*-DCB (COSMO)) using Gaussian 09, revision D.01.[S40] At this computational level natural bond orbital (NBO)[S41,S42] analyses, natural population analyses (NPA),[S41] and calculations of Wiberg bond indices (WBIs)[S43,S44] were performed for compounds **9**, **10**$^+$, and **10**$^+_{cs}$. In addition, at the same level **10**$^+$ and **10**$^+_{cs}$ were also examined within the framework of natural resonance theory (NRT).[S41] As all calculated properties show only a minor dependence on the structure, only results based on the gas-phase optimized structures are listed in Tables S5–S7.

Atoms-in-molecules (AIM)[S45] calculations and plots of AIM-based molecular graphs were done with the AIM2000 program, version 2.0.[S46]

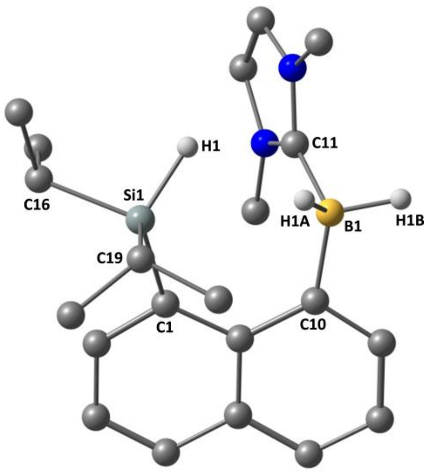

9

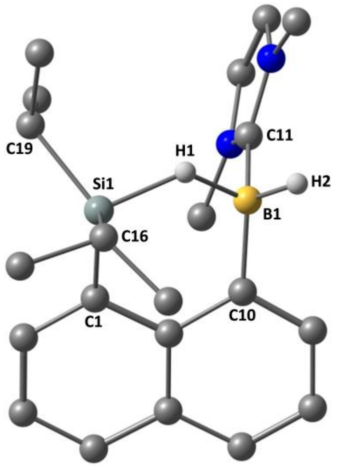

10^+

Figure S40. Optimized molecular structures of **9** and **10**$^+$ with atom labels.

Table S3. Selected structural parameters for **9**, **10**$^+$, and **10**$^+_{cs}$ from structure optimizations at the MARIJ-TPSSh-D3(BJ)/def2-TZVP level in the gas phase (GP) or *o*-DCB (COSMO). Distances (*d*) are given in Å, angles (*α*) in degree [°]. Experimental values are in italics.

	**9**		**10**$^+$		**10**$^+_{cs}$	
	**GP**	**COSMO**	**GP**	**COSMO**	**GP**	**COSMO**
***d*(Si1–H1)**	1.476<br>*1.42(2)*	1.473<br>*1.42(2)*	1.586<br>*1.59(2)*	1.578<br>*1.59(2)*	1.620<br>*1.59(2)*	1.617<br>*1.59(2)*
***d*(B1–H1A)**	1.214<br>1.15(2)	1.216<br>1.15(2)				
***d*(B1–H1B)**	1.224<br>*1.12(2)*	1.228<br>*1.12(2)*				
***d*(B1–H1)**			1.356<br>*1.33(2)*	1.366<br>*1.33(2)*	1.343<br>*1.33(2)*	1.343<br>*1.33(2)*
***d*(B1–H2)**			1.199<br>*1.08(2)*	1.199<br>*1.08(2)*	1.204<br>*1.08(2)*	1.204<br>*1.08(2)*
***d*(Si1···B1)**	3.331<br>*3.190(2)*	3.359<br>*3.190(2)*	2.654<br>*2.458(2)*	2.656<br>*2.458(2)*		
***d*(Si1–C1)**	1.895<br>*1.904(1)*	1.897<br>*1.904(1)*	1.845<br>*1.864(2)*	1.849<br>*1.864(2)*		
***d*(Si1–C16)**	1.906<br>*1.898(1)*	1.903<br>*1.898(1)*	*1.873*<br>*1.866(2)*	*1.875*<br>*1.866(2)*		
***d*(Si1–C19)**	1.901<br>*1.904(2)*	1.901<br>*1.904(2)*	1.877<br>*1.875(3)*	1.874<br>*1.875(3)*		
***d*(B1–C11)**	1.606<br>*1.606(2)*	1.609<br>*1.606(2)*	1.601<br>*1.586(3)*	1.599<br>*1.586(3)*		
***α*(Si1–H1–B1)**			128,7<br>*114(1)*	128.7<br>*114(1)*		

Table S4. Calculated ^{29}Si, ^{11}B, and ^{1}H NMR chemical shifts[a] (δ, in ppm) of **10**$^{+}$ and **10**$^{+}_{cs}$ (cB97M-V/pcSseg-2//MARIJ-TPSSh-D3(BJ)/def2-TZVP).[b]

	o-DCB				gas phase			
	**δ(^{29}Si)**	**δ(^{11}B)**	**δ(^{1}H)** *(B–)H1*	**δ(^{1}H)** *(B–)H2(–Si)*	**δ(^{29}Si)**	**δ(^{11}B)**	**δ(^{1}H)** *(B–)H1*	**δ(^{1}H)** *(B–)H2(–Si)*
**10**$^{+}$	55.3	–8.5	4.89	1.40	58.5	–10.5	4.84	1.32
**10**$^{+}_{cs}$	58.8	–19.9	4.30	1.79	59.8	–18.1	4.32	1.88
exp.	56.0	–8.2		2.65				

[a] Only the terminal and bridging hydrogen atoms at boron have been considered. See Figure S40 for atom labels.
[b] The counteranion $[B(C_6F_5)_4]^-$ was neglected in all calculations.

A

B

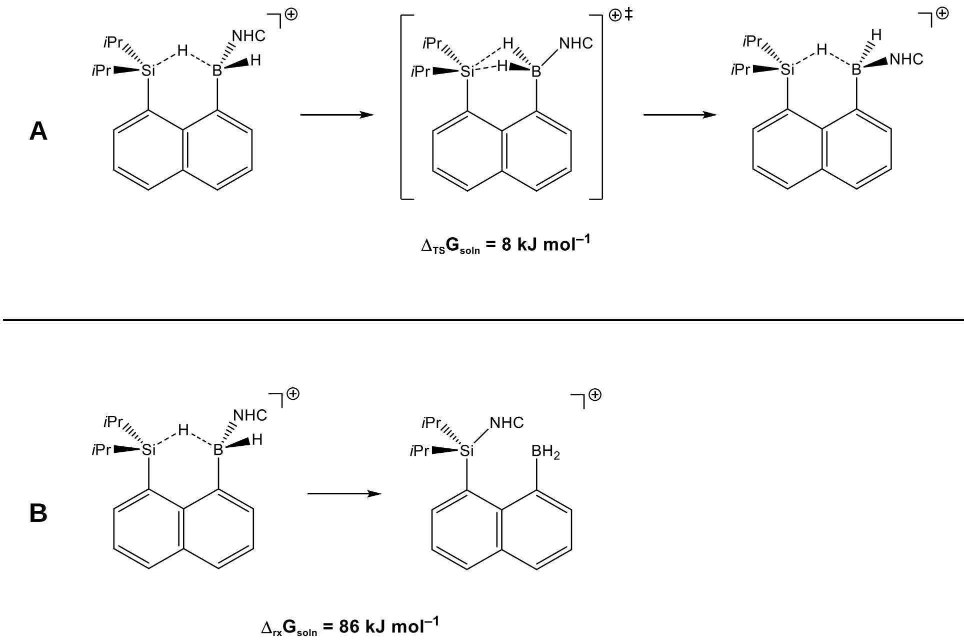

Scheme S1. Free energy of reaction ($\Delta_{rx}G_{soln}$) and activation ($\Delta_{TS}G_{soln}$) in *o*-DCB solution (298.15 K, 0.1 MPa) for the hydrogen exchange reaction from a bridging to a terminal position at boron (**A**) and the NHC shift from boron to silicon (**B**) in **10**$^{+}$. $\Delta_{rx}G_{soln}$ and $\Delta_{TS}G_{soln}$ values calculated from MARIJ-ωB97M-V/def2-TZVPP and MARIJ-BP86/def2-TZVPD/COSMO-RS(*o*-DCB) single point calculations at MARIJ-TPSSh-D3(BJ)/def2-TZVP structures optimized in the gas phase and *o*-DCB (COSMO). The counteranion $[B(C_6F_5)_4]^-$ was neglected in all calculations.

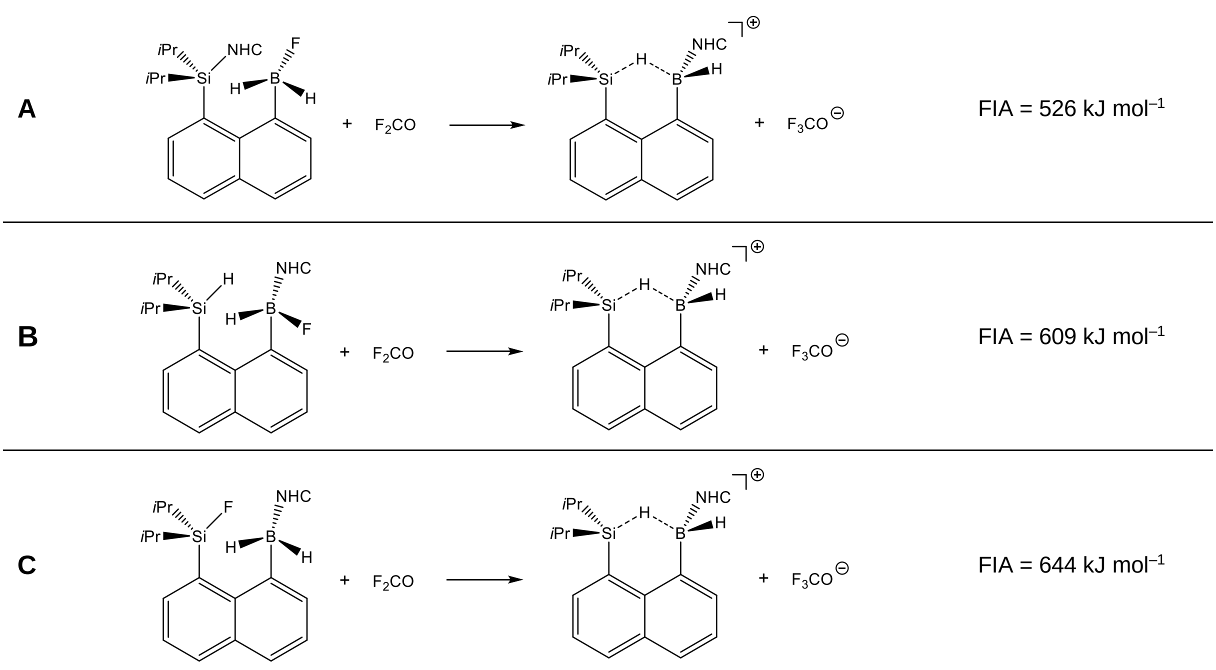

Scheme S2. Isodesmic reactions used to compute the fluoride ion affinity (FIA) of the boron center after boron-to-silicon NHC migration (**A**) and of the silicon (**B**) and boron center (**C**) in **10**$^+$ (MARIJ-TPSSh-D3(BJ)/def2-TZVP).

Table S5. Natural bond orders from NRT analyses and NBO Wiberg bond indices (WBIs) for the B–H–Si 3c2e bond, the terminal B–H bond, and the B···Si interaction in **10**$^+$ and **10**$^+_{cs}$ (TPSSh/def2-TZVP//MARIJ-TPSSh-D3(BJ)/def2-TZVP).

	**10**$^+$ natural bond order				**10**$^+_{cs}$ natural bond order			
	**total**	**covalent**	**ionic**	**WBI**	**total**	**covalent**	**ionic**	**WBI**
**B–H$_{terminal}$**	0.978	0.966	0.013	0.939	0.953	0.945	0.008	0.913
**B–H$_{bridge}$**	0.467	0.293	0.177	0.528	0.510	0.368	0.144	0.585
**Si–H$_{bridge}$**	0.365	0.191	0.174	0.397	0.306	0.144	0.162	0.344
**B···Si**	0.169	0.138	0.032	0.155	0.206	0.132	0.074	0.197

Table S6. NPA charges (q, in a.u.) of selected atoms in **9**, **10**$^+$, and **10**$^+_{cs}$ (TPSSh/def2-TZVP//MARIJ-TPSSh-D3(BJ)/def2-TZVP).

	**9**	**10**$^+$	**10**$^+_{cs}$
**q(Si)**	+1.503	+1.801	+1.823
**q(B)**	–0.256	+0.057	–0.058
**q((B)H$_{terminal}$)**	–0.002/+0.006	+0.030	+0.029
**q((Si)H)**	–0.227		
**q(H$_{bridge}$)**		–0.128	–0.073

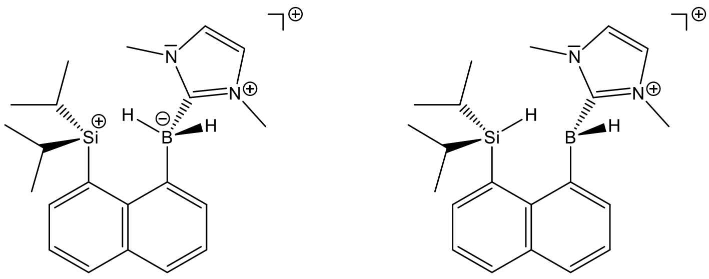

LS_{BH1} **LS_{SiH1}**

Figure S41. Lewis structures LS_{BH1} (left) and LS_{SiH1} (right) used for NBO analyses, featuring either an explicit B–H1 or Si–H1 bond.

Table S7. Occupancies of selected NBOs and the total non-Lewis density (NL) for the resonance Lewis structures LS_{SiH1} and LS_{BH1} of **10**$^+$ (TPSSh/def2-TZVP//MARIJ-TPSSh-D3(BJ)/def2-TZVP).

**LS_{SiH1}**	**σ(Si–H$_{bridge}$)**	**σ*(Si–H$_{bridge}$)**	**LV(p-AO(B))**	**σ(B–H$_{terminal}$)**	**NL**
	1.497	0.068	0.529	1.947	4.125
**LS_{BH1}**	**σ(B–H$_{bridge}$)**	**σ*(B–H$_{bridge}$)**	**LV(p-AO(Si))**	**σ(B–H$_{terminal}$)**	**NL**
	1.613	0.047	0.383	1.950	3.989

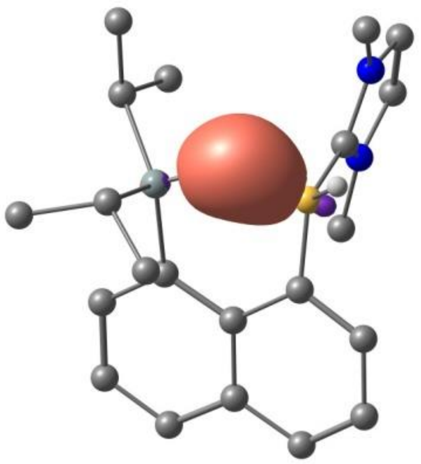

Figure S42. Orbital plot (isosurface value 0.05 a.u.) of the B–H–Si 3c2e NBO in **10**$^+$ (TPSSh/def2-TZVP).

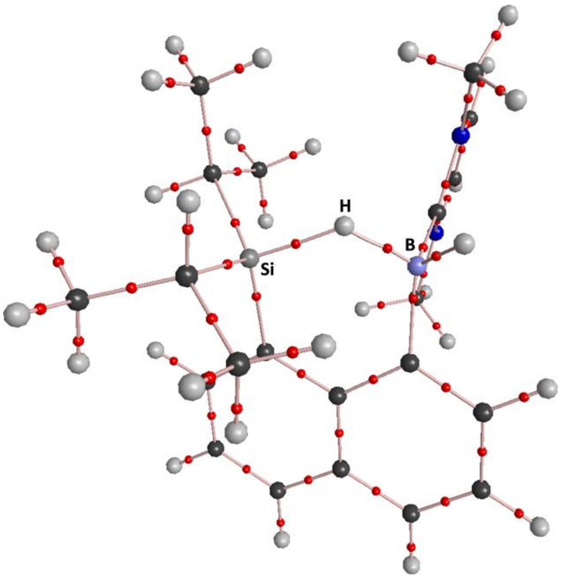

Figure S43. AIM-based molecular graph of **10**$^{+}$.

Table S8. Electron density (ρ), Laplacian ($\nabla^2\rho$), and ellipticity (ε) at selected bond critical points (BCPs) from AIM analysis of **10**$^{+}$ (B3LYP/def2-TZVP//MARIJ-TPSSh-D3(BJ)/def2-TZVP).

Bond	ρ	$\nabla^2\rho$	ε
**Si–H$_{bridge}$**	0.0824	0.0243	0.0367
**B–H$_{bridge}$**	0.0971	0.0124	0.4862
**B–H$_{terminal}$**	0.1761	-0.0788	0.0825

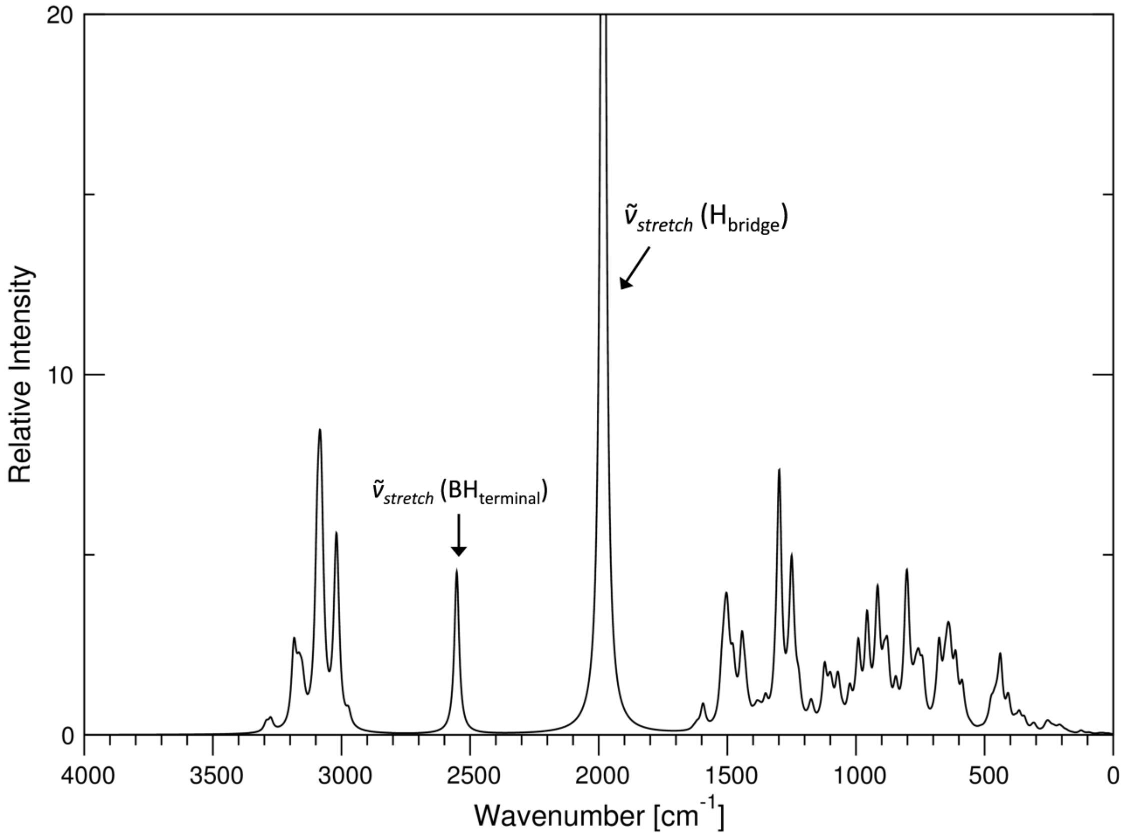

Figure S44. Calculated IR spectrum of **10**$^{+}$ (MARIJ-TPSSh-D3(BJ)/def2-TZVP).

Table S9. Cartesian coordinates of all considered compounds, optimized at the MARIJ-TPSSh-D3(BJ)/def2-TZVP level in the gas phase or *o*-DCB (COSMO).

**10^+ (GP)**				**10^+ (COSMO(*o*-DCB))**				**10^+-TS (GP)**			
C	-1.821349	2.652856	0.030661	C	-1.838203	2.665236	0.032735	C	2.577073	-1.601309	-0.181406
C	-2.962971	2.636248	0.868643	C	-2.985885	2.623259	0.862255	C	3.866423	-1.024449	-0.106987
C	-3.383949	1.475657	1.470139	C	-3.396899	1.447174	1.441731	C	4.024229	0.324282	0.109186
C	-2.701849	0.266688	1.225825	C	-2.698053	0.249584	1.183785	C	2.899632	1.159926	0.255964
C	-1.583746	0.232511	0.409973	C	-1.575097	0.241430	0.374359	C	1.617377	0.641363	0.187812
C	-1.101611	1.438820	-0.180282	C	-1.103525	1.462369	-0.193381	C	1.441878	-0.756782	-0.028517
C	0.091678	1.438160	-0.959240	C	0.096624	1.487842	-0.962031	C	0.126479	-1.300656	-0.090039
C	0.472593	2.617986	-1.572821	C	0.472480	2.683233	-1.548090	C	-0.024907	-2.652095	-0.307782
C	-0.255483	3.814165	-1.400206	C	-0.269695	3.869208	-1.359866	C	1.097836	-3.498176	-0.464422
C	-1.368190	3.837964	-0.596897	C	-1.392324	3.867117	-0.568867	C	2.368960	-2.986650	-0.401216
H	-1.918550	4.759029	-0.440812	H	-1.952893	4.779828	-0.398053	H	3.230052	-3.634929	-0.519455
H	0.077408	4.717999	-1.896357	H	0.062950	4.787054	-1.830933	H	0.947681	-4.557611	-0.635488
H	1.366782	2.638197	-2.187666	H	1.377226	2.725693	-2.146120	H	-1.017719	-3.090158	-0.364354
B	1.086931	0.206439	-1.026303	B	1.100835	0.263422	-1.046827	B	-1.041705	-0.244325	0.115443
C	2.260808	0.164418	0.061785	C	2.244092	0.167419	0.062444	C	-2.546255	-0.738030	0.126931
N	2.420694	0.865018	1.209125	N	2.404996	0.842332	1.224559	N	-3.153904	-1.393654	1.137471
C	3.603737	0.517241	1.819563	C	3.555897	0.433671	1.856333	C	-4.447055	-1.719490	0.787441
C	4.197276	-0.418815	1.035418	C	4.126347	-0.516677	1.069864	C	-4.638243	-1.259380	-0.474630
N	3.362966	-0.618387	-0.038803	N	3.310878	-0.662136	-0.025288	N	-3.457285	-0.663014	-0.865456
C	3.629722	-1.555263	-1.130630	C	3.567358	-1.586401	-1.129918	C	-3.220892	-0.051173	-2.174166
H	3.596731	-1.028225	-2.081679	H	3.704690	-1.026033	-2.052570	H	-3.196412	1.034268	-2.081948
H	4.617833	-1.981520	-0.976542	H	4.468828	-2.145727	-0.895486	H	-2.276274	-0.412243	-2.576051
H	2.885668	-2.350808	-1.130214	H	2.730676	-2.273876	-1.241463	H	-4.031487	-0.342123	-2.837841
H	5.129971	-0.943874	1.144442	H	5.029578	-1.088410	1.191292	H	-5.495393	-1.310753	-1.122878
H	3.920611	0.965503	2.744731	H	3.865968	0.852255	2.797586	H	-5.105407	-2.249224	1.453516
C	1.486886	1.844228	1.777168	C	1.502427	1.848640	1.791721	C	-2.525142	-1.701617	2.422972
H	1.319557	2.650815	1.067617	H	1.377236	2.669766	1.090625	H	-2.951949	-2.626186	2.805714
H	1.935366	2.233547	2.687851	H	1.954371	2.209680	2.711471	H	-2.705884	-0.891724	3.130114
H	0.540439	1.360814	2.008309	H	0.536504	1.398233	2.009160	H	-1.456537	-1.832794	2.266474
H	1.513810	-0.064727	-2.114405	H	1.540029	0.020562	-2.136617	H	-0.954553	0.629326	-0.762885
H	0.527322	-1.012717	-0.826481	H	0.518972	-0.966165	-0.921639	H	-0.861053	0.326028	1.204330
Si	-0.879206	-1.365678	-0.184832	Si	-0.839230	-1.348577	-0.215453	Si	0.094280	1.688264	0.346741
C	-1.762203	-2.059873	-1.684084	C	-1.752287	-2.071406	-1.685358	C	-0.194602	2.911750	-1.063616
C	-3.116564	-2.649908	-1.244343	C	-3.092104	-2.671981	-1.221143	C	0.569443	4.223466	-0.801517
H	-3.004058	-3.446428	-0.504164	H	-2.956753	-3.454837	-0.470467	H	0.243131	4.717168	0.115674
H	-3.763061	-1.876694	-0.819894	H	-3.742359	-1.901529	-0.796750	H	1.646879	4.048655	-0.731065
H	-3.635536	-3.072000	-2.108786	H	-3.615742	-3.112901	-2.074240	H	0.409339	4.919715	-1.628986
C	-1.945238	-0.997807	-2.778903	C	-1.961713	-1.023065	-2.787989	C	0.196701	2.315447	-2.422516
H	-0.990699	-0.592383	-3.121270	H	-1.014076	-0.615381	-3.147736	H	-0.322073	1.375273	-2.631612
H	-2.552933	-0.164418	-2.418462	H	-2.570431	-0.190383	-2.425827	H	1.268787	2.109931	-2.462052
H	-2.454865	-1.434735	-3.641744	H	-2.481103	-1.473648	-3.638838	H	-0.043112	3.013331	-3.228991
H	-1.140741	-2.872956	-2.079486	H	-1.126978	-2.879950	-2.082822	H	-1.268522	3.143677	-1.074153
C	-0.426530	-2.611861	1.143085	C	-0.368678	-2.578098	1.117509	C	-0.196538	2.507408	2.017133
C	0.195811	-3.892762	0.567091	C	0.257482	-3.860528	0.549050	C	-1.640997	3.013778	2.153979
H	-0.444275	-4.362258	-0.182303	H	-0.377754	-4.330136	-0.204910	H	-1.915977	3.705607	1.354181
H	0.369547	-4.622823	1.361755	H	0.420334	-4.586474	1.350402	H	-1.776838	3.534204	3.105597
H	1.162686	-3.684915	0.098400	H	1.228967	-3.655499	0.090107	H	-2.350002	2.178490	2.135467
C	0.438255	-2.013578	2.262282	C	0.488879	-1.977424	2.240400	C	0.203249	1.611117	3.193834
H	0.000856	-1.099457	2.670093	H	0.061181	-1.051340	2.631133	H	1.237390	1.270913	3.114943
H	0.547247	-2.728233	3.082129	H	0.571617	-2.685878	3.069047	H	0.090275	2.149753	4.138453
H	1.443166	-1.780821	1.899383	H	1.502642	-1.767275	1.890625	H	-0.435388	0.723057	3.248593
H	-1.400056	-2.879439	1.581730	H	-1.344769	-2.844430	1.551569	H	0.473451	3.378721	2.008315
H	-3.082686	-0.644310	1.676937	H	-3.068084	-0.671799	1.621941	H	3.061097	2.219448	0.426943
H	-4.253610	1.479349	2.115781	H	-4.270124	1.429766	2.083000	H	5.017499	0.752819	0.166966
H	-3.503183	3.562635	1.030424	H	-3.536091	3.541801	1.036712	H	4.734595	-1.664250	-0.221941
**10^+-TS (COSMO(*o*-DCB))**				**10^+_{cs} (GP)**				**10^+_{cs} (COSMO(*o*-DCB))**			
C	2.605908	-1.603002	-0.165895	C	2.070888	4.996746	-3.987025	C	2.070888	4.996746	-3.987025
C	3.891363	-1.015192	-0.090160	N	1.701909	4.891795	-2.662805	N	1.701909	4.891795	-2.662805
C	4.035406	0.337264	0.117892	C	1.394559	3.619876	-2.378156	C	1.394559	3.619876	-2.378156
C	2.902169	1.165333	0.255862	N	1.565834	2.931568	-3.522083	N	1.565834	2.931568	-3.522083
C	1.625283	0.635423	0.186560	C	1.982123	3.779815	-4.514389	C	1.982123	3.779815	-4.514389
C	1.462949	-0.765991	-0.022975	C	1.274398	1.515410	-3.731836	C	1.274398	1.515410	-3.731836
C	0.150258	-1.318240	-0.086876	C	1.682856	6.004502	-1.718468	C	1.682856	6.004502	-1.718468
C	0.008366	-2.672332	-0.295291	B	0.909760	3.011147	-0.995814	B	0.909760	3.011147	-0.995814
C	1.139262	-3.511473	-0.441272	C	-0.450348	3.523682	-0.361683	C	-0.450348	3.523682	-0.361683
C	2.408764	-2.992225	-0.377266	C	-0.750244	3.073171	0.962880	C	-0.750244	3.073171	0.962880
H	3.275327	-3.635460	-0.487261	C	-2.061730	3.223466	1.485056	C	-2.061730	3.223466	1.485056
H	0.995740	-4.573721	-0.603918	C	-3.024470	3.919591	0.713442	C	-3.024470	3.919591	0.713442
H	-0.981077	-3.116312	-0.349894	C	-2.704439	4.422844	-0.513077	C	-2.704439	4.422844	-0.513077
B	-1.027637	-0.268320	0.105692	C	-1.418995	4.193970	-1.060238	C	-1.418995	4.193970	-1.060238
C	-2.538198	-0.736224	0.127841	C	0.260657	2.445031	1.746196	C	0.260657	2.445031	1.746196
N	-3.144489	-1.400107	1.132808	C	-0.060811	1.919828	2.979137	C	-0.060811	1.919828	2.979137
C	-4.453774	-1.671919	0.802267	C	-1.388874	1.996274	3.462770	C	-1.388874	1.996274	3.462770
C	-4.656083	-1.166049	-0.441728	C	-2.348258	2.650192	2.752756	C	-2.348258	2.650192	2.752756
N	-3.465707	-0.598264	-0.840676	Si	2.022278	2.527065	1.141846	Si	2.022278	2.527065	1.141846
C	-3.248351	0.063458	-2.127732	C	3.004303	3.825321	2.071751	C	3.004303	3.825321	2.071751
H	-3.200586	1.142918	-1.987678	C	4.390063	4.074715	1.463366	C	4.390063	4.074715	1.463366
H	-2.324576	-0.301861	-2.571468	C	2.920387	0.927349	0.799347	C	2.920387	0.927349	0.799347
H	-4.083361	-0.181856	-2.778446	C	3.842306	1.003553	-0.427672	C	3.842306	1.003553	-0.427672
H	-5.526517	-1.167226	-1.074029	C	1.986209	-0.285868	0.701801	C	1.986209	-0.285868	0.701801
H	-5.113230	-2.200110	1.468359	C	2.224345	5.134315	2.230629	C	2.224345	5.134315	2.230629

C	-2.511552	-1.751469	2.403912	H	1.931252	3.404249	-0.216851	H	1.924965	3.406960	-0.210749
H	-2.892638	-2.717522	2.726685	H	0.982960	1.810035	-1.022649	H	0.978706	1.809517	-1.015499
H	-2.741698	-0.993935	3.152864	H	0.690730	1.440850	3.599124	H	0.692339	1.442161	3.597233
H	-1.436566	-1.817866	2.253960	H	-1.627959	1.555012	4.423098	H	-1.627923	1.555109	4.423687
H	-0.931669	0.595581	-0.780437	H	-3.355211	2.738468	3.146689	H	-3.355754	2.739242	3.146537
H	-0.837245	0.310115	1.190678	H	-4.021463	4.051878	1.119774	H	-4.021618	4.051419	1.121083
Si	0.081628	1.663098	0.335017	H	-3.443848	4.963410	-1.091853	H	-3.443183	4.965022	-1.092098
C	-0.198763	2.879642	-1.082446	H	-1.223558	4.538099	-2.072784	H	-1.220356	4.540598	-2.070482
C	0.556920	4.195621	-0.821557	H	2.165317	3.434082	-5.517013	H	2.161909	3.431526	-5.516629
H	0.219733	4.690579	0.091133	H	2.361151	5.936877	-4.423116	H	2.357946	5.938790	-4.420724
H	1.634681	4.026265	-0.739421	H	1.852731	0.908632	-3.038649	H	2.016944	0.899649	-3.226604
H	0.397537	4.885266	-1.655515	H	1.549456	1.264882	-4.753356	H	1.311323	1.323975	-4.800851
C	0.201622	2.279939	-2.436929	H	0.212376	1.328617	-3.580999	H	0.282000	1.284213	-3.352154
H	-0.314366	1.338502	-2.646036	H	0.984675	5.767259	-0.918666	H	0.987405	5.768555	-0.916495
H	1.275851	2.081005	-2.470301	H	1.347313	6.899464	-2.238528	H	1.345545	6.897638	-2.239168
H	-0.039697	2.976027	-3.245059	H	2.681993	6.167611	-1.313184	H	2.683272	6.168592	-1.317715
H	-1.273389	3.104400	-1.101346	H	3.560974	0.792755	1.684070	H	3.556918	0.796022	1.686337
C	-0.219456	2.494337	1.996780	H	2.573995	-1.199291	0.577706	H	2.577077	-1.197966	0.577234
C	-1.676705	2.968291	2.119347	H	1.316725	-0.198012	-0.157645	H	1.318290	-0.198889	-0.159301
H	-1.961959	3.642231	1.307765	H	1.363815	-0.400098	1.590608	H	1.366972	-0.399622	1.593646
H	-1.825818	3.498118	3.064359	H	4.406196	0.073342	-0.533302	H	4.414272	0.076469	-0.521563
H	-2.366028	2.116965	2.108771	H	4.559493	1.824314	-0.368346	H	4.550557	1.832302	-0.372264
C	0.187167	1.612945	3.182228	H	3.253975	1.138546	-1.339081	H	3.255323	1.125299	-1.341372
H	1.231561	1.300601	3.117130	H	3.140909	3.386282	3.072308	H	3.139873	3.383167	3.070281
H	0.048309	2.155660	4.121709	H	2.781971	5.832797	2.860024	H	2.787970	5.833695	2.854711
H	-0.429809	0.709623	3.235095	H	1.243512	4.976294	2.682297	H	1.247573	4.975433	2.692198
H	0.432584	3.378138	1.985337	H	2.071729	5.619106	1.262234	H	2.063083	5.617141	1.262881
H	3.053786	2.226948	0.420838	H	4.929908	4.828799	2.042530	H	4.927593	4.826683	2.048573
H	5.024880	0.775576	0.176503	H	4.310532	4.448631	0.437000	H	4.311774	4.451826	0.438556
H	4.765518	-1.648801	-0.197560	H	5.004365	3.172346	1.445130	H	5.000522	3.169578	1.444620
**10^{+}-Si-NHC (GP)**				**10^{+}-Si-NHC (COSMO(*o*-DCB))**				**9 (GP)**			
C	4.395515	0.526328	0.182465	C	4.398984	0.506054	0.189015	N	2.903578	-0.957269	-0.043262
C	4.112905	1.739373	0.757605	C	4.109945	1.721179	0.757406	C	2.051539	0.097755	-0.023237
C	2.788471	2.033229	1.126040	C	2.781118	2.020000	1.113797	N	2.182697	0.621146	1.220125
C	1.750176	1.119897	0.951161	C	1.745346	1.107979	0.934574	C	3.087739	-0.111363	1.968039
C	2.019934	-0.113914	0.267387	C	2.021680	-0.129480	0.263516	C	3.541892	-1.106182	1.170162
C	0.987197	-1.017270	-0.126728	C	0.987770	-1.032444	-0.127833	B	1.182268	0.521140	-1.305862
C	1.349332	-2.208335	-0.745268	C	1.345873	-2.226591	-0.740395	C	0.044963	1.628775	-1.047289
C	2.686484	-2.540005	-1.020810	C	2.684732	-2.561540	-1.011199	C	-1.097709	1.495715	-0.182266
C	3.679585	-1.643809	-0.716952	C	3.681075	-1.667799	-0.706857	C	-1.924927	2.655410	0.037198
C	3.370457	-0.416000	-0.088091	C	3.373674	-0.435896	-0.083261	C	-1.672389	3.864396	-0.653641
H	4.713846	-1.860427	-0.960326	H	4.715632	-1.887498	-0.947524	C	-0.622667	3.952923	-1.525971
H	2.920702	-3.484769	-1.495764	H	2.917046	-3.507904	-1.484808	C	0.225544	2.843518	-1.693449
H	0.580959	-2.914327	-1.045417	H	0.576721	-2.931451	-1.040316	C	-1.451065	0.283841	0.520016
Si	-0.865778	-0.784082	0.053531	Si	-0.863403	-0.776676	0.052329	C	-2.510424	0.328444	1.420310
C	-1.496146	-1.576286	1.678705	C	-1.488481	-1.576614	1.675094	C	-3.279065	1.481368	1.660099
C	-2.758206	-2.443411	1.553500	C	-2.731608	-2.467715	1.532007	C	-2.999263	2.619438	0.957290
H	-2.649811	-3.217670	0.790508	H	-2.590068	-3.246375	0.778459	Si	-0.948953	-1.521325	0.237103
H	-3.653814	-1.866266	1.314298	H	-3.628573	-1.904921	1.267408	C	-1.222889	-2.526882	1.832771
H	-2.945719	-2.948763	2.504572	H	-2.929821	-2.965298	2.485918	C	-0.367683	-1.956997	2.973571
C	-0.354813	-2.413222	2.290645	C	-0.343526	-2.388257	2.310982	C	1.480748	1.791824	1.741099
H	-0.684956	-2.835797	3.243166	H	-0.681807	-2.806504	3.263532	C	3.069770	-1.822125	-1.200813
H	0.539025	-1.814299	2.471516	H	0.537254	-1.771293	2.498670	C	-2.022583	-2.222048	-1.166013
H	-0.071687	-3.244258	1.639772	H	-0.037547	-3.219301	1.670068	C	-1.952236	-1.393821	-2.456535
H	-1.706198	-0.775295	2.390734	H	-1.728405	-0.776192	2.377293	C	-3.483057	-2.372041	-0.712733
C	-1.780011	-1.492195	-1.460299	C	-1.781889	-1.472863	-1.461941	C	-0.847312	-3.992847	1.564522
C	-3.236847	-0.998019	-1.521865	C	-3.229953	-0.954175	-1.524570	H	-2.327313	4.710358	-0.471130
H	-3.790819	-1.180342	-0.600847	H	-3.785192	-1.117795	-0.600470	H	-0.427701	4.873007	-2.066637
H	-3.272393	0.074901	-1.735722	H	-3.246468	0.117523	-1.745137	H	1.082630	2.941914	-2.350917
H	-3.772911	-1.504085	-2.329086	H	-3.774003	-1.459400	-2.327884	H	3.289162	-1.213475	-2.076677
C	-1.120472	-1.244649	-2.824983	C	-1.114819	-1.232299	-2.823942	H	3.889022	-2.511083	-1.002255
H	-1.220022	-0.196235	-3.118902	H	-1.190734	-0.181065	-3.113285	H	2.144914	-2.373060	-1.377145
H	-1.624459	-1.835924	-3.594394	H	-1.630829	-1.813154	-3.594345	H	4.255196	-1.890500	1.352131
H	-0.061489	-1.506878	-2.843441	H	-0.061218	-1.516039	-2.839556	H	3.324439	0.144534	2.985499
H	-1.802299	-2.576980	-1.287631	H	-1.818127	-2.556971	-1.289484	H	1.337541	2.510220	0.939602
C	-1.255266	1.116522	-0.102468	C	-1.233127	1.123560	-0.071345	H	2.092957	2.228283	2.529164
N	-2.266587	1.807593	0.489197	N	-2.263585	1.800781	0.508926	H	0.508422	1.502447	2.138706
C	-2.369992	3.066175	-0.044290	C	-2.396030	3.043626	-0.043593	H	2.005886	0.949268	-2.104068
C	-1.412053	3.169380	-1.001405	C	-1.438558	3.155612	-1.005002	H	0.751578	-0.515541	-1.768685
N	-0.746943	1.970685	-1.032669	N	-0.746743	1.976840	-1.018149	H	-3.581178	-3.021751	0.161231
C	0.404199	1.734498	-1.906237	C	0.401451	1.751833	-1.897721	H	-3.912798	-1.399187	-0.456260
H	1.276323	2.235360	-1.486360	H	1.265779	2.277283	-1.493202	H	-4.092682	-2.799051	-1.516449
H	0.597779	0.669652	-1.968751	H	0.614475	0.690190	-1.951290	H	-0.935488	-1.328773	-2.845903
H	0.174301	2.130074	-2.893739	H	0.155278	2.133926	-2.885572	H	-2.299176	-0.371762	-2.281699
H	-1.148938	3.985010	-1.652313	H	-1.194786	3.967256	-1.668257	H	-2.591723	-1.836341	-3.228685
H	-3.104749	3.773511	0.298833	H	-3.148011	3.737844	0.288774	H	-1.626205	-3.224521	-1.378504
C	-3.108678	1.333544	1.587588	C	-3.105090	1.324912	1.607190	H	-1.490977	-4.447137	0.806782
H	-2.535966	1.342084	2.514372	H	-2.518008	1.284414	2.523487	H	-0.932683	-4.592110	2.477720
H	-3.461928	0.328910	1.372510	H	-3.504865	0.342973	1.368126	H	0.186903	-4.073760	1.213698
H	-3.959472	2.005084	1.672419	H	-3.923979	2.028888	1.725031	H	-0.656465	-0.932411	3.221790
B	0.472037	1.427448	1.732708	B	0.439640	1.427435	1.678791	H	-0.461170	-2.564331	3.881003
H	0.179522	2.556577	1.992585	H	0.139225	2.562953	1.898876	H	0.690995	-1.945016	2.692884
H	-0.160223	0.549534	2.236444	H	-0.155053	0.564494	2.250674	H	-2.273863	-2.507513	2.141576
H	2.573187	2.979855	1.610481	H	2.564138	2.973686	1.583413	H	-2.787635	-0.570403	1.958447
H	4.901072	2.459230	0.940976	H	4.895863	2.444312	0.940220	H	-4.092322	1.450736	2.376416
H	5.413709	0.272238	-0.092090	H	5.418686	0.251139	-0.079766	H	-3.589318	3.520144	1.094781
								H	0.474166	-1.821099	-0.012712

9 (COSMO(*o*-DCB))

N	2.895899	-0.982745	-0.050921
C	2.058208	0.082320	-0.022692
N	2.191945	0.594488	1.224223
C	3.088277	-0.152989	1.967582
C	3.532072	-1.147241	1.161990
B	1.199546	0.549231	-1.300517
C	0.056119	1.653264	-1.030678
C	-1.105210	1.505765	-0.190185
C	-1.938194	2.662555	0.029003
C	-1.673407	3.884880	-0.635781
C	-0.603864	3.989412	-1.483075
C	0.247651	2.881226	-1.650369
C	-1.474800	0.284325	0.486575
C	-2.555491	0.316145	1.362151
C	-3.331987	1.464625	1.601133
C	-3.035318	2.614403	0.923034
Si	-0.961671	-1.522373	0.222048
C	-1.188588	-2.502784	1.836605
C	-0.332896	-1.896040	2.958112
C	1.503863	1.767191	1.760004
C	3.072385	-1.845422	-1.210553
C	-2.062177	-2.245491	-1.149139
C	-1.985820	-1.442579	-2.455190
C	-3.521741	-2.382179	-0.691652
C	-0.789084	-3.966717	1.589365
H	-2.332958	4.727146	-0.451918
H	-0.395500	4.919332	-2.002014
H	1.119864	2.997047	-2.285759
H	3.399554	-1.252215	-2.063464
H	3.821902	-2.595279	-0.967801
H	2.123647	-2.325885	-1.451522
H	4.236390	-1.941789	1.336205
H	3.326663	0.094979	2.986926
H	1.413984	2.519935	0.982563
H	2.096261	2.154647	2.586326
H	0.510390	1.491712	2.112104
H	2.026235	1.015192	-2.080254
H	0.767072	-0.464574	-1.813404
H	-3.616590	-3.004978	0.202041
H	-3.951737	-1.402331	-0.462370
H	-4.132058	-2.833931	-1.481706
H	-0.965796	-1.391284	-2.840395
H	-2.327607	-0.415019	-2.298619
H	-2.624024	-1.895129	-3.222957
H	-1.670112	-3.253053	-1.343755
H	-1.430788	-4.444609	0.844198
H	-0.858891	-4.550018	2.514275
H	0.244039	-4.035467	1.232760
H	-0.635338	-0.870973	3.187713
H	-0.412014	-2.487119	3.877588
H	0.723328	-1.873173	2.669755
H	-2.236436	-2.495930	2.156382
H	-2.842629	-0.589387	1.884193
H	-4.161112	1.422794	2.298643
H	-3.627496	3.513734	1.061006
H	0.449673	-1.802773	-0.092877

F_2CO (GP)

C	0.000000	-0.000000	0.225184
O	-0.000001	-0.000000	1.399335
F	1.065424	-0.000000	-0.555589
F	-1.065421	-0.000000	-0.555592

F_3CO^- (GP)

C	-0.000014	-0.000000	0.166504
O	-0.000024	-0.000000	1.382465
F	1.277344	-0.000000	-0.493013
F	-0.638652	-1.106211	-0.492976
F	-0.638652	1.106211	-0.492976

10^+-Si-F (GP)

C	-2.994001	2.603860	1.079642
C	-1.930500	2.627213	0.147002
C	-1.123095	1.456830	-0.088135
C	-1.469172	0.246778	0.627382
C	-2.521325	0.307677	1.536608
C	-3.278854	1.467364	1.780643
C	-1.671418	3.835376	-0.542475
C	-0.640330	3.912146	-1.438471
C	0.177984	2.786405	-1.636645
C	-0.008506	1.574916	-0.986510
B	1.081674	0.436785	-1.275310
C	2.168999	0.209158	-0.115023
N	3.134275	-0.736058	-0.212833
C	3.921659	-0.763171	0.918027
C	3.448321	0.202485	1.739988
N	2.380177	0.794761	1.088496
C	3.238214	-1.651290	-1.338621
C	1.589232	1.873432	1.675825
Si	-1.005862	-1.570283	0.359772
C	-1.877936	-2.239890	-1.181088
C	-3.383554	-2.337913	-0.858801
C	-1.552821	-2.609976	1.861617
C	-1.275168	-4.097584	1.580035
C	-0.807573	-2.174467	3.134396
C	-1.693249	-1.464755	-2.492127
H	-2.309861	4.690064	-0.343028
H	-0.443434	4.831839	-1.979210
H	1.012562	2.865453	-2.325454
H	3.186643	-1.081656	-2.265053
H	4.188487	-2.178520	-1.269986

10^+-Si-F (COSMO(*o*-DCB))

C	-3.060200	2.604680	0.938103
C	-1.934184	2.650943	0.080900
C	-1.112504	1.485689	-0.141643
C	-1.502236	0.260705	0.525513
C	-2.613465	0.299457	1.364670
C	-3.393649	1.448958	1.584939
C	-1.634579	3.885337	-0.544916
C	-0.546457	3.994978	-1.367300
C	0.277320	2.871413	-1.559304
C	0.051507	1.631224	-0.975703
B	1.151304	0.502743	-1.296546
C	2.178979	0.152172	-0.110503
N	3.135590	-0.796889	-0.240624
C	3.874572	-0.920443	0.917095
C	3.375924	-0.013797	1.790920
N	2.343098	0.640936	1.141870
C	3.302560	-1.616285	-1.432102
C	1.549557	1.689461	1.778970
Si	-1.050773	-1.568101	0.323030
C	-1.997126	-2.287476	-1.144847
C	-3.486878	-2.422855	-0.779168
C	-1.464487	-2.525638	1.911794
C	-1.126673	-4.014494	1.715727
C	-0.682437	-1.955451	3.106781
C	-1.853096	-1.509444	-2.459512
H	-2.285678	4.732248	-0.352854
H	-0.310741	4.934941	-1.855522
H	1.152160	2.979068	-2.192547
H	3.468460	-0.974820	-2.296917
H	4.159781	-2.269004	-1.282200

10^+-B-F (GP) (NHC shift)

N	-0.844303	2.033299	-1.225974
C	-1.415536	1.195953	-0.333952
N	-2.388849	1.916944	0.265033
C	-2.404418	3.206032	-0.221384
C	-1.432426	3.280322	-1.159764
Si	-0.904707	-0.632754	0.072330
C	-1.895006	-1.432011	-1.390158
C	-1.542457	-0.932839	-2.796877
C	-3.142126	1.464942	1.427897
C	0.369335	1.772278	-1.996667
C	0.925645	-0.938792	-0.233516
C	2.048633	-0.210078	0.303798
C	3.368799	-0.635381	-0.081666
C	3.542826	-1.749924	-0.936178
C	2.465195	-2.448422	-1.408418
C	1.168192	-2.030870	-1.055718
C	4.505667	0.050050	0.404415
C	4.358954	1.112767	1.258105
C	3.070195	1.517764	1.646824
C	1.914230	0.899314	1.197551
B	0.520222	1.461828	1.763328
C	-1.583269	-1.587090	1.598145
C	-0.791891	-1.377662	2.895827
C	-1.584648	-3.091934	1.255867
C	-3.404958	-1.246636	-1.161085
H	4.553884	-2.043042	-1.201947
H	2.598434	-3.310035	-2.052912
H	0.334692	-2.596646	-1.454077
H	-0.587601	-3.428652	0.956204
H	-2.283191	-3.346116	0.457572

H	2.408311	-2.357005	-1.307801	H	2.400988	-2.206801	-1.591622	H	-1.870258	-3.667162	2.141907
H	4.740452	-1.451521	1.031692	H	4.681576	-1.626322	1.009029	H	-1.139768	-2.086931	3.654846
H	3.773676	0.528876	2.711840	H	3.660823	0.234151	2.798394	H	-0.883293	-0.369594	3.296834
H	1.338557	2.600987	0.909329	H	1.376486	2.496930	1.073386	H	0.274556	-1.556174	2.733725
H	2.189585	2.343494	2.453471	H	2.110699	2.058787	2.634831	H	-2.624563	-1.293252	1.774200
H	0.669563	1.476695	2.105094	H	0.591899	1.289311	2.110115	H	-3.734446	-1.655090	-0.202344
H	1.730944	0.721671	-2.271268	H	1.858377	0.890402	-2.221925	H	-3.678818	-0.185868	-1.189172
H	0.596341	-0.652968	-1.479203	H	0.664736	-0.548632	-1.654158	H	-3.983735	-1.743471	-1.947318
H	-3.587811	-2.974115	0.005387	H	-3.642404	-3.042522	0.107510	H	-1.779430	0.129541	-2.912007
H	-3.803137	-1.348450	-0.653262	H	-3.931580	-1.442002	-0.584183	H	-2.125002	-1.470362	-3.553511
H	-3.924609	-2.753132	-1.715056	H	-4.040172	-2.877224	-1.607759	H	-0.485289	-1.073426	-3.033638
H	-0.665381	-1.500796	-2.850400	H	-0.819989	-1.486149	-2.807598	H	-1.699191	-2.509689	-1.370260
H	-1.957573	-0.411402	-2.367943	H	-2.180127	-0.472778	-2.336204	H	1.220692	2.206051	-1.471989
H	-2.345265	-1.885539	-3.266096	H	-2.472769	-1.967356	-3.238881	H	0.516343	0.701001	-2.081741
H	-1.498190	-3.260583	-1.326339	H	-1.592933	-3.297899	-1.295183	H	0.259062	2.213772	-2.986375
H	-1.849866	-4.468122	0.727274	H	-1.708520	-4.462462	0.905449	H	-1.100628	4.098321	-1.774014
H	-1.541032	-4.708019	2.449888	H	-1.341625	-4.577070	2.630670	H	-3.097676	3.943565	0.142084
H	-0.215766	-4.264912	1.368652	H	-0.067180	-4.149413	1.482522	H	-2.436115	1.213938	2.221003
H	-1.016210	-1.134429	3.397931	H	-0.956572	-0.917674	3.313516	H	-3.746565	0.597054	1.170598
H	-1.092202	-2.801624	3.986420	H	-0.872686	-2.542446	4.011973	H	-3.789483	2.276490	1.752377
H	0.272900	-2.272311	2.996722	H	0.394115	-1.984008	2.912062	H	0.153869	2.482734	1.187421
H	-2.629149	-2.512621	2.038391	H	-2.533816	-2.467387	2.138665	H	-0.350817	0.583155	1.540778
H	-2.804333	-0.580897	2.084050	H	-2.925435	-0.600278	1.878911	H	2.969610	2.343956	2.343827
H	-4.085175	1.441820	2.504909	H	-4.246611	1.402637	2.252493	H	5.230703	1.634192	1.639759
H	-3.571374	3.511415	1.225982	H	-3.643616	3.510275	1.071975	H	5.488678	-0.290698	0.095137
F	0.568753	-1.989948	0.405691	F	0.514263	-1.990781	0.203930	F	0.551627	1.678665	3.169537
**10^{+}-B-F (COSMO(*o*-DCB)) (NHC shift)**				**10^{+}-B-F (GP)**				**10^{+}-B-F (COSMO(*o*-DCB))**			
N	-0.829069	2.038883	-1.150759	C	2.306685	1.445396	-1.569625	C	2.294004	1.422090	-1.606353
C	-1.397633	1.188195	-0.269011	C	2.520902	0.450808	-0.586575	C	2.520542	0.450961	-0.601177
N	-2.393446	1.887297	0.317540	C	1.438437	-0.020613	0.239791	C	1.443936	-0.023528	0.232766
C	-2.434904	3.172809	-0.174361	C	0.124072	0.525107	-0.009546	C	0.122127	0.495738	-0.032062
C	-1.448870	3.269696	-1.098574	C	-0.006583	1.510797	-0.982428	C	-0.021677	1.459285	-1.025400
Si	-0.916753	-0.650600	0.055331	C	1.065772	1.986080	-1.757659	C	1.043797	1.937292	-1.809212
C	-1.874943	-1.421424	-1.426476	C	1.746304	-0.966444	1.277229	C	1.767137	-0.946548	1.289353
C	-1.474322	-0.909276	-2.815804	C	3.047863	-1.431799	1.376380	C	3.079556	-1.381836	1.395541
C	-3.215810	1.409072	1.424047	C	4.088412	-1.005919	0.531336	C	4.115789	-0.952829	0.545247
C	0.372182	1.797279	-1.948016	C	3.829662	-0.063382	-0.425293	C	3.840263	-0.034328	-0.430521
C	0.926341	-0.961448	-0.193506	B	0.731666	-1.534285	2.392656	B	0.761404	-1.521781	2.414638
C	2.061036	-0.218575	0.301449	F	1.367644	-2.527225	3.223685	F	1.447162	-2.497631	3.256860
C	3.372376	-0.653498	-0.107697	Si	-1.600635	-0.028741	0.545831	Si	-1.604363	-0.047987	0.531671
C	3.533066	-1.803065	-0.919821	C	-2.204699	-1.398638	-0.624858	C	-2.228367	-1.386038	-0.665103
C	2.446076	-2.521522	-1.337102	C	-2.522533	-0.821214	-2.013406	C	-2.530730	-0.803694	-2.053878
C	1.156532	-2.083502	-0.978999	C	-2.803156	1.448709	0.491532	C	-2.786240	1.441791	0.531645
C	4.524286	0.055886	0.307537	C	-4.198497	0.967507	0.920240	C	-4.179382	0.972180	0.981984
C	4.403383	1.157389	1.115751	C	-2.316261	2.569959	1.420258	C	-2.265197	2.545884	1.462742
C	3.124964	1.563982	1.538534	C	-1.223020	-2.571615	-0.755074	C	-1.264831	-2.575023	-0.785213
C	1.954148	0.917728	1.168380	H	4.613955	0.309072	-1.076493	H	4.617979	0.342279	-1.087335
B	0.594323	1.475948	1.826154	H	5.087416	-1.410756	0.653414	H	5.122555	-1.334453	0.679729
C	-1.619514	-1.579827	1.579241	H	3.267489	-2.152178	2.155440	H	3.320810	-2.079161	2.189227
C	-0.902175	-1.358978	2.916917	H	-1.624700	-0.398864	-2.474353	H	-1.622199	-0.405885	-2.516324
C	-1.584191	-3.088239	1.248437	H	-2.894114	-1.607642	-2.679081	H	-2.925787	-1.580467	-2.717994
C	-3.388653	-1.223925	-1.232242	H	-3.279959	-0.033838	-1.972399	H	-3.264974	0.005220	-2.010847
H	4.538714	-2.101369	-1.199650	H	-1.612708	-3.316775	-1.457698	H	-1.662676	-3.322566	-1.481103
H	2.563693	-3.407623	-1.950647	H	-1.041450	-3.061872	0.201903	H	-1.093695	-3.057850	0.178534
H	0.317156	-2.656494	-1.353480	H	-0.256225	-2.229421	-1.134337	H	-0.291954	-2.248661	-1.165015
H	-0.567963	-3.419469	1.013510	H	-3.139959	-1.778696	-0.191401	H	-3.171389	-1.751614	-0.236491
H	-2.229529	-3.352251	0.409932	H	-4.906471	1.802776	0.956107	H	-4.874969	1.816929	1.035904
H	-1.921730	-3.657526	2.119902	H	-4.168070	0.519347	1.918951	H	-4.136210	0.516080	1.976621
H	-1.274125	-2.081631	3.651722	H	-4.601300	0.218965	0.233061	H	-4.603237	0.234277	0.295523
H	-1.048005	-0.355745	3.314661	H	-2.201148	2.199198	2.444537	H	-2.140425	2.165727	2.482117
H	0.176150	-1.508022	2.811410	H	-1.350260	2.968612	1.099796	H	-1.297373	2.930956	1.131124
H	-2.671730	-1.302667	1.700707	H	-3.031679	3.399521	1.449203	H	-2.967044	3.386249	1.507509
H	-3.746413	-1.641161	-0.287677	H	-2.891065	1.851692	-0.523425	H	-2.887553	1.857795	-0.476816
H	-3.653047	-0.161443	-1.255033	H	-0.984629	1.933601	-1.180836	H	-1.005459	1.865036	-1.232305
H	-3.944975	-1.709998	-2.040702	H	0.896719	2.756338	-2.501585	H	0.863444	2.690497	-2.567939
H	-1.701983	0.154917	-2.927338	H	3.153941	1.771395	-2.164712	H	3.138165	1.750763	-2.204419
H	-2.036939	-1.439977	-3.591579	C	0.192820	-0.398747	3.454151	C	0.185380	-0.399934	3.459419
H	-0.410776	-1.053770	-3.020083	H	-1.760124	-0.438933	1.955230	H	-1.734577	-0.530737	1.917796
H	-1.688694	-2.500597	-1.412298	H	-0.272156	-2.014018	1.902045	H	-0.207889	-2.068791	1.926653
H	1.220435	2.284288	-1.468174	N	-0.784308	-0.676525	4.347020	N	-0.814684	-0.671227	4.329407
H	0.556996	0.730166	-2.004290	C	-1.081055	0.426851	5.115020	C	-1.117509	0.433475	5.093030
H	0.217631	2.201507	-2.946509	C	-0.252612	1.417318	4.702219	C	-0.271590	1.418010	4.699546
H	-1.131759	4.091497	-1.716220	N	0.528042	0.889390	3.691666	N	0.524931	0.885850	3.704741
H	-3.157786	3.890400	0.172124	C	-1.472577	-1.961555	4.410176	C	-1.522029	-1.946465	4.394068
H	-2.561075	1.083015	2.231648	H	-2.029327	-2.007464	5.344549	H	-2.159626	-1.937225	5.275001
H	-3.851286	0.588254	1.096931	H	-2.152885	-2.047599	3.561388	H	-2.127912	-2.069166	3.495305
H	-3.836023	2.233992	1.764464	H	-0.734392	-2.758187	4.365851	H	-0.800890	-2.756981	4.460549
H	0.228231	2.532308	1.313321	C	1.560650	1.657919	2.997249	C	1.571910	1.656982	3.034258
H	-0.306061	0.649431	1.692023	H	1.911156	2.436255	3.673360	H	1.876807	2.458075	3.703997
H	3.049769	2.419680	2.203641	H	2.379636	0.997080	2.730713	H	2.415913	1.009141	2.819529
H	5.283925	1.704509	1.436863	H	1.150149	2.103288	2.091747	H	1.189130	2.074754	2.103992
H	5.496685	-0.295184	-0.023189	H	-1.833968	0.408164	5.883164	H	-1.887656	0.418038	5.844299
F	0.781664	1.694008	3.251328	H	-0.139714	2.432697	5.038589	H	-0.155500	2.431948	5.039930

9 References

[S1] C. A. Reed, *Acc. Chem. Res.* **2010**, *43*, 121–128.

[S2] S.-H. Ueng, L. Fensterbank, E. Lacôte, M. Malacria, D. P. Curran, *Org. Lett.* **2010**, *12*, 3002–3005.

[S3] R. K. Harris, E. D. Becker, S. M. Cabral de Menezes, R. Goodfellow, P. Granger, *Pure Appl. Chem.* **2001**, *73*, 1795–1818.

[S4] J. S. McGough, J. Cid, M. J. Ingleson, *Chem. Eur. J.* **2017**, *23*, 8180–8184.

[S5] *Agilent CrysAlis PRO*, **2012**, Agilent Technologies, Yarnton, UK.

[S6] G. M. Sheldrick, *Acta Crystallogr.*, *Sect. A.* **1990**, *46*, 467–473.

[S7] G. M. Sheldrick, *Acta Crystallogr.*, *Sect. A.* **2008**, *64*, 112–122.

[S8] Mercury 3.9; Cambrige Crystallographic Data Center: https://www.ccdc.cam.ac.uk/solutions/csd-system/components/mercury/.

[S9] R. Ahlrichs, M. Bär, M. Häser, H. Horn, C. Kölmel, *Chem. Phys. Lett.* **1989**, *162*, 165–169.

[S10] O. Treutler, R. Ahlrichs, *J. Chem. Phys.* **1995**, *102*, 346–354.

[S11] M. Von Arnim, R. Ahlrichs, *J. Comput. Chem.* **1998**, *19*, 1746–1757.

[S12] V. N. Staroverov, G. E. Scuseria, J. Tao, J. P. Perdew, *J. Chem. Phys.* **2003**, *119*, 12129–12137.

[S13] V. N. Staroverov, G. E. Scuseria, J. Tao, J. P. Perdew, *J. Chem. Phys.* **2004**, *121*, 11507.

[S14] F. Weigend, R. Ahlrichs, *Phys. Chem. Chem. Phys.* **2005**, *7*, 3297–3305.

[S15] K. Eichkorn, O. Treutler, H. Öhm, M. Häser, R. Ahlrichs, *Chem. Phys. Lett.* **1995**, *242*, 652–660.

[S16] K. Eichkorn, F. Weigend, O. Treutler, R. Ahlrichs, *Theor. Chem. Acc.* **1997**, *97*, 119–124.

[S17] M. Sierka, A. Hogekamp, R. Ahlrichs, *J. Chem. Phys.* **2003**, *118*, 9136–9148.

[S18] F. Weigend, *Phys. Chem. Chem. Phys.* **2006**, *8*, 1057–1065.

[S19] S. Grimme, J. Antony, S. Ehrlich, H. Krieg, *J. Chem. Phys.* **2010**, *132*, 154104.

[S20] S. Grimme, S. Ehrlich, L. Goerigk, *J. Comput. Chem.* **2011**, *32*, 1456–1465.

[S21] A. Klamt, G. Schüürmann, *J. Chem. Soc., Perkin Trans. 2* **1993**, 799–805.

[S22] A. Klamt, *J. Phys. Chem.* **1995**, *99*, 2224–2235.

[S23] S. Grimme, C. Bannwarth, S. Dohm, A. Hansen, J. Pisarek, P. Pracht, J. Seibert, F. Neese, *Angew. Chem. Int. Ed.* **2017**, *56*, 14763–14769.

[S24] S. Grimme, *J. Chem. Theory Comput.* **2019**, *15*, 2847–2862.

[S25] P. Pracht, F. Bohle, S. Grimme, *Phys. Chem. Chem. Phys.* **2020**, *22*, 7169–7192.

[S26] C. Bannwarth, S. Ehlert, S. Grimme, *J. Chem. Theory Comput.* **2019**, *15*, 1652–1671.

[S27] F. Eckert, A. Klamt, *AIChE J.* **2002**, *48*, 369–385.

[S28] A. Klamt, *WIREs Comput. Mol. Sci.* **2011**, *1*, 699–709.

[S29] A. Klamt, M. Diedenhofen, *J. Phys. Chem. A* **2015**, *119*, 5439–5445.

[S30] A. Hellweg, F. Eckert, *AIChE J.* **2017**, *63*, 3944–3954.

[S31] J. P. Perdew, *Phys. Rev. B* **1986**, *33*, 8822–8824.

[S32] A. D. Becke, *Phys. Rev. A* **1988**, *38*, 3098–3100.

[S33] N. Mardirossian, M. Head-Gordon, *J. Chem. Phys.* **2016**, *144*, 214110.

[S34] K. O. Christe, D. A. Dixon, D. McLemore, W. W. Wilson, J. A. Sheehy, J. A. Boatz, *J. Fluorine Chem.* **2000**, *101*, 151–153.

[S35] J. M. Slattery, S. Hussein, *Dalton Trans.* **2012**, *41*, 1808–1815.

[S36] Schattenberg, M. Kaupp, *J. Chem. Theory Comput.* **2021**, *17*, 1469–1479.

[S37] N. Mardirossian, M. Head-Gordon, *J. Chem. Phys.* **2015**, *142*, 74111.

[S38] F. Jensen, *J. Chem. Theory Comput.* **2015**, *11*, 132–138.

[S39] NBO 7.0, E. D. Glendening, J. K. Badenhoop, A. E. Reed, J. E. Carpenter, J. A. Bohmann, C. M. Morales, C. R. Landis, F. Weinhold, Theoretical Chemistry Institute, University of Wisconsin, Madison, **2018**.

[S40] Gaussian 09, revision D.01, M. J. Frisch, G. W. Trucks, H. B. Chlegel, G. E. Scuseria, M. A. Robb, J. R. Cheeseman, G. Scalmani, V. Barone, G. A. Petersson, H. Nakatsuji, X. Li, M. Caricato, A. V. Marenich, J. Bloino, B. G. Janesko, R. Gomperts, B. Mennucci, H. P. Hratchian, J. V. Ortiz, A. F. Ismaylov, J. L. Sonnenberg, D. Williams-Young, F. Ding, F. Lipparini, F. Egidi, J. Goings, B. Peng, A. Petrone, T. Henderson, D. Ranasinghe, V. G. Zakrzewski, J. Gao, N. Rega, G. Zheng, W. Liang, M. Hada, M. Ehara, K. Toyota, R. Fukuda, J. Hasegawa, M. Ishida, T. Nakajima, Y. Honda, O. Kitao, H. Nakai, T. Vreven, K. Throssell, J. A. Montgomery, J. E. Peralta, F. Ogliaro, M. Bearpark, J. J. Heyd, E. Brothers, K. N. Kudin, V. N. Staroverov, T. Keith, R. Kobayashi, J. Normand, K. Raghavachari, A. Rendell, J. C. Burant, S. S. Iyengar, J. Tomasi, M. Cossi, J. M. Millam, M. Klene, C. Adamo, R. Cammi, J. W. Ochterski, R. L. Martin, K. Morokuma, O. Farkas, J. B. Foresman, D. J. Fox, Gaussian, Inc., Wallingford CT, **2016**.

[S41] A. E. Reed, L. A. Curtiss, F. Weinhold, *Chem. Rev.* **1988**, *88*, 899–926.

[S42] F. Weinhold, C. R. Landis, E. D. Glendening, *Int. Rev. Phys. Chem.* **2016**, *35*, 399–440.

[S43] K. B. Wiberg, *Tetrahedron* **1968**, *24*, 1083–1096.

[S44] K. B. Wiberg, *J. Am. Chem. Soc.* **1968**, *90*, 59–63.

[S45] R. W. F. Bader, *Atoms in Molecules: A Quantum Theory*, Oxford University Press, Oxford, **1990**.

[S46] AIM2000, F. Biegler-Koenig, J. Schoehnbohm, **2002**.
